# Unravelling the Long Non-Coding RNA Profile of Undifferentiated Large Cell Lung Carcinoma

**DOI:** 10.3390/ncrna4010004

**Published:** 2018-02-05

**Authors:** Sudhanshu Shukla

**Affiliations:** Department of Biosciences and Bioengineering, Indian Institute of Technology Dharwad, IIT Dharwad, WALMI Campus, Dharwad, Karnataka 580011, India; sudhanshu@iitdh.ac.in; Tel.: +91-8362212853

**Keywords:** Long non-coding RNA, RNA-Sequencing, Undifferentiated Large cell lung carcinoma (LCLC), Non-Small Cell Lung Cancer (NSCLC), diagnosis

## Abstract

Undifferentiated large cell lung carcinoma (LCLC) accounts for 2.9–9% of total lung cancers. Recently, RNA-seq based studies have revealed major genomic aberrations in LCLC. In this study, we aim to identify long non-coding RNAs (LncRNAs) expression pattern specific to LCLC. The RNA-seq profile of LCLC and other non-small cell lung carcinoma (NSCLC) was downloaded from Gene Expression Omnibus (GEO) and analyzed. Using 10 LCLC samples, we found that 18% of all the annotated LncRNAs are expressed in LCLC samples. Among 1794 expressed LncRNAs, 11 were overexpressed and 14 were downregulated in LCLC compared to normal samples. Based on receiver operating characteristic (ROC) analysis, we showed that the top five differentially expressed LncRNAs were able to differentiate between LCLC and normal samples with high sensitivity and specificity. Guilt by association analysis using genes correlating with differentially expressed LncRNAs identified several cancer-associated pathways, suggesting the role of these deregulated LncRNA in LCLC biology. We also identified the LncRNA differentially expressed in LCLC compared to lung squamous carcinoma (LUSC) and Lung-adenocarcinoma (LUAD). We found that LCLC sample showed more deregulated LncRNA in LUSC than LUAD. Interestingly, LCLC had more downregulated LncRNA compared to LUAD and LUSC. Our study provides novel insight into LncRNA deregulation in LCLC. This study also finds tools to diagnose LCLC and differentiate LCLC with other Non-Small Cell Lung Cancer.

## 1. Introduction

Lung cancer is the major cause of cancer-related deaths worldwide [1] and is generally divided into small cell lung carcinoma (SCLC) and non-small cell lung carcinoma (NSCLC) [2]. Non-small cell lung carcinoma is further divided into lung adenocarcinoma (LUAD), lung squamous cell carcinoma (LUSC), and undifferentiated large cell carcinoma (LCLC) [3]. Large cell lung carcinoma is the fourth most common lung cancer and accounts for nearly 2.9–9% of all lung cancers [4]. Lung-adenocarcinoma, LUSC, and SCLC are well-defined based on genetic and epigenetic studies; however, LCLC is not as well studied and is characterized as undifferentiated [3]. Therefore, understanding the molecular mechanisms underlying LCLC would facilitate the clinical management of this disease.

The non-coding regions of eukaryotic genomes, including those of humans, have been considered “noise” and to only have a “filling function” [5]. However, as a result of recent advancements in high-throughput sequencing techniques, a large part of these non-coding regions has been demonstrated to be actively transcribed [6]. The proportion of these actively transcribed non-coding regions increases with genomic complexity, suggesting the potentially important role of these previously uncharacterized genomic regions [7]. The transcriptions with no protein coding potential are called non-coding RNA, and non-coding RNA of 200 nt in length are called long non-coding RNA (LncRNA) [5]. Most LncRNA is transcribed by RNA Polymerase II and is processed as mRNA [8]. Recently, many studies have shown that lncRNA has high tissue and cancer specificity, potentially making it an optimal biomarker for use in therapeutics [6]. 

While the expression profile and role of LncRNA in LUAD, LUSC, and SCLC are well studied, there are no studies showing the expression pattern of LncRNA in LCLC [9]. Here, we utilized available high-throughput data for LCLC and identified the expression profile of LncRNA in these neoplasms. We also compared the LncRNA profile of LCLC with LUAD and LUSC to identify specific biomarkers. This study will prove useful in further characterizing this understudied lung cancer subtype.

## 2. Results

### 2.1. Long non-coding RNA Expression Profile of LCLC

**Figure 1:**
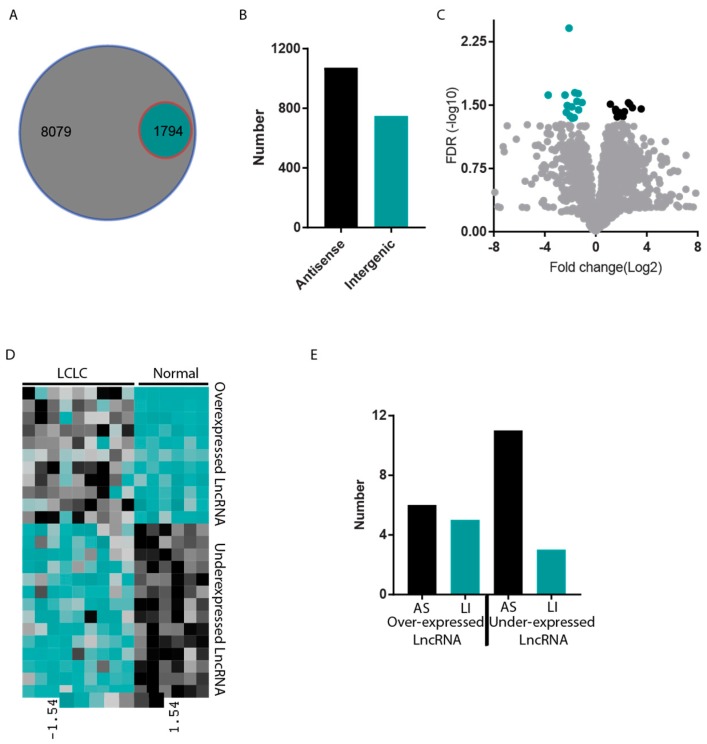
Expression profile of long non-coding RNAs in large cell lung carcinoma (LCLC). (**A**) Venn diagram presenting total number of annotated LncRNA and expressed RNA. Big circle represents total number of LncRNA annotated and smaller circle represents the expressed LncRNA in LCLC. (**B**) Bar diagram showing number of intergenic and antisense LncRNA in expressed LncRNAs. (**C**) A volcano plot to show the significantly differentially expressed LncRNA in LCLC. Black dots show overexpressed genes and blue dots show underexpressed LncRNAs. (**D**) Heat map repressing the expression pattern of significantly differently regulated gene in LCLC. Color bar indicates the expression level with black high expression and Blue low expression. (**E**) Bar diagram to show the number of Antisense (AS) and Long intergenic differential expressed in LCLC.

To begin, we downloaded the RNA-seq data from GEO and analyzed it to calculate the expression values for each LCLC patient. We used a 0.5 Fragments Per Kilobase of transcript per Million (FPKM) average cut-off across the patients to consider the LncRNA to be expressed. Using this cut-off, we found that only 18% of all annotated LncRNAs are expressed in LCLC samples (Figure 1A). We also found that of all expressed LncRNA, 59.4% is antisense RNA and the remaining 40.6% is intergenic LncRNA (Figure 1B). 

We then performed differential expression analysis between LCLC and normal lung samples. The analysis identified 11 upregulated and 14 downregulated LncRNAs with at least a two-fold expression difference and a false discovery rate (FDR) of 0.05 (Figure 1C). The expression pattern of all the differentially expressed LncRNAs between LCLC and normal lung samples is shown on a heat map (Figure 1D, Supplementary Table S1). Interestingly, over-expressed LncRNAs were evenly distributed between antisense and intergenic LncRNAs (6 antisense and 5 intergenic); however, antisense LncRNAs were enriched in underexpressed LncRNA compared to intergenic LncRNAs (11 antisense and 3 intergenic) (Figure 1D). 

**Figure 2:**
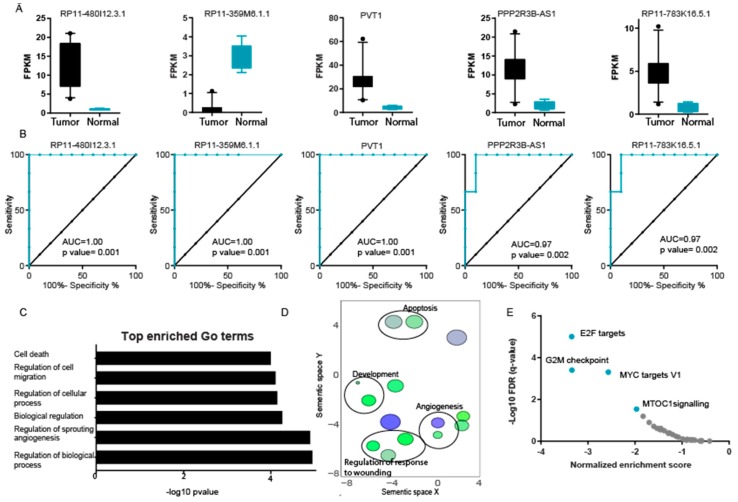
LncRNAs regulate signaling in LCLC. (**A**) The Box plot representing the expression level of indicated LncRNAs. The whiskers show the 10th and 90th percentile of expression. (**B**) Curve representing results of ROC analysis performed to show the sensitivity and specificity of mentioned LncRNA in LCLC vs. normal. (**C**) The bar diagram to show the significantly enriched Gene Ontology (GO) terms as identified in the GO analysis of best correlating genes. (**D**) Gene Ontology enrichment analysis for protein- coding genes co-expressed with differentially expressed lncRNAs with visualization by REViGO algorithm [10]. (**E**) Scatter plot showing significantly enriched data sets in Gene Set Enrichment Analysis (GSEA) using protein- coding genes co-expressed with differentially expressed lncRNAs [11].

To utilize differentially expressed LncRNAs as diagnosis markers in LCLC patients, we used the expression pattern of the top five differentially expressed LncRNAs and performed a ROC analysis. The expression pattern of the top five differentially expressed LncRNAs is shown in Figure 2A. Of these, four were overexpressed and one was underexpressed. The ROC analysis revealed that the top three differentially expressed LncRNAs showed perfect sensitivity and specificity with an area under the curve (AUC) value of one (Figure 2B). The other two overexpressed LncRNAs also showed very high sensitivity and specificity, with an AUC of 0.97 (Figure 2B). We have identified the LncRNA patterns of LCLC patients and show that LncRNA can be used as a biomarker in diagnosing this disease.

### 2.2. Identification of Signaling Pathways Regulated by LncRNA Differentially Expressed in LCLC

We performed guilt by association analysis to identify potential biological pathways regulated by differentially expressed LncRNA in LCLC patient samples. We used a Co-LncRNA algorithm to identify the protein-coding genes co-expressed with LncRNAs in LCLC. Co-regulated genes were then used to perform gene ontology (GO) analysis. The GO analysis identified regulation of biological processes, regulation of sprouting angiogenesis, biological regulation, regulation of cellular processes, and regulation of cell migration and cell death as significant enriched terms (Figure 2C). We also summarized the GO terms using the REViGO approach, shown in Figure 2D [10]. 

To identify the signaling pathways regulated by genes co-expressed with differentially expressed LncRNA, we performed gene set enrichment analysis (GSEA) using pre-ranked analysis. The results showed that protein co-regulated with differentially expressed LncRNA was significantly enriched in E2F targets, G2M checkpoints, *MYC* targets, and mTORC1 signaling (Figure 2E, Supplementary Figure S1 and Table S2). The analysis confirms the potential role of these LncRNAs in cancer development. 

We performed guilt by association analysis to identify potential biological pathways regulated by differentially expressed LncRNA in LCLC patient samples. We used a Co-LncRNA algorithm to identify the protein-coding genes co-expressed with LncRNAs in LCLC. Co-regulated genes were then used to perform GO analysis. The GO analysis identified regulation of biological processes, regulation of sprouting angiogenesis, biological regulation, regulation of cellular processes, and regulation of cell migration and cell death as significant enriched terms (Figure 2C). We also summarized the GO terms using the REViGO approach, shown in Figure 2D. 

To identify the signaling pathways regulated by genes co-expressed with differentially expressed LncRNA, we performed gene set enrichment analysis (GSEA) using pre-ranked analysis. The results showed that protein co-regulated with differentially expressed LncRNA was significantly enriched in E2F targets, G2M checkpoints, MYC targets, and mTORC1 signaling (Figure 2E, Supplementary Figure S1 and Table S2). The analysis confirms the potential role of these LncRNAs in cancer development.

### 2.3. LncRNA Expression Is Different in LCLC Compared to LUSC and LUAD

**Figure 3:**
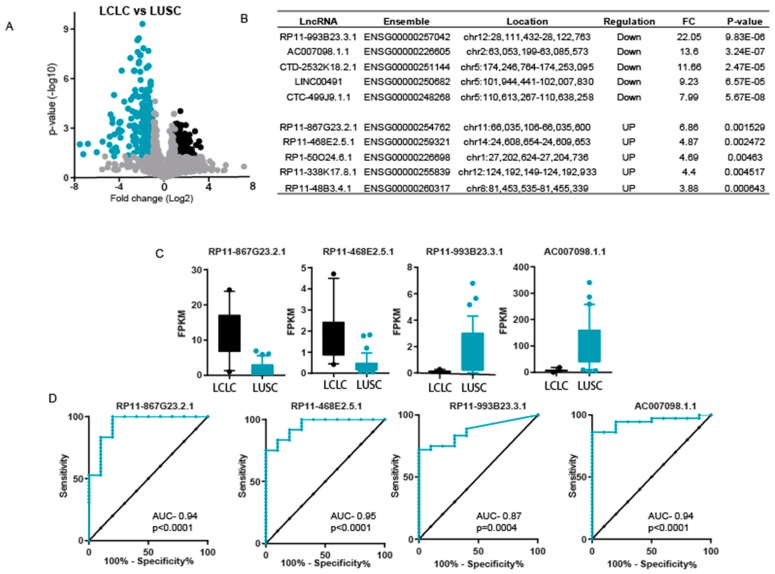
Expression difference in LCLC and lung squamous carcinoma (LUSC). (**A**) A volcano plot to show the significantly differentially expressed LncRNA in LCLC as compared to LUSC. Black dots show overexpressed genes and blue dots show underexpressed LncRNAs. (**B**) List of top five underexpressed and top five overexpressed LncRNA in LCLC compared to LUSC. (**C**) The Box plot representing the expression level of indicated LncRNAs. The whiskers show the 10th and 90th percentile of expression. (**D**) Curve representing results of ROC analysis performed to show the sensitivity and specificity of mentioned LncRNA in LCLC vs. LUSC tumors.

Generally, LCLC patients are grouped together with other NSCLC patients for treatment. Here, we wanted to identify the specific LncRNA pattern of LCLC compared to LUSC and LUAD to differentiate tumors. We compared the LncRNA expression differences between LCLC and LUSC. Interestingly, we found that there were more underexpressed LncRNAs than overexpressed LncRNAs in LCLC (228 underexpressed and 111 overexpressed), with at least a two-fold difference (Figure 3A).

The expression pattern of the top five underexpressed and top five overexpressed genes is given in Figure 3B. We used the top two overexpressed genes and the top two underexpressed genes to perform ROC analysis to discriminate between LCLC and LUSC. The expression pattern of the top 4 differentially expressed LncRNAs is given in Figure 3C. All four differentially expressed LncRNAs showed significantly high sensitivity and specificity between LCLC and LUSC (Figure 3D). 

Generally, LCLC patients are grouped together with other NSCLC patients for treatment. Here, we wanted to identify the specific LncRNA pattern of LCLC compared to LUSC and LUAD to differentiate tumors. We compared the LncRNA expression differences between LCLC and LUSC. Interestingly, we found that there were more underexpressed LncRNAs than overexpressed LncRNAs in LCLC (228 underexpressed and 111 overexpressed), with at least a two-fold difference (Figure 3A).

The expression pattern of the top five underexpressed and top five overexpressed genes is given in Figure 3B. We used the top two overexpressed genes and the top two underexpressed genes to perform ROC analysis to discriminate between LCLC and LUSC. The expression pattern of the top four differentially expressed LncRNAs is given in Figure 3C. All four differentially expressed LncRNAs showed significantly high sensitivity and specificity between LCLC and LUSC (Figure 3D). 

**Figure 4:**
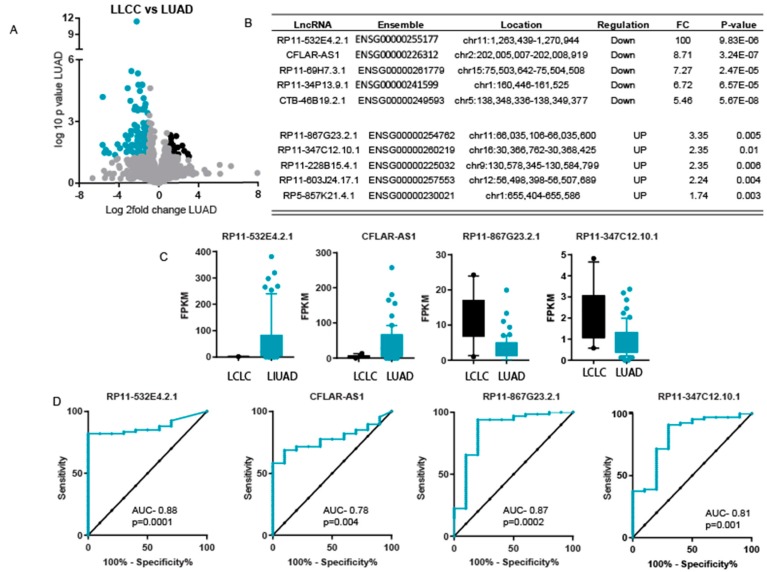
Expression difference in LCLC and LUAD. (**A**) A volcano plot to show the significantly differentially expressed LncRNA in LCLC as compared to LUAD. Black dots show overexpressed genes and blue dots show underexpressed LncRNAs. (**B**) List of top 5 underexpressed and top5 overexpressed LncRNA in LCLC compared to LUAD. (**C**) The Box plot representing the expression level of indicated LncRNAs. The whiskers show the 10th and 90th percentile of expression. (**D**) Curves representing results of ROC analysis performed to show the sensitivity and specificity of mentioned LncRNA in LCLC vs. LUAD tumors.

We also compared LncRNA expression in LCLC using LUAD samples. Interestingly. The LncRNA profile of LCLC was found to be less different than that of LUAD compared to LCLC and LUSC. We found 25 overexpressed and 109 underexpressed LncRNAs in LCLC compared to LUAD (Figure 4A). The list of the top five underexpressed and top five overexpressed genes is given in Figure 4B. To identify the markers to differentiate between LCLC and LUAD, we performed ROC analysis using the top two overexpressed and the top two underexpressed LncRNAs (Figure 4C). The ROC analysis showed that the most overexpressed LncRNA, RP11-532E4.2.1, and the most underexpressed LncRNA, RP11-867G23.2.1, showed high specificity and sensitivity between LCLC and LUAD, whereas the other two LncRNAs showed less sensitivity and specificity (Figure 4D).

## 3. Discussion

Large cell lung carcinoma (LCLC) have been classified as major lung cancer subtype by World Health Organization [12]. Although, LCLCs are very heterogeneous group of cancers, majority of LCLC patients are pooled with LUAD and LUSC for treatment [12]. Based on immunohistochemistry, two third of samples are grouped with LUAD or LUSC but remaining cases still remains unclassified [3]. Genetic aberration associate with LCLCs have been described but still there are no markers available for proper diagnosis of these patients. 

In present study, we utilized previously available high throughput RNA-seq data and performed different statistical analysis to get a novel insight in LncRNAome of LCLC patient samples. Recently, growing body of evidence suggests that LncRNA play an essential role in all aspects of biology including cancer development and progression. There are multiple studies done on LncRNA profile in major subtypes of NSCLC i.e., LUAD and LUSC, but there is no study done on LCLC samples. In this regard, we perform first study to identify the LncRNAome of LCLC samples. In our analysis, we identified that only a small fraction of all annotated LncRNAs are expressed in LCLC samples. Interestingly, the LncRNA expressed in LCLC had a higher fraction of antisense LncRNA compared to Long intergenic RNA (LincRNA). Surprisingly, this bias was more evident in underexpressed LncRNA in LCLC, suggesting that antisense LncRNA may have different regulation than the LincRNAs.

In this study, we also showed that LncRNAs can discriminate between cancerous and normal samples with very high sensitivity and specificity. Due to high sensitivity and specificity, LncRNAs can be potential marker for the development of novel diagnostic markers for the LCLC neoplasm. In guilt by association analysis, we found that the proteins co-regulated with differentially expressed LncRNA in LCLC have major role in cancer development. These genes were enriched in apoptosis, angiogenesis and other important cancer related pathways. Also, in GSEA analysis we found that these co-regulated protein coding genes played significant role in E2F pathway, G2M checkpoint pathway, MYC pathway and MTORC1 signaling pathway. Suggesting that these LncRNAs play an important role in development and progression of LCLC.

The main limitation of this work is the number of LCLC samples used for study. Although this is the largest dataset available till date, inclusion of more samples would make statistics more significant. Also, it would be interesting to see if LCLC profile changes with addition of more samples.

Due to lack of biomarkers majority of LCLC patient get classified as LUAD/LUSC and get similar treatment. In our attempt to identify markers associate with LCLC, we compared the expression profile of LCLC patients with LUSC and LUAD patients. Interestingly, we found that LncRNA profile of LCLC patients is more similar to LUAD compared to LUSC, suggesting similarity in LUAD and LCLC development. We also identified several LncRNAs which can be used to separate the LCLC samples with other non-small cell lung tumors like LUAD and LUSC with very high specificity and sensitivity. 

## 4. Materials and Methods

### 4.1. RNA-Seq Data Acquisition and Analysis

We downloaded the sequencing data from GEO (GSE66729) and analyzed as described before [11,12,14]. In short, obtained sequence were aligned to genome using Tuxedo pipeline [15]. First, reads were mapped using TopHat 2.1.0 [16]. Then, mapped reads were assembled to transcript in Cufflinks. At the end read counts were obtained and used for differential analysis using Limma in R [17]. FPKM value were calculated to show the individual expression level. For transcriptome analysis, gencode genome assembly was used. 

### 4.2. Statistical Analysis

Two-sided Non-parametric test was done to identify the differentially regulated genes. Z-score was then calculated to plot the heat map in tree-view software. ROC analysis was performed in Graph-pad version 7. All the tests done were two sided. Multiple testing correction was done using FDR method [18]. 

### 4.3. Pathway Analysis

To identify the genes correlating with differentially expressed LncRNA, Co-lncRNA algorithm was used. Genes with more than 0.7 correlation coefficient were used for GO analysis using GOrilla software [19]. REViGO approach was used to visualize and summaries the GO terms [10]. For GSEA analysis, GSEA 3.0 software was used. Genes were arranged in increasing order of correlation coefficient and preranked algorithm was applied. We used Hallmark gene set to identify the enriched gene sets. 

## 5. Conclusions

Taken together, this study identifies the LncRNA profile for LCLC patients. We also identify the diagnostic markers for LCLC tumors. Our study, supports the World Health Organization’s classification of LCLC as separate cancer type. Further studies are needed to identify function of LncRNA in LCLC neoplasm which may influence the clinical and therapeutic management of patients with such tumors.

## Supplementary Materials

The following are available online at http://www.mdpi.com/2311-553X/4/1/4/s1, Figure S1: GSEA plot of all the significantly enriched data sets. Table S1: Z scored expression of LncRNA used for Heatmap. Table S2: GSEA analysis results. Table S3: List of positive regulated genes with top5 LncRNA. Table S4: List of negatively regulated genes with top5 LncRNA.

## References

[1] Siegel R.L., Miller K.D., Jemal A. (2017). Cancer statistics, 2017. CA. Cancer J. Clin..

[2] Sutherland K.D., Berns A. (2010). Cell of origin of lung cancer. Mol. Oncol..

[3] Barbareschi M., Cantaloni C., Vescovo V.D., Cavazza A., Monica V., Carella R., Rossi G., Morelli L., Cucino A., Silvestri M. (2011). Heterogeneity of large cell carcinoma of the lung: An immunophenotypic and miRNA-based analysis. Am. J. Clin. Pathol..

[4] Sholl L.M. (2014). Large-cell carcinoma of the lung: A diagnostic category redefined by immunohistochemistry and genomics. Curr. Opin. Pulm. Med..

[5] Kung J.T.Y., Colognori D., Lee J.T. (2013). Long Noncoding RNAs: Past, Present, and Future. Genetics.

[6] Evan J.R., Feng F.Y., Chinnaiyan A.M. (2016). The bright side of dark matter: IncRNAs in cancer. J. Clin. Investig..

[7] Nohata N., Abba M.C., Gutkind J.S. (2016). Unraveling the oral cancer lncRNAome: Identification of novel lncRNAs associated with malignant progression and HPV infection. Oral Oncol..

[8] Ulitsky I., Bartel D.P. (2013). XLincRNAs: Genomics, evolution, and mechanisms. Cell.

[9] Wei M.-M., Zhou G.-B. (2016). Long Non-coding RNAs and Their Roles in Non-small-cell Lung Cancer. Genom. Proteom. Bioinform..

[10] Supek F., Bošnjak M., Škunca N., Šmuc T. (2011). Revigo summarizes and visualizes long lists of gene ontology terms. PLoS ONE.

[11] Subramanian A., Kuehn H., Gould J., Tamayo P., Mesirov J.P. (2007). GSEA-P: A desktop application for gene set enrichment analysis. Bioinformatics.

[12] Hwang D.H., Szeto D.P., Perry A.S., Bruce J.L., Sholl L.M. (2014). Pulmonary large cell carcinoma lacking squamous differentiation is clinicopathologically indistinguishable from solid-subtype adenocarcinoma. Arch. Pathol. Lab. Med..

[13] Balbin O.A., Malik R., Dhanasekaran S.M., Prensner J.R., Cao X., Wu Y., Robinson D., Wang R., Chen G., Beer D.G. (2015). The landscape of antisense gene expression in human cancers. Genome Res..

[14] Dhanasekaran S.M., Balbin O.A., Chen G., Nadal E., Kalyana-Sundaram S., Pan J., Veeneman B., Cao X., Malik R., Vats P. (2014). Transcriptome meta-analysis of lung cancer reveals recurrent aberrations in NRG1 and Hippo pathway genes. Nat. Commun..

[15] Trapnell C., Roberts A., Goff L., Pertea G., Kim D., Kelley D.R., Pimentel H., Salzberg S.L., Rinn J.L., Pachter L. (2013). Differential gene and transcript expression analysis of RNA-seq experiments with TopHat and Cufflinks. Nat. Protoc..

[16] Trapnell C., Pachter L., Salzberg S.L. (2009). TopHat: Discovering splice junctions with RNA-Seq. Bioinformatics.

[17] Ritchie M.E., Phipson B., Wu D., Hu Y., Law C.W., Shi W., Smyth G.K. (2015). limma powers differential expression analyses for RNA-sequencing and microarray studies. Nucleic Acids Res..

[18] Benjamini Y., Hochberg Y. (1995). Controlling the False Discovery Rate: A Practical and Powerful Approach to Multiple Testing. J. R. Stat. Soc..

[19] Eden E., Navon R., Steinfeld I., Lipson D., Yakhini Z. (2009). GOrilla: A tool for discovery and visualization of enriched GO terms in ranked gene lists. BMC Bioinformatics.

