# Supplementary material for: Unravelling the Long Non-Coding RNA Profile of Undifferentiated Large Cell Lung Carcinoma
Source: Noncoding RNA. 2018 Feb 5;4(1):4. doi: 10.3390/ncrna4010004 (PMC5890391; doi:10.3390/ncrna4010004)
Supplement: Supplementary file 1 [file ncrna-04-00004-s001.pdf]

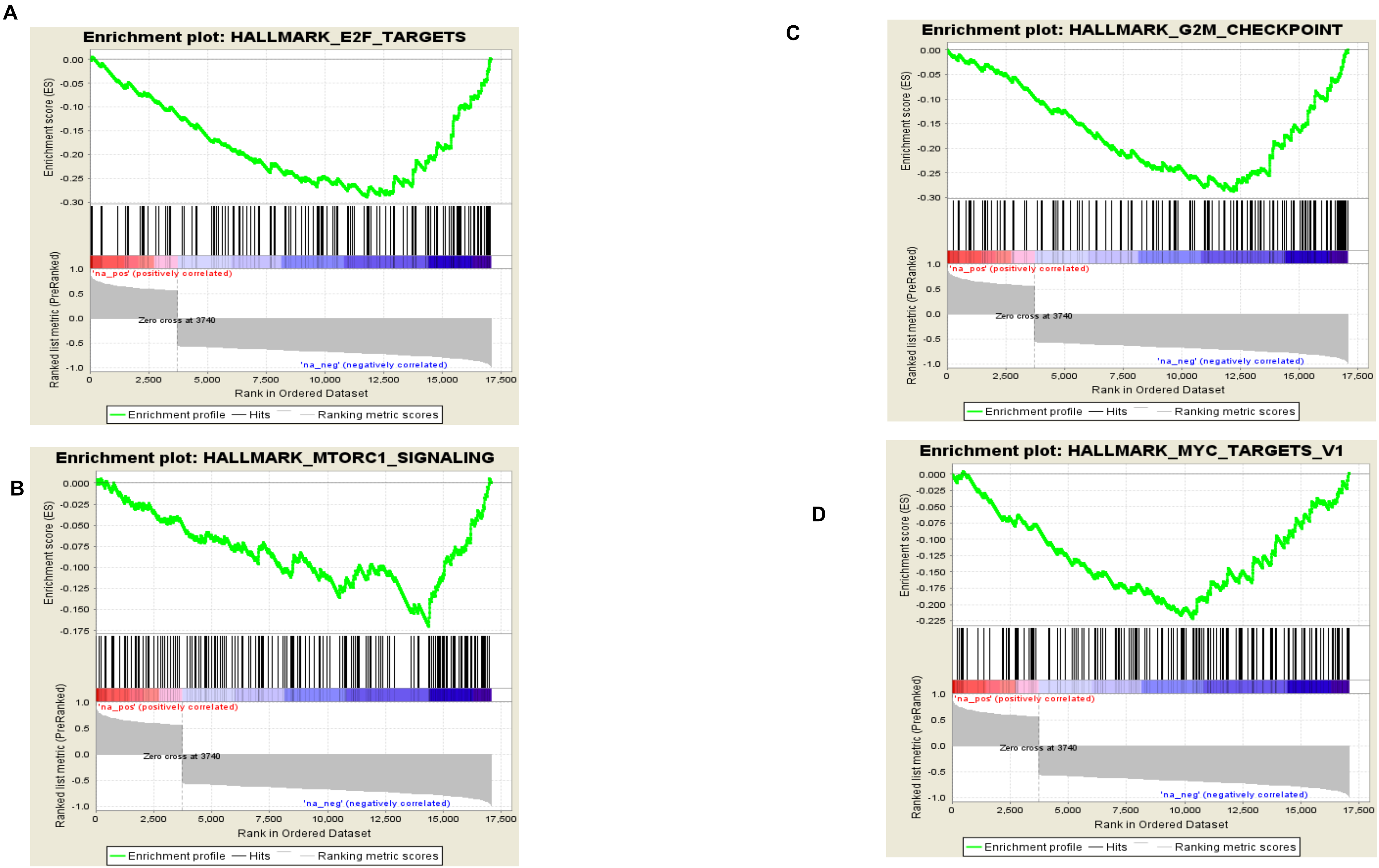

Supplimentry Figure 1- GSEA plot of all the significantly enriched data sets.

Supplimentry table 1: Z scored expression of LncRNA used for Heatmap

gene name	Large.	Large.Cell.1	Large.Cell.2	Large.Cell.3	Large.Cell.4	Large.Cell.5	Large.Cell.6	Large.Cell.7
RP11-480I12.3.1	0.86	1.92189	-0.58257	0.293378	-0.01075	0.298213	-0.05745	1.426062
PVT1	0.51	0.528962	2.805457	0.744063	-0.23242	0.946929	0.644022	-0.49832
RP11-783K16.5.1	0.5	0.801134	2.382613	0.836208	-0.88521	0.607387	-0.02162	0.007917
PPP2R3B-AS1	-0.92	0.989577	0.554721	0.319507	0.775065	0.691629	0.665095	2.30364
RP11-245J9.4.1	-0.41	0.63698	0.500735	0.486873	0.211292	1.072923	-0.02079	-1.02992
Z83851.4.1	-0.48	-0.19666	-0.0449	0.521845	-0.27177	-0.128	0.097712	-0.10842
AC074117.10.1	0.26	-0.04253	1.062745	1.612085	-0.43519	1.177709	1.104093	1.995102
RP11-539L10.3.1	0.77	0.142025	1.13385	0.555188	-0.4964	0.330995	1.493892	2.285316
RP5-1057J7.6.1	-0.46	0.677257	0.850895	0.66216	0.467272	0.463049	0.60703	1.458551
RP11-488L18.10.1	-0.49	-0.30316	-0.25469	0.31528	0.849582	1.024589	1.763832	0.656867
HCG25	1.28	0.528442	1.561257	1.442326	-0.64416	1.395538	0.231245	0.726803
CTD-3185P2.1.1	0.29	-0.82716	-1.31099	-0.29855	-0.79409	-1.21484	-1.31185	0.07118
RP11-88I18.2.1	-0.82	-1.00625	0.712026	-0.37935	-0.96714	-0.51031	-0.76271	-1.56367
RP11-1008C21.2.1	-0.37	-1.16539	-1.19518	-1.07738	-1.02855	-0.56069	0.476905	-0.99607
RP11-65F13.2.1	0.46	-1.11092	-1.33234	-0.26844	-0.6921	0.358947	-0.56037	-1.23025
RP11-361M10.5.1	-0.23	-0.3369	0.34032	-0.62329	-0.77064	-1.17209	-1.31612	-1.11021
AC008440.5.1	-0.93	-0.95175	0.933572	-1.06045	-0.83363	-0.72615	-0.79311	-0.79234
AC026150.8.1	0.35	-0.54883	-1.25287	-0.71177	-0.42918	-0.26888	-0.97498	-1.14499
RP4-575N6.4.1	-1.15	-0.2085	-1.14747	-1.14747	-0.54181	-0.69244	1.418168	-1.14747
ADAMTS9-AS2	-0.77	0.290414	-0.12756	-1.14964	-0.97121	-1.0302	-0.35149	-0.72683
AC093110.3.1	-0.71	-0.66593	-1.0675	-0.81012	-1.02493	-0.42814	0.07772	-0.50064
CTD-3107M8.4.1	-0.54	-0.95779	-0.93333	-0.81512	-0.99672	0.998548	-0.63256	-0.75457
RP11-327J17.3.1	-0.74	-0.75284	-0.81046	-1.01312	-0.75491	-0.89735	-0.54652	0.753826
SNRK-AS1	-0.53	0.222692	-0.63843	-1.02188	-1.05016	-0.85196	-0.46941	-0.85044
RP11-359M6.1.1	-0.57	-0.72405	-0.854	-0.854	-0.06374	-0.80841	-0.71755	-0.68843

Gene Name	Large.	Large.Cell.9	Normal	Normal.1	Normal.2	Normal.3	Normal.4	Normal.5
RP11-480I12.3.1	1.86	-0.25668	-0.9502	-0.9786	-1.00393	-1.0085	-0.97401	-1.00834
PVT1	0.36	0.663446	-0.77822	-0.97369	-0.98561	-1.00814	-0.8208	-0.94415
RP11-783K16.5.1	0.82	0.518555	-1.03529	-1.22476	-1.17865	-0.87361	-0.79353	-0.98579
PPP2R3B-AS1	-0.16	1.259965	-1.10756	-0.92865	-0.7028	-1.19477	-0.86479	-1.05102
RP11-245J9.4.1	0.12	0.469447	-0.77264	-0.70208	-0.9447	-0.85422	-0.48203	-0.88608
Z83851.4.1	0.82	0.084974	-0.5088	-0.70269	-0.44348	-0.69584	-0.55515	-0.59877
AC074117.10.1	0.33	-0.50947	-0.87874	-0.66406	-0.9891	-1.03763	-0.75002	-0.93837
RP11-539L10.3.1	0.75	-0.83098	-0.3658	-0.95442	-0.57632	-1.25212	-0.86593	-1.22639
RP5-1057J7.6.1	-1.06	-0.1254	-1.1267	-0.90859	-0.69803	-1.26056	-0.51733	-0.90434
RP11-488L18.10.1	0.38	1.69429	-1.50685	-0.95127	-0.72639	-1.68086	-0.32595	-0.79242
HCG25	0.39	-0.01436	-0.78401	-1.36278	-0.26269	-0.93511	-0.98009	-1.05217
CTD-3185P2.1.1	0.39	-0.47721	1.886034	1.021256	1.440811	0.518347	0.950973	0.478282
RP11-88I18.2.1	0.03	-0.05894	1.92301	1.536534	0.324327	0.767264	0.919081	0.741136
RP11-1008C21.2.1	0.21	0.003409	1.270238	1.274225	1.88468	0.876676	0.516076	0.63115
RP11-65F13.2.1	-1.03	-0.25618	0.813627	1.498033	0.919182	1.831534	0.57595	0.786737
RP11-361M10.5.1	-0.15	-0.87541	0.900546	0.588532	0.638319	1.468652	1.055952	2.053302
AC008440.5.1	0.26	-0.56657	0.665814	1.105599	0.873782	2.229403	0.959471	0.372355
AC026150.8.1	-0.6	-0.60454	1.508009	1.676522	0.843723	1.45478	0.295631	1.071462
RP4-575N6.4.1	-1.15	0.074523	1.300469	1.199934	0.781674	1.545202	0.197469	0.865233
ADAMTS9-AS2	-1.04	-0.428	0.451568	2.205524	0.607617	0.636489	1.450127	1.324714
AC093110.3.1	-0.64	-0.85709	0.565843	1.658647	1.292424	1.030729	1.245062	1.437329

CTD-3107M8.4.1	-1.02	-0.3224	0.637064	1.474784	1.599301	1.463732	0.099332	1.186605
RP11-327J17.3.1	-0.66	-0.75993	1.338793	2.170436	0.780465	1.32038	0.305247	0.71675
SNRK-AS1	-0.7	-0.74493	1.844224	1.835349	0.820992	0.817532	0.59788	1.126696
RP11-359M6.1.1	-0.85	-0.854	0.829953	1.969572	0.62322	1.497655	1.301927	1.048782

Supplimentry table 2: GSEA analysis result

GS<br>follow link to MSigDB	SIZE	ES	NES	NOM p-val
HALLMARK_E2F_TARGETS	194	-0.28945	-3.3461	0
HALLMARK_G2M_CHECKPOINT	194	-0.2876	-3.34217	0
HALLMARK_MYC_TARGETS_V1	191	-0.22178	-2.56817	0
HALLMARK_MTORC1_SIGNALING	192	-0.1703	-1.96826	0.00223
HALLMARK_DNA_REPAIR	138	-0.17421	-1.82737	0.008245
HALLMARK_UV_RESPONSE_UP	149	-0.15779	-1.69783	0.027242
HALLMARK_UNFOLDED_PROTEIN_RESPONSE	110	-0.16594	-1.60063	0.031477
HALLMARK_MITOTIC_SPINDLE	185	-0.13476	-1.53536	0.053393
HALLMARK_MYC_TARGETS_V2	56	-0.20054	-1.49627	0.076517
HALLMARK_COMPLEMENT	187	-0.12614	-1.45447	0.082497
HALLMARK_COAGULATION	125	-0.14556	-1.45201	0.093381
HALLMARK_EPITHELIAL_MESENCHYMAL_TRANSITIO	194	-0.12523	-1.45125	0.075991
HALLMARK_INTERFERON_ALPHA_RESPONSE	93	-0.15259	-1.37628	0.113043
HALLMARK_HYPOXIA	189	-0.11151	-1.29388	0.177654
HALLMARK_SPERMATOGENESIS	122	-0.12797	-1.28243	0.181273
HALLMARK_BILE_ACID_METABOLISM	102	-0.13458	-1.25752	0.205346
HALLMARK_PANCREAS_BETA_CELLS	35	-0.19214	-1.19741	0.231966
HALLMARK_IL2_STAT5_SIGNALING	184	-0.09899	-1.12328	0.313348
HALLMARK_APICAL_JUNCTION	183	-0.09535	-1.07776	0.387097
HALLMARK_IL6_JAK_STAT3_SIGNALING	81	-0.11633	-0.98355	0.47
HALLMARK_KRAS_SIGNALING_DN	180	-0.08034	-0.91568	0.555051
HALLMARK_APICAL_SURFACE	40	-0.13793	-0.89649	0.574586
HALLMARK_MYOGENESIS	177	-0.07818	-0.89429	0.598425
HALLMARK_ESTROGEN_RESPONSE_LATE	183	-0.07791	-0.89177	0.611364
HALLMARK_PROTEIN_SECRETION	90	-0.09265	-0.834	0.666667
HALLMARK_P53_PATHWAY	187	-0.07109	-0.81796	0.708241
HALLMARK_PI3K_AKT_MTOR_SIGNALING	93	-0.0882	-0.79369	0.713422
HALLMARK_FATTY_ACID_METABOLISM	151	-0.07181	-0.77155	0.729025
HALLMARK_GLYCOLYSIS	188	-0.06208	-0.73002	0.775371
HALLMARK_XENOBIOTIC_METABOLISM	175	-0.05525	-0.62188	0.892202
HALLMARK_PEROXISOME	99	-0.06284	-0.58917	0.921544
HALLMARK_ALLOGRAFT_REJECTION	181	-0.0519	-0.58112	0.928171
HALLMARK_NOTCH_SIGNALING	29	-0.07195	-0.4139	0.995536

FDR q-val
0
0
0
0.029487
0.064603
0.128571
0.196976
0.242139
0.260554
0.290112
0.266446
0.245357
0.318393
0.417215
0.407126
0.420301
0.492647
0.592258
0.646863
0.791409
0.888647
0.883958
0.849508
0.818324
0.879145
0.870739
0.874173
0.873594
0.89876
0.984548
0.980921
0.95628
0.994683

Supplementary table 3: List of positive coregulated genes with top5 LncRNA

RP11-480I1	PVT1	RP11-783	PPP2R3B-A	RP11-359M6.1.1	
RPP38	TMX2	FAM189B	MYOZ3	RAB36	
CDHR3	PICALM	ZNF217	C3	BPIFA1	
CCDC65	TOX4	BZW1	43347	APOB	
C22orf15	CHCHD4	CPSF7	CDK2AP2	KCNIP2	
KCNRG	TFG	ZFR	RGS9	IFFO1	
WDR38	PPP2R5E	GTF2F1	SIAE	ZNF540	
HLA-DRB5	GNPDA1	VEGFA	KIAA1456.	TRIM39-RPP21	
RSPH10B2	CSNK1G3	DAXX	KCNE3	CRYBA4	
SHH	SLBP	TRIM59	NME5	BPIFB2	
WFDC1	FAM189B	GPR152	TMEM50B	C12orf63	
PACRG	RABGEF1	EPS15L1	C9orf135	ITPR2	
TTLL10	PSMD8	MRPL37	GPX3	OR2L13	
DNAAF1	ERLIN1	ITPK1	GPX3	CCDC54	
FAM154B	PPRC1	PPP2R5E	AC006276.	FLT3	
FAM166B	NCS1	PLIN3	SLC22A4	ASB2	
PALM3	PRKAR2A	FTSJ1	TNNI3	RTBDN	
CAPSL	RP11-80H	C1orf212	UNC45B	TTN	
APOBEC4	API5	MAT2A	GHRL	TXLNB	
PZP	MRPS18B	ASAH2B	GRIN3B	AGAP3	
DNAH12	KIAA1161	NAA25	TMEM231	PNPLA7	
TCTE1	PFDN1	DLG5	LINGO4	PSTPIP1	
SDR42E2	METTL14	ZFP1	C13orf30	AKAP6	
C1orf87	FCF1	METRNL	AVPR2		43344
IL12B	GLRX3	QTRTD1	ORAOV1	ZNF775	
WDR96	ZDHHC3	KIAA1161	C10orf67	DNER	
AKAP14	ASNA1	RRP1B	FCGR2A	RAB33A	
C13orf26	RNF11	PSG3	RHBDL1	DISP2	
C9orf117	SAMD8	IL17RD	RP11-48B1	PAN3	
LRRC18	TOR1B	SAP130	C11orf70	PPEF2	
C11orf88	ARL8B	ATG3	H1FNT	RIMS3	
C2orf73	CAMKK2	RAD9B	DNAJC4	TBC1D10C	
PPP1R36	USP14	TADA3	TRPC6	SLC2A6	
FAM179A	PXK	NCKIPSD	BCAN	ABCB1	
C1orf129	C7orf70	ATP5S	SULT1A4	SMCHD1	
GAS2	C4orf3	AP2A1	C10orf107	PAK3	
ENKUR	ATG3	RFT1	CYS1	CDKL1	
TMEM190	DENR	PRKAR1B	KANK3	FGD2	
OSCP1	BEND3	COMMD5	ALDH3B1	RIMBP2	
CCDC108	MTPN	CDCP1	BAAT	CACNA1H	
PPIL6	RRAGC	VPS8	C16orf71	KIF19	
KIAA1377	VPS29	TFG	SERPINA1	PMEL	
TTC40	DCUN1D5	TOX4	IFT27	RBMXL2	
DYDC1	KLF10	RCOR1	RPP38	EPHX2	
KIF6	TMED2	EIF3B	HLA-DMA	GIPR	
PPP1R32	DBN1	SH3D21	TRIM49	CASD1	
SLC47A1	DCAF10	FUS	GATA5	STAMBPL1	
C2orf77	SHISA5	ZFPL1	CDHR3	ZAP70	
PIH1D2	HAUS6	DYNC1LI1	RP11-1348	PALM2	

NEK5	OTUD4	SBNO1	CRYAA	SYT7
RNF175	VEGFA	PRPF40A	C8orf73	SSR4
CCDC78	LRRFIP2	PTPRK	PSENEN	NLRP14
RP11-503N	BCL2L2-PA	RP11-80H	ODF3B	CLEC12A
LDLRAD1	SLIRP	YES1	GTF3A	KCNC4
CCDC164	KCNJ12	KIF1C	PLK5	RP11-1286E23.12.1
C20orf85	ZFP91	TMEM20.	ADHFE1	UNC79
HDLBP	CLIC1	DNTTIP1	FAM209A	GNLY
AC002472.1	EP400	METTL2B	ADH1A	SLC17A7
C6orf165	TAF3	GPR156	FAM92B	MYO7B
FBXL13	BMI1	PFDN1	RP11-505K	GABBR2
ZMYND12	DAXX	SETD8	CCDC65	BDKRB1
DNAI2	SET	SHMT1	VSIG2	UTS2
CCDC42B	KPNA4	METTL14	CINP	PTPRN2
C6orf103	C10orf46	SNTB2	CINP	ASB5
PLLP	CBS	SNTB2	SERPINF2	SMAD9
AXDND1	SNTB2	DDTL	SCGB3A2	CLEC10A
RSPH10B	SNTB2	DPF2	C9orf24	ASMT
TRIM55	TLE1	PATL1	BMP4	C19orf45
C16orf93	CDK2AP1	PSMD8	SFTPD	PIWIL4
ANKRD18B	ACTR10	VPS33A	SCGB1A1	BIN2
CDHR4	FBXW8	API5	CCDC116	FGF21
CCDC135	SLC38A2	C3orf38	C22orf15	WNT1
KRTAP5-8	SAR1A	MBOAT7	TEKT3	CNTN6
LEAP2	ARF4	ENAH	SPINLW1	BTNL2
UBXN10	ZNF283	RP11-93K	AGER	PRKCG
ZNF474	TMBIM1	SCAP	DLG4	CYP2A6
LRRC23	C9orf41	SCD	TCTEX1D1	MYOM2
WDR63	MTCH1	RNF222	DCDC5	SPESP1
DUSP13	PATL1	RAD18	ZNF536	LRRC9
TPPP3	ABCE1	CENPN	FITM1	P2RX1
HSD17B2	PRRG1	ESYT1	CCDC17	RASAL3
AZU1	PTPRK	FAM120C	PANX2	CST1
C1orf88	POLR3F	ATF2	SLC46A3	SLFNL1
CCDC151	CCNYL1	PRKAR2A	ECT2L	MC5R
KBTBD10	SLC25A26	PLEKHM2	CACNG6	LPL
ABHD1	RAP1B	MAP4	CDH19	WDFY4
CCDC87	RBBP9	DIABLO	METRN	SNAI3
PRB2	IP6K1	TCEB3B	GLRX	MIPEP
FAM183A	BEND6	PSMC4	ETV2	VSIG4
SPEF1	BZW1	NUP155	HLA-DQB1	DLGAP2
C16orf48	SCD	BCORL1	PRDM7	TMEM119
C19orf51	BICD2	HDGF	C1orf141	AC092165.4.1
XAF1	GTF2H3	FAM83G	NFKB2	GPR12
ABCB11	ABTB2	YY1	SLC25A34	DUSP26
HLA-A	RHOA	BRAP	OR1N1	ARHGAP15
SPATA17	KLF11	KCNJ12	RGS22	ARHGAP15
LRRC10B	MAP4	EIF5A2	HIGD2B	C20orf132
AC091172.1	ASB6	HIST1H2E	C9orf116	FGF9
HLA-B	BCAR3	SCO1	FBXW10	EPB42

ADAT2	ANKRD33I	GMPS	RIIAD1	LCN10
RHCE	EIF5A2	MTAP	SPATA9	CYTH4
AK7	HMGA1	FCF1	RP3-369A1	TUBB1
SPATA18	DCTPP1	RPP40	KIAA0408	HSH2D
NFE2	SAP130	C12orf43	ANXA6	SCML4
AKR7A2	ZNF395	BCAR1	AC008735.	GDA
VIP	C1orf212	PLEC	STOML3	KCNQ4
TRAF5	PABPC3	NGEF	AKR1C3	RNF128
IL5RA	SLC35F3	GTF2H3	KCNRG	CST2
ALKBH8	HIATL1	DDT	RNASE13	PHYHIPL
C1orf222	SPTLC1	C7orf70	DTHD1	SHOX
C17orf47	UBE2E1	ERRFI1	DNAH9	CXorf57
IL29	CRK	CCL28	WDR38	GPR114
CCDC81	CSNK2A1F	SPATA2	C1orf173	PRR20C
YSK4	CBL	NUDCD1	HLA-DRB5	FMO2
RP11-268F1	TEX10	MKRN2	SNTN	NEUROD2
AP000322.5	PTPLA	HIST1H2A	ARMC4	JAKMIP2
ROPN1L	RAB5A	EFNB2	SEC14L3	PRR4
MAPK15	SEC23IP	KLC2	NOTUM	FAM189A1
GSTA3	UBA6	DENR	CRB1	LIPC
LUZP2	PCDHB13	ALDOA	HLA-DPA1	SLC35D3
FHAD1	PSD2	UBA6	PODNL1	CDH17
DNAJB13	GPN3	MLEC	MORN5	FAM113B
HLA-DQA1	MMP14	TBC1D5	RSPH10B2	PLA2G12B
DYDC2	CCDC62	ZDHHC3	ANKRD1	CXCR6
C2orf62	KIAA1143	RP11-286	ZSCAN4	KCNK17
LIAS	RAB35	CAPN7	NMUR1	SNAP25
CCDC147	CENPN	CAMKK2	C5orf49	CD1D
RP11-368J2	CSNK2A1	FAM168A	C1QTNF7	GRIN2C
NSUN6	DPH3	FAM205A	SULT1A3	HBE1
C5orf20	MAPK7	NOLC1	SHH	CRLF1
ERAP2	NAA25	TMX2	WFDC1	KRTAP3-2
ABHD14A	CLIC4	PGAM5	SCN11A	MYL10
UBAP1L	DDX21	C10orf2	ABHD12B	C16orf78
CRYZL1	PLIN3	PRRC2B	RBM24	DNTT
CETN2	C22orf13	DHX33	FOLR3	NR0B2
IDI2	MCMBP	GAL3ST2	MEIG1	ARSA
ATG9B	ZBTB38	HDAC11	MYEOV	DDC
LRRC29	LRP12	FOXK1	WDR93	OLIG2
C6orf97	TBC1D13	TWF1	EPHX1	C3orf77
AC022098.1	TMEM43	ACTA1	SPNS2	PDK4
DNAJC28	COPS8	PLK2	HRASLS2	CPXM2
TPPP2	C10orf90	ASNA1	C1orf189	TSPAN11
EFHC2	ATF2	RFX8	AL359392.	IMPA2
DNAH7	BCORL1	GYS1	ORM1	NPAS4
HSF4	OLA1	ATG12	RFX2	CLEC1B
CASC1	ASAP1	GATC.1	TMEM212	OPN4
C1orf192	UFD1L	TAMM41	VWA3B	OR4C6
C17orf72	FOPNL	POM121L	UROC1	GPR15
DCST1	FOXK1	METTL16	C9orf171	OLIG3

EFCAB10	BACE2	FBXW8	PACRG	ZNF732
RIBC1	DLG5	ZNF654	GLTSCR2	TAGLN3
DLEC1	SBDS	TULP1	SHE	ITGB7
SEMA4G	IL17RD	MCHR1	MYL3	IL13
JAKMIP3	FBXW2	WDR5	TTLL10	CCDC166
C9orf174	FAM105B	C9orf40	CXCL14	RAB3C
ZMYND10	METTL16	C11orf91	HHLA2	TRIM72
MSH4	MBNL1	BCAR3	TTC16	HAND2
NUCB2	ZFPL1	PCDHGC5	DNAAF1	MUSK
LRTOMT	SETD8	RCE1	CXorf41	HERPUD1
CDRT4	C12orf23	KLHL31	C2orf71	GFRA4
C6orf118	OSGIN2	KPNA4	FAM154B	FAM19A5
GSDMD	ANP32B	RNF11	TEKT1	RP4-559A3.7.1
FSIP1	C14orf43	HCCS	OXT	IHH
RSPH1	RALA	TBX22	GUCY2D	FREM3
ZNF429	DSEL	HMGA1	TTC29	FAM155A
ALS2CR12	PRICKLE1	ZCCHC8	C11orf54	C9orf30-TMEFF1
LTN1	KHDRBS1	FBN2	LRRC46	PVALB
KIAA1841	ACTA1	AHSG	DCDC2B	PPP4R4
IGFN1	RYBP	RRP9	TMEM146	PTPRCAP
LRGUK	ESYT1	ZFP41	CPVL	SLAMF1
MOCS1	KLF7	PKM2	SPAG8	MEF2B
ZNFX1	IPMK	PSD2	TNFSF14	SLITRK2
SPA17	QRICH1	KIAA0664	FAM166B	RGS21
BTN3A2	FOXP1	RABGEF1	HLA-DPB1	IPO8
HLA-G	NPY	RRAGC	AGT	CYP3A43
CCDC153	C5orf62	GPN3	ASMTL	FRMD3
CCDC96	BNIP3L	C7orf45	DNAI1	PPP1R17
NPIPL3	PGRMC2	AK4	PRG2	CLEC3A
SULT1A1	VTI1A	SH3GL1	PALM3	OSBPL1A
EFCAB1	AP3S1	EP400	PTPRM	CHGA
SULT1C2	ENAH	CCNK	NOSTRIN	CPZ
CAPS	SLC15A4	NME2	ZNF33A	GALNTL6
CES4A	CARHSP1	TLE1	FBXW12	MUCL1
RSPH4A	GATC.1	C3orf23	TBC1D17	CHRNB3
RGPD5	GPR113	NCS1	FOLR1	NKX2-4
DHDH	GFAP	HSF1	CAPSL	DCX
NRL	INSL3	FAM129B	TSPAN19	NEFM
PLA1A	ARPC3	IL1RL2	APOBEC4	MFSD2A
C15orf26	CTDSPL	NMT2	TBC1D12	ANP32D
RP11-231C	CT62	SFSWAP	C6	AKAP4
LCA5L	SCAI	BNIP3	FAM81B	HGF
RTDR1	EFNB2	HSD3B7	TNFAIP8L2	TMEM59L
FANK1	RYR1	PVRL3	SLC27A3	PRR20D
MDH1B	ATXN2	SBDS	PZP	CABP7
TEKT2	WASL	NCKAP1	DNAH12	ZIM2
DYNC2H1	C9orf30	SLCO4A1	CNTD2	C5orf47
PBLD	C19orf67	TIMM8A	SAA2-SAA	ADAMDEC1
C1orf158	SYCE2	JRK	TCTE1	MMP25
RP11-190A	PURB	AMOTL2	SDR42E2	KIAA1755

PANX3	KLF16	TMEM42	MTBP	FGF18
IFT57	MYBL1	VPS37B	TCTEX1D4	CSF2RA
TSPYL6	NANP	PI4K2A	C1orf87	PCDH9
C9orf93	CDKN3	C11orf71	NMBR	NKX6-3
STARD9	RNF169	PLOD2	CEACAM19	ELAVL4
FAM22A	TMEM69	GNPDA1	IL12B	SLC17A9
CCDC114	C17orf39	TOR1B	ALOX15	FAM9B
CCDC88B	ZNF367	SCN5A	WDR96	NCF4
TTYH1	MIF	ZC3H15	RP11-1212	AKR1D1
PBX4	NDUFAF2	SEC23IP	RPGR	SLC38A8
RXRG	SDCCAG3	DNAJC24	TICAM2	GPR142
C2orf50	CENPH	UHRF1BP	NPR1	CITED4
SPAG6	TUBB6	SLC9A8	AKAP14	SERPINA10
PGCP.1	HTRA1	RALA	ROS1	GRP
FAM22E	TMEM233	DPH3	RBMY1J	RP11-1286E23.18.1
CCDC103	FUS	PSMD9	TLR2	CFC1
OR51I1	DAG1	SYNGAP1	IFIT1B	UGT2B4
RILP	PVRL3	PURB	ORM2	BTBD18
KIAA0319	TBC1D25	CARM1	ANKHD1-E	POMC
PION	GFOD1	PPP1R15A	SPATA4	FGL1
IQCC	GFOD1	UBE2C	CCR3	CTAG2
CAMKMT	DGKI	BEND3	C13orf26	PPT1
USP43	HDGF	GBE1	AC079354	CELF3
RP3-486B1(	CDCP1	SMTNL1	NKX6-2	SLC2A7
RP11-1396(	PXN	MBNL1	AK8	ELAVL3
WDR65	AC011551	KDM1B	C18orf63	RFX4
RP11-1035I	KCTD11	TBC1D15	C9orf117	RUNX1T1
SLPI	TCEB1	PLK1	LRRC18	NR0B1
GLB1L2	TMEM116	CCDC58	ATP1A2	CYP26B1
RP11-347C:	RAD9B	PCDHGB4	RP13-554N	STMN4
TEKT4	DLGAP4	AP2S1	MAL	INHA
COL18A1	FKBP5	KIAA1524	C11orf88	FAM154A
AP4B1	MMP16	MTCH1	C2orf73	DHRS7C
HERC5	EID2	RNF169	PPP1R36	ZNF642
FABP6	NUDT10	UQCRQ	CC2D2B	SETBP1
IFI6	CISD1	LYAR	FKBP6	NNAT
RP11-345J4	TAF9	BHLHE40	C10orf68	SHBG
RCN3	NLGN2	ASB6	PRAMEF12	ESR2
RGN	RELL2	ABCE1	EVX1	KCNU1
SPATS1	LSM3	DUS4L	IFLTD1	CALCA
KCNE1	MET	MED6	ALOX15B	IL17F
CHRNA3	TBC1D20	ZNF283	PTH1R	ZNF835
TTLL9	POFUT1	LRRFIP2	APOM	SFRP5
WDR86	UBE2E2	PLIN2	DGCR6	RNASE11
IQCK	AHRR	UTP11L	CTSH	SLC14A2
FAM18A	RHOF	PAQR7	BSCL2	KIR3DL3
MNS1	SPRYD7	CDT1	LIPJ	KLK13
HES5	TTC7B	ABHD5	TTC18	GP2
C1orf123	GPC1	TMEM43	EDN3	NEUROD4
LDHD	AZI2	GLT25D1	FAM179A	CTC-554D6.1.1

LRRC6	RPA3	PPP2R5D	C1orf129	RP11-548K23.11.1
OAF	DNTTIP1	POFUT1	WFDC6	DYTN
OAF	APPL1	RAB3IP	MMP17	NEUROD6
C7orf57	TBC1D15	MPP5	TXNDC3	PTH2
UBXN11	PFDN4	PCDHB13	FAM47E	NLRP13
METTL18	PGAM5	DFFA	GAS2	GC
EML6	VRK3	FAM105E	CLYBL	TAS2R1
HLA-C	TMC7	SNX21	ENKUR	AC010872.2.1
DIO1	RBM12B	SRSF9	NKD1	OR2Z1
C9orf169	TM9SF4	ANKLE2	RAB36	SPAG11B
PAQR6	ITPK1	MIF	DGAT2	RESP18
RP11-347C:	HRH1	GPC1	KCNQ2	IL22
PRDX1	VEGFC	FTMT	RSPO1	CST9
SLC35A1	MRAS	LLGL1	NKAPL	BARHL1
KLF8	ZNF585A	NPY	SAA2	HIST1H2BA
FAM22D	PHOX2A	TRNT1	ZSWIM2	OR52N5
RNASET2	C6orf57	NOXO1	HLA-F	GAGE12B
LACE1	TRIML2	C19orf67	TMEM190	MFRP.1
BTBD16	DYNLL1	HIST1H2E	CCDC101	KRTAP9-9
KLHL35	ZNF805	NIP7	SIDT2	GIF
MOGAT2	TTC28	CPA4	AC011443.	MTNR1B
NEK11	CSNK2A2	PSG2	MAEL	TNP1
MDN1	RILPL1	GFOD1	HLA-E	CARTPT
PRKAG2	FAM83G	GFOD1	NPIPL1	TMCO2
AIFM3	TNFRSF11	C1orf182	ANKRD34E	AC013269.5.1
SIPA1	SNRPC	DHX37	BPIFA1	ZNRF4
UPK2	TPM4	ART1	CD164L2	FAM47A
KPNA7	PPM1F	ARF4	C1orf227	GPR119
BIRC7	STAC	ATP6V0A	OSCP1	DEFB121
C17orf57	YWHAG	KLF16	UCN3	OR51G1
C19orf71	VPS26A	TBC1D13	FAM116B	GJA8
GRIA4	HIST1H2B.	MTCH2	FAM71F2	RP11-234B24.6.1
REC8	SPEM1	TMPPE	RP11-46C2	OR2M4
LRP2BP	LOX	IFRD2	SPINK4	GPR139
RSPH9	ODZ3	C8orf33	DES	COX7B2
SPAG17	AGFG1	CES2	INSIG2	GAGE1
MTIF3	MTDH	TRIAP1	TMPRSS7	SYT5
RABL2A	KDM1B	EXOSC3	TTR	NEUROD1
GSTK1	POLR3G	CDKN3	ADCY7	NPPB
MZF1	FUT5	TTC9C	WDR16	CFC1B
KRTAP5-10	SLC35G6	DGKI	CREB3L3	SYT4
PCSK4	ITGB1BP1	RCOR2	CCDC108	BARHL2
TMC4	CBLL1	UFD1L	PPIL6	GAGE12H
CRISP2	FAM210A	OR2B6	CEP192	CALB1
KATNB1	ITPR1	RNF24	PIK3CD	SOX3
LRRC71	SERPINE1	SNAPC1	FKBP2	GAGE12D
GMPR	NPW	ARPC4	KIAA1377	KLHL6
STAC3	C4orf43	PICALM	NPAS1	ZFP42
SLC34A3	KIAA1715	ADAM11	PLIN5	SIM1
RYR3	RBM7	APPL1	PEBP4	UBE2QL1

ZBBX	CCNH	PCF11	MS4A14	CCKAR
IFT172	LRRTM1	FAM207A	CCDC11	PCSK1
CCDC19	S100A2	AHNAK2	OVCH1	SMPD3
MORN2	DPM1	PRIM1	SULT1A3.1	GAGE12E
DNAH6	HAS2	FBXW2	CATSPER4	GAGE12C
NWD1	RAB32	MYBL1	C4orf47	LRRTM3
ZNF778	SHC1	ZNF790	SPDYA	ACCN1
SAMD15	BNIP3	IER5L	CDA	SLC7A14
EXOC3L1	PPP1R10	GLRX3	PTCHD1	AGBL1
INF2	PLK2	LVRN.1	FGFBP2	POU3F3
ABCC6	ADD2	GPR113	MAP6	RP11-77K12.1.1
CYP46A1	C17orf104	MET	TTC40	GNRH2
TTC21A	DNAJC25	HIF1A	RASL10A	PHGR1
THADA	CPSF7	HIF1A	FABP3	CGA
GNL2	PPP1R3F	MRAS	CUBN	KIF5C
BHMT2	OGG1	SLC10A4	ALG9	MYBPC3
GCC2	ATE1	TM9SF4	DYDC1	UGT3A1
CPLX3	LHX6	VPRBP	LRRC48	PASD1
SWSAP1	C16orf5	CDKN2AII	DPCR1	KCNK16
EFCAB2	C16orf5	DAG1	C9orf85	C4orf50
C6orf225	PCBP3	CHAT	CCDC102A	SLC38A11
DCXR	METRNL	OGG1	AKR1C1	PHF21B
RIBC2	PNMA1	OXSR1	KIF6	KIR3DL1
ABCC10	DCBLD2	KPNA6	L1TD1	PRLHR
WRB	DCT	FAM25E	TSPY8	EPHA5
FAM54B	COL6A1	ZNF578	CFB	CRH
ZMAT1	CMTM6	CA13	PPP1R32	TBC1D8B
LRRC43	TRPC1	ANKRD33	ADH6	KCNJ3
MESP1	FUBP3	BACE2	FXYD2	SCN3A
RP11-542P2	PSIP1	CAMKV	43169	TAS2R3
CNKSR1	GLIPR1	SIGLEC11	NTN4	PTPRN
RBMY1B	TRIM7	ADORA2E	GOLGA6A	SSPN
LRRIQ1	MTX3	CHCHD4	CCDC122	KRTAP1-5
TARSL2	SMARCB1	DLGAP4	CCR9	RP11-1286E23.4.1
CAT	BBS7	HIST1H2A	CARNS1	HMP19.1
KLHL3	VPS33A	TOMM70	ABCC2	STMN2
MECR	ODZ2	C16orf5	OSTBETA.1	ASB15
UBQLNL	SLC13A5	C16orf5	MUC12	KL
MORN3	HPD	LHB	ASB4	KIAA0825
CDK10	C11orf20	PABPC3	RBMY1E	KIAA0825
ARHGAP4	BIVM	PAICS	ODZ4	GYPA
C6orf108	FXR2	FAM46B	ODZ4	KLK9
PATL2	TMPPE	HAUS6	TM4SF4	FCRLA
FAM22B	PCGF6	PPP2R4	SLC47A1	RGAG1
DNAH3	ST3GAL1	RYR1	PCSK2	CYBB
ZNF211	ADAMTSL	WDR87	BCO2	INSM2
MST1	SNX12	NOC4L	C21orf62	LRFN5
RPL4	ZNF568	PHF13	MUC5AC	ZNF684
SRPK3	HSF1	CSNK2A1	MUC5AC	KIR2DL1
C11orf52	FOXL2	SNTA1	SPRYD5	DCAF12L1

KIAA1407	MRPL49	PNMA1	PDZD9	TRIM49L1
IQUB	EHD2	TRIP6	APOH	ANKRD6
ZNF600	GDF6	EIF2A	FGF19	PHOX2B
C1orf194	CILP2	MAMSTR	C2orf77	GAGE12J
PPP2R5A	MBTPS2	CDK19	CLEC12B	RLBP1
TARBP1	ZNF385D	SLC13A5	HLA-DRA	INSM1
QRICH2	ASAH2B	SUMO3	BPIFB1	C10orf54
NOL3	DIABLO	C7orf29	A1CF	MYCL1
RGS11	CHRM4	SERPINE1	CHI3L2	NBPF4
ACTR3C	TGIF2LX	PPM1F	LECT1	OTOP2
HYDIN	MTAP	C3orf26	C20orf26	TDRD6
SIL1	DNAJC24	MYBBP1A	SLC6A18	CDK5R2
ZNF775	GJC1	DBN1	RBM44	IL17REL
CCDC41	METTL11A	BICD1	SULT1C3	TRIM9
KRTAP5-9	RP11-630.	RFPL1	BMP15	CD79A
BTN3A3	FSCN1	GRIK4	FSCB	DIO2
RBKS	FAM194A	NDUFAF3	C19orf75	UMOD
TTLL3	STX1B	CGB5	PRNT	TPH1
DNAH2	DHFR	TCTA	PPP1R3A	WFDC10A
PRR5	RGS20	CDKN2A	ADAD1	POU2F2
IQCD	CDKN2A	OPA3	TRIM64B	ARMS2
TMUB1	GORASP2	MRPL11	MUC5AC	PRODH2
LENG1	C9orf40	ASF1B	MUC5AC	SLC25A21
ASB18	LHB	C10orf90	ZNF33B	LCN15
OXER1	GPR78	CORO1C	FBXO40	VGF
TNNI2	C8orf33	ARSK	AC069154.	CD27
TJP3	CPT1C	ARL13B	ZNF83	C12orf53
KCNH3	PPAPDC1A	MYC	TMPRSS15	MORC1
DZIP1L	NTAN1	RELL2	BHMT	ZMPSTE24
THUMPD2	KLF12	DRAP1	GPX2	GOLGA6L2
SEC16B.1	LRRN1	C12orf52	HLA-DRB1	MBP
OBP2B	ERRFI1	BRPF1	TMEM232	SLAMF7
ZNF441	FAM127A	AHRR	CTAGE4	RP1-310O13.12.1
CLUAP1	IFITM2	SOX12	UGT1A5	BEND4
BCL7C	SRSF9	OLA1	MLKL	FSTL5
CCDC37	CAV2	TMC7	ALPPL2	SST
EPS8L1	NUDT11	MTFMT	CPB2	ZNF643
PSORS1C2	WBP2NL	KIAA0947	PTPN22	TRPM8
TMEM161A	ZNF208	TMEM23.	PIH1D2	OR7C1
FCGRT	WNK4	TM4SF19	PI16	MYF6
C6orf163	PRR16	CCDC62	CYP2F1	PNOC
NLRC5	IL11	EIF4A1	ATP5I	TM4SF5
C12orf10	ARPC4	SET	C14orf45	RFX6
ZNF132	RNF24	XRRA1	MYBPHL	GALR1
CCDC130	ZNF790	AP2M1	FAM186A	C20orf196
EIF3C	PRAF2	MRPS22	UNCX	SCN3B
MIIP	TMEM158	FAM127A	OPALIN	NEUROG2
DYX1C1-CC	PPP2R4	CCDC51	NEK5	CPLX2
VARS2	FOSL1	PXN	FAM183B	TRIT1
C3orf25	MAGEA8	FAM25A	DYNLRB2	CYP2A7

NFASC	HABP4	SPEM1	RP11-718(	RBFOX1
CCS	ZNF718	PRICKLE3	ELANE	NCF1
CFTR	FAM57A	PPP6R1	RNF175	PGLYRP1
TMEM88	NOL9	PPAPDC1	EYA4	IGFL4
C7orf63	GNG12	TSGA10	CCDC78	LILRB1
C1orf114	RMI1	ZNF787	RP11-503N	KCNA6
CCDC111	NMT2	GOLGA3	USHBP1	DERL3
MED25	DKK1	RBM14	FAM179B	SYP
TRAPPC6A	ARL6IP6	DVL2	RIMBP3C	GADL1
BEST4	CLDND2	HIF1AN	XDH	PCP4
RABGAP1L	CCDC38	HIGD1C	TSPAN7	TM7SF4
CWC15	MAML1	COPS8	AC007405.	GAGE2A
SDF2L1	SAP30	CLIP2	TMEM27	NKX2-2
ATP6V0A4	NPBWR1	MTHFD1L	LDLRAD1	CRABP1
EFHC1	OVCH2	TRIP13	CCDC33	NFYC
IK	MT1M	APLN	GDPD3	AGPAT2
RNF224	PCNP	AC01155:	FIGF	CEACAM21
USP15	TSGA10	PPP1R10	OR5B12	FKBP11
ADK	RPL24	BICD2	CCDC164	LY9
C3orf15	UBE2Z	RP4-576H	RP11-1212	KCTD4
CXorf22	HRK	TNFRSF1:	LYRM1	RP11-1280I22.1.1
TFB1M	FBXO27	RILPL1	TMEM114	HNF4A
ANK3	IQCF1	MCMBP	AKR1C2	ZIC3
C3orf32	TMEM14E	BBS7	CA4	MAPK4
NEK10	SAMD4B	UBE2V1	TMEM132	THBS4
HSD17B4	GNAI2	VAMP3	C20orf85	AMPD1
MIA2	FAM127C	GPR78	MRPS31	CPNE5
C19orf44	SDCBP	MT1A	GALM	MPHOSPH8
IRX5	PDP1	UBFD1	NRN1L	JAM3
ACSM3	MRPS30	QRICH1	HDLBP	ANGPTL3
HYOU1	LRRC3	KHDC1L	AC002472.	GAGE12F
ALDH1L1	CCNK	CARHSP1	TRIM49L2	LIN28A
APRT	PPT2	ERLIN1	NGB	IGFBPL1
TMEM213	LVRN.1	FGF11	LCN2	C8orf80
TNNC1	MNT	RPA2	DNAH10	GAGE12G
ALDH5A1	AXL	CTSA	OR52L1	CALCB
CCDC13	RP11-122.	CCNH	C6orf165	NAA16
CCDC13	DLGAP3	SYCE2	GTSF1L	FAM22F
OBSCN	ANKLE1	C9orf78	CABYR	ST18
NDUFA7	GDNF	KLC3	SLC25A28	OR51B2
DOCK6	KCNA7	KRTAP5-6	OVGP1	HTR1F
MAT1A	NGEF	PDP1	C1orf167	SLC39A5
FAM86C1	HSCB	MLXIP	CDH15	NBPF6
FOXJ1	PLEC	METTL11	FBXL13	DEM1
FAM188B	SH2D5	PSMD6	TEPP	SMAP2
NDUFS7	TTC34	PPRC1	B3GNTL1	TAC3
FUT2	HIST1H2A	C10orf11	SLC26A9	SLC18A1
WDR35	LYRM7	GBX2	ZMYND12	REG4
RNF223	GBX2	C10orf46	CAPN3	KRTAP1-3
YIPF2	BRK1	PRAMEF1	MYOM1	KCNK9

C16orf62	UBL5	STAC	CDADC1	C8orf47
C1orf63	C10orf114	GPHN	DNAI2	PGLYRP2
CLK3	FAM102A	SCARB1	CCDC42B	FAM46D
RRP12	EPC2	NADKD1	C6orf103	SGCZ
PALMD	APLN	YKT6	CST5	MNX1
NME9	GNA12	CCDC38	FAM125A	KIR3DL2
ZNF18	TH	IL28B	TMEM143	CHGB
AGAP4	UQCRQ	DNAJB12	AKR7A3	SLAMF8
C6orf70	FBXO47	CCNYL1	ALG8	TSGA10IP
ASPSCR1	GPHN	ERCC2	TINAG	TFF1
GCOM1	YWHAE	ATG9A	TPSG1	BHLHA15
ANO1	PCDHGA1	SLC9A7	C16orf95.1	NR1H3
ATAD3C	SPANXB2	HSF2BP	PLLP	BTBD17
TSNAXIP1	HAPLN1	ABTB2	PPIAL4D	MTMR7
DAK	LRRN4CL	NCAN	PTGDR2	GABRR3
TAF1C	HEPN1	C11orf95	SEC31B	OR9A2
C11orf63	NDST3	SNX15	CXorf1	TLR8
CHN2	DCAF16	CSRP3	C2orf81	CXCR3
TLR3	CPEB1	TOP3A	RP11-343C	ST6GALNAC4
VWA3A	C10orf140	GAPDHS	AXDND1	SIGLEC10
CNTRL	MT1X	WNK4	CC2D2A	MGAT4C
ZNF611	CCL28	TRIB3	SNTG2	CCBL1
CYP4A11	PRRT4	SLC18A3	NOD2	PAH
OFD1	DPF2	SLC34A1	ANKRD31	EVX2
SPECC1	PFN1	ZFR2	C19orf80	FAM123C
DDIT4L	OR11H1	DDIT4	DPP7	U2AF1L4
EIF2D	CRTAP	MAP7D3	LOXHD1	HRH3
RP11-934B9	ATP6V1C1	PRRT4	C11orf34	ATP1A4
ITGA10	MED22	TGFBI	NOTO	SEZ6L
CCNB3	PSMD9	SPATA25	MMP13	RALGAPA1
BBS2	SLC12A8	CSNK2A2	RSPH10B	GLDC
PCP2	PDYN	FBXO27	YPEL3	OR2D3
HSF2	C11orf95	TMED2	APOB	KCNN3
SPATA7	NDEL1	TUBB6	STEAP4	BAI1
GRTP1	FAM129B	ATXN1L	C16orf89	RPGRIP1
SLC47A2	VBP1	ATG7	SLC13A4	NEUROG3
AGL	TGIF2LY	ZSCAN22	ADCY4	PROP1
COA5	RRAS2	ANKRD62	LIG4	RNF157
MAN2B1	RCOR2	REXO4	TTLL6	CAP1
ZNHIT2	POLE2	RIN1	KCNIP2	OTX2
EIF5B	WFDC13	PPP1R27	TRIM55	IGFL2
HSPA4	FAM25E	KLHDC7B	ACSM2B	CAMK4
SAMHD1	TCHH	LRRN1	C16orf93	RET
CCDC159	CALB2	ARL8B	WDR69	UGT3A2
C21orf59	TGFBI	DCT	BTK	SCG2
ZNF76	PCDHB15	PCIF1	CACNG1	DAO
PEX11G	HBQ1	TGIF2LY	ANKRD18B	SEMA4D
CCDC30	HCCS	CYTH2	FUZ	C2orf83
INTS10	C20orf141	AQP11	SAP25	C7orf58
EXD3	METTL21D	TGIF2LX	UGT1A8	USH1C

VPS33B	PBX3	ZBTB12	SCTR	PTPN7
DZIP3	OR2B6	METTL10	OR52I1	CACNG2
TRIM45	ZFR2	NUDT21	ANKFN1	CACNG2
COG4	PLP2	POLD2	PAMR1	OR2L2
LMX1A	STK35	C3orf67	ANKRD24	MYH8
CCKBR	EBF3	PGAM2	CLEC4G	CACNG3
TUBGCP6	TRIB3	WDR74	KRTCAP2	RLF
NEIL1	C3orf72	MRPS30	KNG1	MMP3
CD320	ADAMTSL	HNRNPC	C19orf69	CALY
IFIT3	CCDC117	PPP1R14I	COL28A1	DDX25
ASNS	GIP	GINS3	RASGRF1	KLK14
PCYOX1L	KLHDC7B	EIF2S3	RPS6KA1	WISP1
MERTK	SPANXC	TMEM12(	CDHR4	RDH12
ZNF20	UBFD1	POLE2	RAD9A	CLEC6A
CAPN9	ITPRIP	HHIPL1	FAM184A	ASCL1
ZNF446	MBD5	ERF	CCDC135	WNT8B
LCMT1	XRN2	TNFRSF1(	KRTAP5-8	LMX1B
C10orf81	NIP7	AGFG1	MED31	TOX3
FASTK	NCAN	TBC1D22	EXOSC8	TAAR1
TEX9	RFT1	TEX13B	GAMT	ZCCHC5
SSX2	HES7	CILP2	RP11-128(	GFI1
ETFB	C9orf21	MMACHC	ANKK1	CNGA3
CCDC106	IFNE	PTPLA	OR52H1	CTPS
ENDOD1	SBNO1	SCAI	FGG	WBP4
TCTN1	LECT2	EIF5A	LEAP2	HS6ST3
GPR111	LY6G6F	SLC25A1	PAEP	TESC
PDZD7	SLC22A24	GJD4	TCEB3C	ATP11C
PCSK6	DEFB103B	CKS2	NPHS1	ZBP1
TMEM139	OR4C12	PHF15	SLC6A12	RNF17
CC2D1A	PRAMEF7	CISD1	CYP4F11	MEI1
NOMO1	LACTBL1	HPS3	COX4I2	MPP1
TRAPPC12	KRTAP6-2	KCNA7	PLEKHN1	BARX1
INTS3	CABP5	KIAA1432	HINT2	HSD11B1
KIAA1257	LIM2	FAM43B	RP11-467N	NEURL
GRAMD1C	OR10W1	APC2	ERN2	CELA1
AKD1	NEU2	43355	IFFO1	WISP2
FAM81A	C8orf86	POLR3G	ELN	FAM69B
GPRIN2	MAGEB18	WBP2NL	UBXN10	CLDN11
HMGCS2	C5orf52	C22orf13	BOLA2B	AC217771.1
DDX4	POM121L	FLII	ZNF474	NDST4
RAD52	PRR23C	C20orf14	AQP7	TNFRSF19
ZNF680	USP29	STX5	ELFN2	GP9
ZNF43	OR13C9	SAMD8	ANKRD10	FGF17
SPATA6	C6orf146	PTPN23	FAM5C	LYZ
THYN1	PRAMEF8	TTL	LRRC23	SEC11C
PIR	KRTAP11-	C9orf50	WDR63	TSHR
C9orf43	OR2T10	ZNF367	DHRS9	CCL25
DDX53	FAM71B	DCBLD2	NLRC4	NPC1L1
CENPT	OR1D2	PRICKLE1	MOBP	DNAJC6
WDPCP	EXOC4	CYC1	DUSP13	CACNG5

PSMB8	EXOC4	SLIRP	STARD10	F5
TGM5	PFN3	HN1L	EIF4EBP3	RAB39B
WFDC2	OR10A3	TRIML2	ENO4	IRG1
C22orf43	OR12D3	CCDC71	AMN	USP17L1P
RPL5	TMEM95	VTI1A	TPPP3	TMEM150B
USP17.1	IQCF5	CDK2AP1	LIPT1	FCRL5
CCDC74B	ACPT	RGS20	HSD17B2	GPR25
ATXN7L2	C20orf62	POLR2E	BTG4	KCNK10
KCNMB3	OR2A2	SLC8A2	CCDC39	ARC
NOS3	PRAMEF2	TMEM11(	GPR20	DCC
ROPN1B	DEFB136	GAPDH	ZNF540	FAR2
TRIM17	BLID	SLC35F3	AZU1	PLA2G2D
RP11-1396(	DEFB103A	C19orf26	MS4A8B	SEZ6
ANKMY1	DEFB115	OR11H1	ADPRHL1	CD247
ZNF182	TRIM42	TCHH	PARD6B	RETNLB
UNC79	MBD3L5	PRR24	OSGIN1	WNT2
PSMG3	MBD3L2	CHRM4	KCNJ16	SSTR5
CCDC24	MBD3L3	UBE2U	C1orf88	SLC6A17
DOC2A	MBD3L4	CPNE7	SGCA	MT1G
DOC2A	PTTG2	CPT1C	PIBF1	NAIP
ZNF493	C11orf91	ANKLE1	RP1-286D(	CHST12
LCN12	C1QL1	ZDHHC5	CCDC151	LRFN2
POM121L7	IKBIP	PSG5	C11orf61	RTL1
DPCD	NPB	OVCH2	IL18	MT1H
ZNF518A	SPANXD	SGOL1	KBTBD10	SCGN
CASQ1	DFFA	SMC2	ZFP3	MB
SLC3A1	CSRP3	FOSL1	KISS1	MB
AP1G2	RPP40	ATXN2	ICAM5	KCNA3
DEPTOR	ATP5S	SPANXC	CA11	RGS7
HERC6	SEMA5B	SLBP	DNASE2B	GABRB2
TREX1	NOC4L	ADM	RP11-128(	SBK2
CRIP1	PROCR	CPEB1	DSCAML1	RNF186
KRT18	HSPB6	CLDND2	ARHGEF15	COG6
PPIB	KIF1C	FAM194A	AVP	C11orf44
NUDT14	COX6A1	PPARD	MYT1L	ZNF813
TSPAN8	OLFML2A	HYAL2	ABHD1	FAM55D
CCDC146	HSPB2-C1	ZNF660	LRRC49	DPYSL5
MDGA1	C7orf29	SMARCC1	SUN3	TMEM196
NBAS	NUP155	CPNE8	ZDHHC11	FAM178B
FBXO16	HS3ST3A1	NSFL1C	NOTCH4	DNAH17
BIRC6	BTF3	ADD2	NAPSA	NOL4
KIAA1009	ZBTB2	TMEM38.	TNFRSF4	FGF16
EIF3CL	PCBP2	VMP1	C1orf162	SFRP1
FAM50B	ZNF217	COL11A2	LGALS12	ZNF560
SLC25A27	CCBE1	ANO10	PP2D1	KCNH6
CCDC82	THOC7	BRS3	EEF1A2	KPRP
ZNF165	ANKRD60	IQCF1	ATP2C2	MCF2L
EIF3I	OR2B2	RAB32	ZMYND17	HTR1A
SYT3	12-Sep	HRH1	SYNE1	KRT40
BAI2	C16orf72	IFNE	CHRND	SALL3

FANCL	TRIM35	ZNF880	CCDC87	TEX14
PCYT2	GRIK4	CCIN	TREML1	C1orf95
CWH43	ARSK	SLC35G6	NAPRT1	NCAM1
CCP110	IL28B	MEX3D	GNPTG	NRCAM
MZT2A	CGB5	MMP16	PRB2	HIST2H2AB
ZNF266	C3orf14	CCDC85B	CCDC48	KLHL33
SH3YL1	CHRDL1	NLRP10	SCUBE1	ZDHHC22
RP11-1396(	PXMP2	SNAI1	SCUBE1	ANKS4B
EPM2A	C5orf51	SEMA5B	C12orf69	FCGR2B
RPL3	ACCN2	ZNF334	FAM183A	C4orf49
PARP14	TMEM38E	MKNK2	ZNF321P	SYT13
AC104809.:	FAM25A	CHRNB1	NPFF	LRRC7
CCDC74A	KLHL4	HAPLN1	LRRN4	PAX4
ACY3	PLOD2	THAP4	CXorf23	PCSK1N
ENOSF1	LAMP1	C3orf72	GLB1L3	MEP1B
CDH6	UBE2V1	AZI2	SPEF1	TAL2
GSTM2	MTCH2	OR1F1	SH2D3C	DSCAM
KIAA1683	CDH24	HBQ1	GBP2	MRGPRG
ZNF784	S100A3	HES3	CHP2.1	TFAP2D
SOD1	GJC2	PARS2	C19orf76	RPS26
PPP1R7	HDAC11	IP6K1	NOVA2	DLL3
DKKL1	VMA21	CT62	NUDT5	PRUNE2
C19orf45	KCNB1	SH2D5	TECR	HFM1
CYBA	GAL3ST2	DLGAP3	MYOZ2	LCN8
C22orf32	RP11-613I	MMP14	C16orf48	CADPS
FRMPD2	ADAMTS6	HPRT1	NUBP1	ANKRD30BL
POLL	CAMKV	HRK	CHODL	CPE
MPHOSPH1	ZNF599	LYRM7	C19orf51	KCNC2
ENGASE	CBWD5	ST3GAL1	OR52K2	GZMM
PPP1R16A	TEX13B	ACOT6	XAF1	OR10A4
NRG4	CCDC73	TTC34	RP1	ASXL3
ARHGAP8	OR2S2	GPR143	PLG	TMEM74
PHKG2	HIF1A	PCDHGA5	MACROD1	CNTN4
TTC26	HIF1A	PEX13	INO80E	DCLK2
CCDC89	PI4K2A	CEP55	BAIAP3	RCBTB1
C9orf173	MRPL37	NDRG1	ALDH3A1	PLSCR5
PARP10	C9orf47	ZNF717	HPCAL4	OIT3
CRELD1	PNLIP	CNTLN	NACA2	POU4F2
RP11-1396(	WDR5	DDX56	KLHDC1	ANK2
ZNF23	UBQLN1	ZNF605	RBM26	IL17D
CR1L	FAM126A	L1CAM	SCN1B	PBOV1
IDE	PCF11	CGB7	FAM178A	SLC8A3
BBS4	GLT25D1	SNAP29	EPDR1	BRSK2
RP11-1396(	CGB7	SPANXB2	AMT	LDHAL6B
PCCA	PTPRR	PDYN	C7orf23	PPFIA2
CARD14	TGFB1	GIP	ABCC3	NKAIN2
UGDH	FAM19A2	LDHA	C1orf170	TAS1R3
AC002365.:	TRIM59	FKBP5	PGC	CNKSR3
HGFAC	HSPB2.1	HIST1H3J	LACTB2	PDX1
FAHD2B	PLIN2	UBL5	PPYR1	RNF215

IQCG	MT1E	MRPL4	ACOXL	NKX1-1
ACADVL	DDTL	EME2	MYCBPAP	OR4C3
PDE4DIP	SF3A1	PTPN12	RPL13	FAM57B
RNF208	TMEM64	LETM2	HSD11B1L	NRXN3
STXBP3	ZCCHC8	OR2B2	RP4-811H2	SKOR1
FAM86B1	HIST1H2A	CGB1	FPGT-TNN	SLC24A3
HRC	CNTFR	TRPC1	FLYWCH1	ABAT
STRC	VGLL2	NPM1	TMEM80	SCRT2
FXYD4	SPANXA1	C2orf16	ABCB11	ANXA10
PIP	TWIST2	RPH3A	CYP2C8	NPPA
ZNF32	FAM92A1	DCAF10	SAA1	HOXB4
ZNF451	C9orf5	LRRC61	AC008073.	GABRB1
CACNA1F	PLEKHM2	SF3B2	GPR112	C6orf174
NAE1	CHN1	UBP1	HLA-A	KCNH8
MRPS5	ANXA5	HPD	TSPO2	KLK12
RARS2	SPANXA2	C13orf16	TRPM4	FNDC7
PCYT1B	PTRF	DCUN1D5	SPATA17	ZNF81
CLEC18A	C10orf122	DKK1	ALS2CL	TRAPPC9
ARSD	LLGL1	STC1	NDUFV2	FBLL1
SLC23A1	VAT1	BRIX1	FAM180B	KCNA1
SDR39U1	YY1	MTPN	DDX26B	HOXD9
ADCK3	ZFAND3	CDC20	LRRC10B	MAPRE2
FYCO1	CUL4B	UBE2S	NR1H4	VSTM2A
C16orf55	OSMR	SLC22A24	CLEC3B	SCG3
C3orf19	MT1A	LY6G6F	WWC3	GNAZ
WDR54	TMEM240	DEFB103I	AC091172.	SIX2
CROCC	CAPN7	MBD3L3	EGFL7	CER1
ANKRD54	PANK2	MBD3L4	HLA-B	NKX2-5
CRYM	SCAMP1	MBD3L2	FBXL8	FAM169B
CCDC160	CPA4	MBD3L5	HLA-DOB	PPP1R3B
RP11-1396	COL6A2	LACTBL1	ANKRD18A	COL25A1
GLB1L	FGF2	C5orf52	TRIM39-RI	CDH7
PHKB	C17orf74	OR10A3	CSMD1	MIPOL1
DEF6	YES1	OR12D3	ADAT2	MIPOL1
TRIM62	SNX21	CABP5	C1orf177	BRMS1L
DYNC2LI1	C5orf43	LIM2	RHCE	TBL1X
WDR31	RP4-576H	KRTAP11-	IFT140	SNCAIP
ZNF273	SLC5A3	OR4C12	AK7	RHOXF2B
BTN3A1	PHF13	KRTAP6-2	ALB	OSBPL3
NBPF1	ME2	OR10W1	NPDC1	LRRC2
PPP1R21	OR1F1	NEU2	AGRP	BRCA2
ZNF177	IZUMO4	PRR23C	HAGHL	ENC1
TMEM238	MCFD2	OR2T10	PNPLA4	ZNF519
ASPG	CAV1	FAM71B	ERCC5	TOX
CCDC104	SMCR8	EXOC4	TSPAN32	TRIM67
MUTYH	PRKAR1B	EXOC4	TDRD3	KCNC1
ATRNL1	MAMSTR	TMEM95	REEP6	MTNR1A
AHCYL1	GFRA1	DEFB115	CAPN12	GALNTL5
SELENBP1	CRKL	PRAMEF7	SPATA18	SOX14
RBM20	CADM3	C8orf86	AGTRAP	ATP7B

RNF123	AHNAK2	MAGEB18	NFE2	TFF3
PSMB10	SP9	POM121L	AKR7A2	KCNB2
AC002310.1	RBM22	USP29	VIP	BFSP2
C14orf2	RRP9	OR13C9	TRAF5	DCLK1
AKR1B1	NXN	PRAMEF8	JMJD5	RP11-404P21.8.1
PLD3	MT2A	PFN3	ADAMTS8	CNR1
RWDD2B	SNAP29	ACPT	SLC4A2	SLC17A4
FAM198A	SOX30	PRAMEF2	RP11-468E	CEP128
TMEM72	STK10	DEFB136	PFN4	CPA5
C16orf86	BAIAP2L2	BLID	DHRS2	USP17L8
RP11-723O	FAM180A	IQCF5	IL5RA	GRAP2
NOXA1	C7orf45	OR2A2	ALKBH8	NYAP1
COG7	CGB1	TRIM42	BOLA2	RUNDC3A
COG7	TM4SF19	C6orf146	SPACA3	FBXW7
TUBB1	LETM2	OR1D2	ZNF254	KLK11
WDR73	EFCAB9	C20orf62	AMBP	CARKD
POLG	DENND5B	DEFB103A	VNN3	SLC25A31
CBWD7	CGB	LECT2	C1orf222	REPS2
PAAF1	LSM12	PTTG2	ECM1	ABRA
ICA1L	DHX37	IZUMO4	SUSD4	NAPB
PCM1	EHD1	ITPR1	C19orf66	PTMS
IFIT2	OTP	EIF2C2	EFCAB6	SEBOX
EPHA4	KHDC1L	SAR1A	OCEL1	MYT1
TNIK	SOX12	CSNK2A1	APBA1	MAPK8IP1
TTC23	LSM6	HIST2H3C	TLE6	IZUMO2
AIFM1	ARL10	SLC38A2	C17orf47	RNF148
RHOBTB1	PGAM2	EIF3G	IL29	PPM1E
NDUFV3	SMAP1	PRDM4	CCDC81	CLVS1
GSTM3	CNTLN	S100A2	YSK4	PRR20B
ALPP	C4orf46	RBM42	RP11-268F	PRR20A
KLC4	NDRG1	SMCR8	RPL28	USP17L3
WDR19	OTOP1	CBS	AP000322.	CRYBA2
CCNT2	SPOCK1	LOX	TMED6	TUSC3
BRE	TMEM38A	SPANXD	GNMT	FAM194B
FAM195A	HIST1H2B	OR2S2	FATE1	CDH9
ARRDC1	FBN2	SOX30	TECTA	FCGR3B
SAMD10	UBE2R2	PTPRR	APOBEC2	C6orf94
RP11-762I7	EIF5A	HYAL3	ROPN1L	ZMAT4
SLC35F4	TCEA1	GDNF	TYK2	SRRM4
KY	SMN2	C1QL1	SUGT1	CTD-2135J3.4.1
CCNA1	F7	FOXP1	TSKS	FRMD1
MUC6	KIAA1432	MT1E	GPBAR1	EPHA7
ZNF44	PRRC2B	ZNF295	ACCS	MAGEH1
CCDC28B	C9orf50	BCL7B	MAPK15	C1orf127
TTC30B	ARHGAP2	SLC27A4	KIF12	CD163
CIB1	RP11-585I	KIAA1143	ITGA1	FOXG1
SLC40A1	GSC2	PCDHGA6	ABCA6	KRTAP1-1
PAM	S100A4	HSD17B3	SDPR	NPSR1
TK2	MBOAT7	PA2G4	CAMK1D	CNTNAP2
TK2	HSBP1L1	PHOX2A	GSTA3	CCDC155

ENPP5	EIF3M	TRIO	OR5AU1	GSC
CEP152	PTTG1	HES7	FAM188A	CHRNB2
ZKSCAN2	CD44	ANXA2	HHATL	PM20D1
SERPINA3	POM121L	TAF3	AGAP5	KRTAP4-5
ZNF692	LRRC8A	BAIAP2L2	RLN3	SLC17A1
KRT23	FLNC	NKPD1	ECSCR	CAMK2B
CCDC76	PDE12	ZNF782	ECSCR	CSNK1A1L
FAM107A	ATP2B1	HIST1H1C	TPP2	IGFBP2
SLC28A2	GOLGA3	SPRED3	PLA2G1B	GPR37L1
FNDC5	FAM36A	NPB	TAF1D	ACTL6B
DHX57	SNAPC2	MRPS18B	SPERT	KCNJ11
RBMY1D	DVL2	TMEFF1	C11orf21	FGB
ACAT1	ZNF699	ZNF479	NEGR1	INSRR
GRM5	SPON2	ODZ2	C6orf52	CDX2
AC006547.1	PSG3	PNPLA6	SMPDL3B	GLYATL3
ZNF222	UCK1	EID2	SSBP4	MGAT5B
PGR	HHIPL1	PKN3	SULT2B1	PLCZ1
BMPR1B	C18orf54	SLC39A4	LUZP2	ZNF215
N6AMT1	LARP1	FOXL2	GPR116	BEST3
SYCE1L	CLIP2	C4orf3	SH2B1	SDK1
TSTD1	ATP6V0A2	VPS29	SLC9A5	CLDN20
ATR	MEX3C	BUB3	GPR108	THEG
CCDC40	TRIAP1	ZNF621	T	MAGEC3
CNPY3	DNAJB12	FUT5	RHOD	SLFN11
COMMD1	PPP2R5D	KLHL4	FHAD1	
GOLGA1	DFNA5	CBL	MYH11	
PGPEP1L	C3orf26	UBAP2	LMF1	
PITRM1	ST5	POLR3F	ANKDD1B	
LIPT2	HAUS2	MRPL43	SPTA1	
RWDD3	PLCD3	TNNT1	TSSK3	
TKTL1	APCDD1L	E2F4	KIAA0391	
PYROXD1	LGALS1	PES1	IL37	
RP11-407N	CCIN	ZNF724P	FGA	
FGGY	UBE2F	TBC1D20	CKB	
NEUROG1	CCNA2	PRAF2	CNGA4	
MSX1	RIN1	LDLRAD3	CCDC84	
FUK	SCAP	S100A4	DNAJB13	
PKHD1L1	ZNF782	GPR107	ZNF573	
CYP1A2	ARHGAP2	COQ5	ZNHIT1	
ZNF705D	PRSS3	STX1B	CCDC68	
MSX2	DDT	C17orf39	DNAH1	
ZNF280D	HAPLN4	PIAS4	TDRD10	
INTS4	TTL	FOPNL	RETN	
RTKN	TIMM22	TBC1D25	HLA-DQA1	
NEFH	SLC8A2	CALB2	DYDC2	
ZNF705G	GNG10	BRK1	TMEM52	
C1QTNF8	FPGS	HSD17B1	C2orf62	
ADAP1	TLX2	POLE3	LIAS	
SERPINA6	NAA20	AC01346	TMEM219	
ZNF816	REXO4	C11orf20	ATG16L2	

GRIK1	CDK19	C19orf29	CHKB
UFC1	GJD4	CLIC1	CCDC147
GMNC	TMEM203	USP19	AGPAT9
RGR	TFDP1	PRSS3	NEDD9
ABCA7	APC2	BCL2L2-P	RP11-368J21.2.1
SCGB1D4	CIC	FAM72A	GPR61
PLIN1	NSA2	DDX21	FBXL16
INSL6	FKBP1A	SPTLC1	MYOCD
SCGB2A2	SGCB	COL6A1	TSPAN1
PDILT	SLC4A1	SHISA5	RGS14
CT45A4	SLC35A4	FAM127C	NSUN6
OLFM4	SNTA1	RPA3	AKR1B10
TRH	CTH	C10orf14	C5orf20
GALP	C9orf89	LHX6	CLCNKB
FOXB2	DAZAP1	DEK	IGSF22
ARGFX	BTBD19	ADAMTSL	ERAP2
PRAMEF14	FGFR1	CCNA2	ALDOB
LY6G6E	NECAB1	CGB	PTP4A3
KRTAP10-8	SIGMAR1	AXL	LY6G5C
F9	NCKIPSD	KIAA1715	C1orf116
SLC26A3	USP22	SLC7A10	ABHD14A
CFHR5	PCDHGC5	PRR16	UBAP1L
MOS	KLHDC10	YBX1	PLCG2
OR8B12	PSMF1	SAMD4B	RP11-73M18.2.1
GPR101	MZT1	CLCN1	RP11-82O18.1.1
OR1C1	NUP35	S100A11	CRYZL1
CXorf66	CALU	SUGP1	CETN2
OR8B3	SNRPB	RPSAP58	TMEM31
RP11-166N	XKR3	MAF1	IZUMO1
OR5AP2	TNNT1	MXI1	GNGT2
INSL5	VPS13A	NDUFS6	HMCN2
SPINLW1-W	EID2B	A3GALT2	HMCN2
OR12D2	AP2A1	CBLL1	HMCN2
OR51D1	NADKD1	FSCN1	HMCN2
PRSS38	DRAP1	FRMD8	LYPLAL1
KRTAP10-6	C10orf47	TLX2	CRYBA4
CT47A9	PTBP3	LPAR2	SPEF2
CT47A8	ANO6	UBQLN1	KNDC1
C10orf27	PTX4	CAMK2N1	IDI2
CRYGB	CACNB4	CSNK2B	TNFRSF6B
OR6F1	IPO7	NMD3	ATG9B
RBPJL	HIST1H4H	DPM1	LRIT3
GPHB5	METTL2B	C9orf30	HPN
LCN9	SPRED3	ASPH	C4orf33
ATP1B4	PAIP1	RHOF	TGM3
CLRN1	FTSJ1	KLF11	NQO1
OR14K1	RP11-566I	DIXDC1	CNTF
CRNN	CSNK2B	IRAK1	LRRC29
OR8A1	TFAM	HIATL1	NINJ2
KRTAP10-1	SNRPB2	KLF10	C17orf46

SCNN1B	NDUFB6	KCTD11	THSD1
SCNN1B	GFPT2	NAB2	CLIP4
CT47A11	CRAT	ABHD6	C6orf97
OR13G1	CSTF3	CDH24	GGN
CLDN17	MXI1	HSPB6	FAM189A2
GNAT3	SNAI1	CIC	ZDHHC11B
OR4D5	IER5L	CEP72	CSF2
OR10D3	MAPK1	HIST1H2E	FAM82A1
MS4A13	NTN5	CSNK1G3	RPL17
SLC22A6	GDF1	WNT7A	C2orf40
RP11-108L7	VPS8	TMEM14	IL31RA
OR1E2	RASSF8	PAX6	AC022098.1
KRTAP10-1	KPNA6	SNRPC	PTCH2
SEMG2	NRIP3	C10orf11	DNAJC28
DUXA	TPGS2	GJC2	ZMAT5
OR8D1	UBE2C	ZNF664	MUSTN1
CT47B1	GPR176	C13orf27	TPPP2
FGF3	MTHFD1L	INVS	SAA4
DPRX	CPNE7	PCDHGB6	ACBD4
TPD52L3	TTC33	PLOD1	GP6
SLC15A5	C7orf60	TAB1	SEPX1
KRT25	FJX1	ANO6	LMO1
OR52A5	HEPACAM	UCK1	SYCE3
OR51J1	UBP1	MT1M	COX5B
OR6V1	C4orf51	ARHGAP2	ATPIF1
RFPL4B	RASGEF1C	LRRTM1	IL3RA
NPVF	TCEB3B	CLIC4	ARSE
NANOS2	SOCS2	CALU	ARV1
KRTAP10-4	TMEM42	HABP4	CTB-134H23.1.1
HTR5A	MAF1	THOC7	C1orf201
CRYGC	TAF9B	ZBTB11	DHRS12
C10orf53	SPHK1	RCL1	GLI1
RBMY1F	PSG2	SLC15A4	EFHC2
MYOG	BUB3	NUP35	DNAH7
KLK15	ATP5G3	ALG3	TMEM125
ACTL7A	TAX1BP3	ASPHD1	TBX2
TYR	NEURL2	KHDRBS1	ADM2
BLACE	ZDHHC8	PROM2	CDKL3
RP13-221M	TMEFF2	ZBTB38	SYF2
HIST3H3	SFSWAP	SEMA3F	TPCN1
RBMY1A1	ZNF596	SPANXA1	ALDH2
ZNF862	MKNK2	STK32C	PTPRU
ATP2B2	APOPT1	C3orf78	LRRC19
SOX17	TMEM14E	CHCHD3	HSF4
CEACAM16	SENP3	CENPH	SFTA2
CDC20B	QPCTL	SFXN5	C3orf71
UBR2	CLNS1A	SPON2	RP11-403P17.5.1
SCGB1D2	ZFP1	OSMR	ZNF181
AC226150.1	WASF1	SPANXA2	CASC1
GALNTL1	ZFAND1	COX6A1	C1orf192

SHROOM1	IFITM3	RP11-566	CPO
FMO4	YIPF5	CBWD5	C17orf72
MLXIPL	SH3GL1	THOC5	CASP4
FOXRED1	ZNF578	PPP4R2	ZNF34
GRIA2	ZNF85	SLC2A1	TMEM163
SLURP1	PRAMEF1	PTX4	DCST1
PNISR	MMGT1	HEPN1	GPX4
ZNF514	BRAF	VEGFC	FXYD5
FGF23	TWF1	ANKRD60	CYP3A5
ESR1	KDSR	KLF7	P4HTM
SCGB2A1	SH3D21	ASB1	PRDX5
TCEA2	PAWR	DCTPP1	GRIN2D
PARD6A	CAPNS1	GRPEL1	EFCAB10
MYH7B	TBC1D5	EIF1AD	MATK
PBX1	DEPDC5	GDF6	RIBC1
PBX1	NTN3	HTRA1	ZBTB32
ZNF823	HIST1H1C	ARHGAP3	NENF
LAMA1	PPTC7	GRM2	DLEC1
SON	FAM70B	SYT6	DENND2D
PCDHA6	DENND2A	RELA	BPIFB2
TXLNG	XKR6	ARMC10	FAH
DNALI1	NUDT6	TTC28	SEMA4G
SEMA3E	NR1D2	APOA2	CORO6
14-Sep	FAM43B	KCNB1	P2RY6
WIF1	NKPD1	FADS2	RASSF4
FREM2	SGOL1	TRIM71	PPP1R3E
COL2A1	SCO1	TP53TG5	JAKMIP3
SZT2	LACTB	FLNC	IFT88
HGD	TBX22	C20orf24	C9orf174
TBCK	DNAJB5	MIS18A	MAP1A
TRIM43	TPM3	ZNF891	LMTK3
GSTP1	PHF23	C5orf22	ZDHHC1
MIOS	RASA3	BASP1	COL4A3
CA10	ARPC4-TT	IFRD1	ZMYND10
SERPINA5	CHAT	RP11-724	SEC14L4
FABP2	LHX5	C4orf51	ANKRD2
RUFY4	SUMO3	DEPDC5	MSH4
RP11-1286I	CYBRD1	LRP12	BCMO1
PAQR5	FAM168A	SLC12A8	RPL18A
ZNF470	C19orf26	CAV2	RPS15
MRPS9	DNLZ	FAM72B	TACR2
LEFTY1	ART1	EFCAB9	GDF15
MIA3	PPIA	SPOCK1	ZNF267
RNPEPL1	SEC61A2	TEX10	GPR162
QARS	SLC10A4	SLC2A10	NUCB2
CDK5	RPL35A	TMEM39	C2orf74
LRRC56	AP00035C	VPS13A	TMEM208
IFIT1	QSOX2	RPSA	LRTOMT
FILIP1	SLC39A14	TH	SORBS1
SLC5A8	RELA	ZNF208	C1orf228

MAK	CKS2	NTN5	CADM1
CYP4F8	RP13-672I	S100A3	IPP
C11orf9	RBMS3	ZNF718	ANKRD22
GTF3C6	DGAT2L6	CTC-534A	LRG1
PDCL2	NUFIP1	CHAF1A	NPR2
KCNN1	VMP1	MAPK7	C21orf2
PPOX	COMMD5	CRYAB	CYP2A13
HSPH1	RAB11FIP!	ZNF736	DDX59
WDR37	NAT16	ITGB1BP1	CDRT4
ZNF700	MEX3D	RPL24	SORCS2
IGLON5	CCL8	ZCWPW1	CD101
MT3	SPATA2	NPW	GSTO2
DIRAS2	CARM1	ACTR1A	CRABP2
MSRB2	FEZF2	VBP1	CCL17
NAA11	C22orf26	PPP1R3F	DVL1
ZNF608	PAQR7	UNG	IGFALS
MZT2B	CDT1	TP53RK	ACTG2
GAL3ST3	NR2C2	CTF1	TGM4
ZNF14	NLRP10	NHEJ1	MYBPC2
ZNF440	SHISA7	PCDHB15	PHF11
CCDC121	RSPO4	PCDHGA1	C6orf118
PEX11A	METTL5	VDAC1	RAPSN
CARD18	TAB1	OR1Q1	PRTN3
FOLH1	GPR107	ZRANB3	RP11-54H19.8.1
ARHGEF9	FNTA	BMI1	TNXB
NUP133	SLC39A4	ZNF789	GSDMD
AMIGO3	KCNIP3	RBM7	GLI4
C20orf201	NUDT19	CCDC9	FSIP1
SYTL4	DNAJC18	AKT1S1	RSPH1
ACE	NAF1	ZNF114	HMHA1
UBXN4	PPP1R14E	RNF26	ZNF429
C19orf46	ATG12	CCDC15	DROSHA
ACAD10	SLC36A4	SPRN	ALS2CR12
RP1L1	SPATA25	LRRC3	TMEM180
TMEM214	ADORA2B	GTF2E1	C12orf63
CTSL2	TMEM14C	ZC3HAV1	ADAMTSL4
CYP2J2	HES3	HIST1H2E	LTN1
WDR60	TCP11L1	FAM126A	KIAA1841
PDXK	LIN52	BRAF	EMB
SAYSD1	DNAJC25-	PORCN	IGFN1
SYBU	TMEM20C	TSSK2	HIGD1B
GEMIN8	C12orf43	TRIP10	FBXO2
ZNF571	PCOLCE	TFDP2	EIF3L
COL21A1	RNF26	TBC1D2	PDE4C
SH3BGR	MAP1B	BEGAIN	LGI4
IQCE	COPZ1	MRPS35	AXIN2
RP4-559A3.	KLC3	DENND2A	LRGUK
UBA7	A3GALT2F	RAB43	OBFC1
CTAGE1	CHRNB1	SOCS2	RG9MTD3
NPRL2	PHF19	LRFN1	CELF5

IFI30	AFAP1	TSFM	SMARCD3
WFIKKN1	SEC14L2	EHD2	GPR123
OTOA	C10orf116	SLC3A2	GNG8
ZNF75D	SLC7A10	OTOP1	SLC16A5
ZNF563	C5orf41	C8orf82	TMEM130
FDXR	ARL13B	SPRYD7	MOCS1
PRSS45	C1orf182	NDUFAF2	TST
PCDHB3	MAVS	OTUD4	ZNFX1
FOXN4	SLCO4A1	MAPKAP1	SPA17
SLC24A4	FLII	NEURL2	POLD4
ADD3	CSTF2T	TMEM15[illegible]	USE1
CAND2	SLC9A7	DSCR3	MRPS15
PPARG	RAB21	SEC22A	SLC6A20
TNMD	HIGD1C	TTC33	RAPGEF3
C1orf64	RNF167	CIZ1	BTN3A2
ACSF3	MYC	CCRN4L	HLA-G
PROC	SKA1	RP13-672	ARHGAP18
COL23A1	SLC18A3	TAX1BP3	FBXO39
GPR26	ACTN3	HIST4H4	CCDC153
DMXL2	LARP1B	TSR2	CCDC96
CACNA2D3	EIF2C2	GNAT1	ITIH1
TF	C9orf78	UBE2M	NPIPL3
RFXANK	GRK1	VAT1	SULT1A1
SLC25A47	QSER1	DCAF15	ADAM33
EFCAB8	GTF3C4	GTF2IRD1	NODAL
EFCAB8	NDUFA12	CCT5	EFCAB1
EFCAB8	PEX26	HIST1H4H	C9orf128
SSTR4	ANO4	RNF212	SULT1C2
INTS8	SLC34A1	CELSR3	SPATA24
KIAA1324	ASPHD1	SLC5A10	MRPL54
NAT14	MAGOH	CDRT1	SYNM
GLB1	EFEMP2	CLNS1A	BCAM
MFGE8	THAP1	COL6A2	CAPS
MFGE8	DDIT4	CCDC63	NXF1
C1QL4	INPP5F	AC10080[illegible]	NME7
AK2	YARS2	APCDD1L	LHCGR
THEM4	VDAC2	CCDC86	MAP3K15
AC108938.[illegible]	HMGA2	BEND6	CLEC1A
YPEL4	ZEB1	ARIH2	DHRS7B
CPSF3	HECW1	PYGL	TMEM133
ZNF525	RFX8	GSS	CLDN5
PCDHB1	NUDCD2	WSB2	NHS
ZNF606	CLMP	RAN	BAIAP2
C2orf70	TP53	HIST1H4J	RADIL
C12orf74	ZYX	SEC23A	C9orf103
LRRC17	ZBTB12	MT1X	CES4A
ADAMTS9	MAG	MRPS17	FAM71D
BLZF1	SCARB1	RYBP	KLK5
USP34	NRM	HNRNPR	RSPH4A
CCDC57	UBAC2	SHC3	RGPD5

PDZRN3	EPN2	UBE2E1	HIST1H1T
CHRNG	LRRC58	MCFD2	RRAD
PUS3	STX5	HIST2H3A	DHDH
KNCN	FAM122B	XIRP1	RORC
RBP2	DEK	CSRNP1	NRL
SMAD6	RNF222	KCTD7	C19orf18
PAX8	HIST1H2A	CSPG5	PLA1A
GALT	ATG7	GSC2	TIMP4
CCT8	CTC-534A	PRPF19	NAT1
EPOR	GALNT9	RNF167	MPP3
SALL1	PCDHA13	SEC13	OSR1
RBBP6	CDC14B	METTL21	SHROOM4
PLEKHB1	ARMC10	MDK	LGR6
METTL20	DOCK3	MED10	C15orf26
CSTL1	KIAA1524	LMNB2	OXA1L
THUMPD1	PPP1R18	CHERP	UPK3A
APBB3	SMC2	KCTD10	BLVRA
TAC1	CCDC85B	ZBTB2	PDE8A
TTLL7	ZNF717	TCEA1	AC109829.1.1
SMPD2	SLC2A11	RP11-3N2	RP11-231C14.4.1
C6orf15	BET1	MBD5	LCA5L
SESN1	RBM12	SHISA7	RNF39
TGM7	KCNJ14	SRP72	KAL1
MCF2	MDK	SNRNP40	ZBTB3
PLDN	EPM2AIP1	ANXA5	RTDR1
RNF40	GPR152	DNLZ	FANK1
CIR1	ZFAND2A	DNAJC11	MDH1B
RABL5	HIST1H1A	SPRYD4	SYT15
RXFP4	ACOT6	GSG2	TEKT2
MCCD1	RNF130	CCR10	CPNE2
NUDT17	ZNF621	MRPL49	DYNC2H1
HEATR7A	RP11-293I	HRASLS	PLD4
MUT	CD300LD	DOCK3	PBLD
RHBG	SLC38A5	C17orf10	MUC5B
C2orf88	RCL1	SENP3	C1orf158
AKAP8L	HPCA	MCM4	TXNDC17
NAT8	RECK	FAM36A	RP11-190A12.7.1
STX16-NPEI	METTL10	C5orf51	SUSD2
CTD-2006C	CGGBP1	FANCC	ZG16B
TCEAL2	ERCC2	SMAP1	PANX3
ACSF2	OTUD6B	WFDC13	UCN
TMEM35	CYC1	PAPD7	LAIR2
ROPN1	VPS37B	ANKRD7	TRMT61B
ZNF841	C1orf144	DFNA5	RPL3L
UQCRC2	C11orf85	NAA35	IFT57
REEP1	ODC1	RBM12B	VSIG10L
CCDC28A	SHC3	HSPB2-C1	CKLF-CMTM1
FAM111A	BRS3	BNIP3L	VCX2
SYT8	CTSA	RP11-196	LONRF3
ASUN	CCRN4L	MPHOSPI	TRADD

CALML4	CORO1C	SWI5	PRODH
UGGT1	CHTF8	MTX2	ESYT3
CDRT15	CLTA	C18orf54	TSPYL6
FAM156B	ANKRD7	ZNF568	NUP214
MLNR	PLEKHG2	PXK	PAM16
ANXA9	LMNB2	MAFG	FBXL22
LPIN3	GTF2F1	STEAP3	C9orf93
ZNF678	UHRF1BP1	RUVBL1	MIOX
SERTM1	C5orf34	WDR55	NHLRC4
STX16	TRIP13	RBM22	BDNF
MOGAT1	FAM46B	DHFR	NDUFS8
LRRD1	SNX15	CDC37	THNSL1
POTEF	CCR10	QPCTL	KIF27
RP3-402G1	NUDCD1	TAF5	PUS10
TATDN3	TATDN1	PCBP3	MAPK10
CDC42BPG	SEMA3F	SP5	43161
ADCK5	RP11-724	AP00035	C22orf23
CLCC1	CIDEA	STAU1	PRR14
PLEKHH1	ZNF605	TSR1	EFHB
NPHP3	ZDHHC14	ETF1	IRX3
DEGS2	TRIO	CAV1	KBTBD3
PRAMEF19	TBC1D19	HPCA	STARD9
SCNN1G	MCHR1	DMKN	FAM22A
RBM34	SDHB	KHSRP	RP5-862P8.2.1
BST2	TMEM120	GIPC3	ETV7
ARMC5	ZFP41	HSD17B1	FBXO15
SIRT7	TPST2	MAGEA8	C21orf63
ZNF425	SPIN1	ATP6V1C	NLRP1
SMYD2	LDLRAD3	C9orf47	CCDC114
CLDN22	HSD17B3	PIAS2	DOK7
TIGD2	MFAP5	RHOA	CCDC88B
H2BFWT	C11orf58	EIF3M	TTYH1
KIAA1107	ARF6	BCL2L1	RPL18
SLC11A2	KIRREL	BIVM	PBX4
ISG15	C12orf24	RP11-613	UNC93B1
POMGNT1	CD163L1	ANO7	NDST2
MOV10	TSSK2	NLGN2	SYTL2
OSBPL9	PCDHGA5	DYNLL1	SLC16A11
WDR49	DCTN4	MELK	TRIM31
RGSL1	YAP1	RPP14	CATSPERG
COL9A1	FTMT	ZBTB9	RXRG
NBPF3	C1QBP	ZNF385D	FOXL1
C16orf90	MED10	RRS1	SLC6A16
CCDC67	KIAA1644	FUBP3	C8orf46
FAM149B1	FBXO34	NUDT11	C2orf50
EYA2	FBXO34	MME	CACNG4
SETD4	SCN5A	WWTR1	CXorf65
GSTM4	PRKAA1	EID2B	MTMR11
TXNRD2	TRIP10	PVR	ZNF558
RP11-683L2	RAB3IP	APOA1	SPAG6

CDK11A	ERF	CWF19L1	PTPN6
UACA	SGTB	UBXN1	ZNF843
KATNAL2	HSD3B7	ODC1	PGCP.1
RP13-221M	TRIP6	PTGER1	ESD
EPRS	GPR156	TPM4	F10
FAIM	BCL2L13	ODZ3	CLDN15
FAM3A	PDCD5	GFAP	FAM22E
HOMER2	CAMK2N2	PXMP2	ZNF720
FHIT	PRRC1	PFDN4	CHRM3
NKTR	C20orf30	GNAI2	MVP
KRTCAP3	CAST	PCDHB12	CERKL
WDTC1	SRPX	F7	BAI3
C2orf67	KLHL31	MTDH	VWCE
SLC19A1	PITX3	ZNF598	FAM167B
CA8	PAX6	KCNG2	PHACTR3
YTHDC2	SHQ1	CCDC90A	CCDC103
HEY1	IKZF5	NAT6	SFTPB
PSD4	ZFR	FBXL5	RIPK3
DBT	SNN	HS3ST3A1	TPPP
PPARGC1B	AUH	NUDT6	EXOSC1
PDXDC1	L1CAM	CHCHD8	USP25
PRRT3	HPS3	SLC35A4	OR51I1
TMEM178	ZNF479	FAM65A	RILP
TAF7L	PJA2	HIST1H2A	IL32
PLS1	AGTPBP1	C19orf53	CDC42EP3
P4HA2	OR2H2	SIX5	FLT4
SNAPC4	DYNC1LI1	MAVS	ARHGDIG
NDUFA3	KRTAP5-6	YWHAG	KIAA0319
ESCO1	NAT6	STK35	PION
TPCN2	CENPK	NUDT10	PCBD1
GNPAT	BCL9L	SLC25A26	ITPR2
KLHL13	PHF15	CDC14B	SLC22A10
RPS6KL1	FANCC	DGAT2L6	IQCC
RP11-574F2	ANKRD13	FAM122B	CAMKMT
ARMCX6	C7orf73	TRIM54	DARS
AGAP10	BCAR1	DENND5B	USP43
C6orf126	AK4	SAP30	RAP1GAP
SVOPL	MAFG	STMN1	ZNF25
C3orf30	MLEC	ARL6IP4	SYN3
TRPT1	HIST1H2B	YY2	C9orf16
RPS6KA5	TYMS	MT2A	RP3-486B10.1.1
RBM19	AKAP2	SIRT4	RP11-1396O13.9.1
PATE2	YEATS4	RBCK1	OR51B4
GOLIM4	HCN2	C22orf26	WDR65
MYH14	KCNG3	RAD1	OR2L13
CHADL	MLXIP	MAP4K5	RP11-1035H13.3.1
UCP3	ELFN1	ESPNL	CCDC113
TCEAL3	BHLHE40	RAB5A	DGCR6L
STXBP2	RNF2	IPMK	LAMA2
METTL17	SMARCD1	SNRPB	

LIX1	SETD1B	C22orf25
VWA5A	AFF3	MAG
TUBGCP2	ZSCAN22	PIP5K1A
SPATA22	VAMP3	AP3D1
GPR31	TIGD1L2	NUMBL
PHTF1	EMD	PCDHGC4
MTOR	ZNF664	PPT2
HNF1A	RBCK1	HPDL
SAT1	STT3B	PPAN
CUL9	HSD17B12	SPHK1
TM7SF2	STXBP5	MND1
GPD1	PDCD2	RAB35
MRPL55	ISL2	SLC10A3
WDR83	PRIMA1	CNTFR
KIAA0564	EMP3	HAUS2
HES4	FGFR1OP2	OXCT1
PABPN1L	EIF1B	POLR1E
HAVCR1	AC100808	SCRN1
NPFFR2	SIGLEC15	ZNF268
BLOC1S1	TKTL2	PRRG1
CELSR2	RRAS	NACC2
OR1E1	RCOR1	KCNG3
TUBA3D	STK17A	TRIM35
TUT1	KCNG2	NAT16
ZNF419	HRCT1	DCTN4
ZFYVE27	RRAGA	ARPC3
KCTD3	HPRT1	INSL3
CYP2D6	GNAL	ARHGAP29
KIAA1875	RCE1	TIMM23B
OPHN1	AMOTL2	IFITM3
ITLN2	CCDC36	PDZD8
PABPC1L	PGF	PAIP1
COG2	SGCE	MED22
RAVER2	SPCS1	KLHL7
RAB11FIP4	CPS1	ZNF599
REN	C3orf38	ZFP91
SLC43A1	FGF11	RHOC
APOO	CDC42EP2	CTSL1
PAIP2B	COQ5	TTC7B
WBP1	JRK	DSCC1
WNT11	ZNF787	SGOL2
C7orf25	RANBP1	HRCT1
FBXL15	CELSR3	HSBP1L1
CHMP4A	SSU72	ACTN4
FAF1	ZNF891	CTH
COQ9	GYS1	ZNF596
NDUFA13	ERI3	LHX5
AQP12A	B3GNT9	FAM204A
ZNF615	PHPT1	MYO1C
ECI1	UBE2M	MRPL3

OBP2A	MRPL13	FTSJD2
SETD6	SLC39A10	CLEC18C
PRDX6	ISCA2	NF2
RNF113A	TSPYL4	CCDC85C
ECH1	SUB1	SNX12
ZNF239	FAM205A	NPBWR1
TBCE	PTF1A	YIPF5
RP11-353J1	ZNF295	SMAGP
MARK3	TOMM5	C9orf172
INO80B	TTC9C	TFAM
AC131180.:	SEH1L	SNAPC2
SLC22A5	SMN1	LMAN2L
GREB1	EPS15L1	RP11-630A13.2.1
CYP8B1	WDR87	HNRNPA3
SAMM50	ZNF852	CDCA4
CTAGE5	CDH2	APOPT1
ANKRD39	NOLC1	TFR2
FIBP	BRIX1	TSPYL4
HADHB	TBCA	ARL10
FAM176A	TBC1D23	ASPDH
SSPO	UBE2H	C14orf43
ALDH18A1	EIF3B	TOMM20
KIAA1324L	ZIC2	HK1
SP140L	MEF2BNB	TAP1
RFX5	CDKN2AIF	GOT2
SF3B1	GARNL3	HIST1H1A
TBCC	RAD23B	SP9
TRPS1	RAB8B	TRUB2
ITSN2	CTNS	NECAP2
TTC12	GPR32	MEGF8
ZNF282	SP5	TRIM7
MYH6	MPP6	FGFBP3
SURF1	CD274	KIAA0020
C14orf102	FKBP1C	ITPRIP
ALDH7A1	PTPRG	WNK3
IFT122	FAM120A	SHQ1
OPRL1	MEMO1	TMBIM1
PPP1R12C	GRM2	DCAF16
HOXB8	MKRN2	FBXO47
MST1R	ANKLE2	TGFB1
LGALS3BP	KIF17	PLP2
STX18	STX2	CRAT
WDR33	BCL2L1	LRRC8A
SCHIP1	ZC3H15	GJC1
ACADL	MCM4	MTA2
HSP90B1	RBL1	CD300LD
USP16	BEGAIN	PTBP3
UTP20	CDRT1	MFRP
ZNF487P	MXRA8	WDR46
ITGB1BP2	MFRP	CD3EAP

PAN2	PAFAH1B2	TPX2
HSD17B7	ATL3	TPM3
ZMYM4	ZNF880	CNBP
RP11-1286I	RBM42	NDUFB4
SAGE1	GOLGA7	HSPB2.1
FAM71E1	SRRD	DSEL
TMEM234	SEC22A	TPBG
RDH8	MRPL32	ZNF716
POC1B-GAL	C5orf22	C9orf89
TTC3	UBAP2	SETD1B
CTPS2	C12orf52	ARL2
PPFIA4	RNF112	HIST2H2AA4
LSM10	FRMD8	HAPLN4
GCFC1	ACCN4	TONSL
RGPD3	SUSD3	UCMA
AC004381.6	PRDM10	SGTA
RAD50	MEGF8	PCDHA13
GLT25D2	NAB2	CAGE1
CAPN10	YY2	NKIRAS1
TAX1BP1	GRHPR	XRN2
OR3A3	MRPL43	ISM2
GTF2B	S100A11	PSG11
METTL3	ABL1	MAPRE1
C22orf40	TMEM55A	TOMM40
C16orf46	RP11-93K	ATP2B1
RMND1	TFAP4	C19orf57
B3GALT5	SRGAP1	FAM70B
ZNF19	PTPRB	DNAJC21
TMEM74B	EXOSC3	MTMR12
PTRH1	KHSRP	CIDEA
EPB41L5	MRPS36	AOC2
C2orf78	CAGE1	RBM48
GCNT2	SKA3	BAG3
BBS5	GTF2H2C	LRRC42
NOP58	MAP4K5	MIR3654
TEAD4	CITED2	FBXO43
DNA2	AKT3	C9orf5
STK38	CYTH2	RAB3D
PRPF39	GIPC3	TRIM28
MCOLN3	NFIX	PDE12
ZNF547	VAV2	FAM131A
ATP6V0E2	ELK3	SKA1
CCBP2	ERGIC2	ANP32B
FAM192A	TMEFF1	C12orf24
DSCR6	AHSG	LDHC
ZNF704	GPR150	PHF19
SCAPER	PTPLB	AGTPBP1
LYRM2	RGP1	SERINC3
ZNF473	RABEPK	FAM57A
SGSM2	RBM48	PIGZ

RBM33	ANAPC10	C5orf62
USP54	BRD4	BFAR
SLC30A10	CD59	CUL2
DEFB107B	RAB43	SRSF1
ZNF638	CIZ1	KIF17
CEP57L1	NCKAP1	TMEM186
CEBPZ	TFPI2	ATE1
MTA3	EFEMP1	GRHPR
CHRNA7	MND1	AP3S1
CHRNA7	PPP4R2	VTI1B
ANKRD53	SS18L2	VRK3
KIF22	SSH1	HOMER1
RNF151	TMEM239	VGLL2
NSUN7	CERS1	AC011498.1
OGDHL	SYNPO2L	SMARCB1
WIPF3	AGBL3	C6orf57
RBM6	IGDCC4	ROMO1
UTF1	SIK2	GATAD2A
RTN4IP1	EDIL3	CENPK
EPCAM	ZCWPW1	TMPRSS9
ARMCX4	AIG1	CSRNP2
KIAA1383	FADS2	TATDN2
MAP7D2	FUCA2	PDCD6
NUB1	MTFMT	SYNPO2L
LONP2	RP11-849I	KLHDC10
OR11H12.1	C3orf78	BTBD2
OGFR	CHRAC1	OTP
RS1	PRR24	PSMF1
GATM	CYB5B	PTGR1
DAPK1	ERBB2IP	NAA50
SRSF6	TRUB1	FBL
ANKRD36B	TMEM209	SRRD
RPAP2	DNM1	RPE
RP11-1286I	CES2	CSTF3
GOLGA4	HERPUD2	TFE3
CDK5RAP3	HIST1H2B	CRK
SIN3B	ADAM11	PROCR
RSAD2	NSUN3	NSMCE2
GBA	TRIM54	ZNF408
TTC21B	BASP1	TIGD6
DDX58	YKT6	VDAC2
CYB5D2	GJA3	NAA15
PGBD4	SLC25A1	LRRC58
COBL	PLEKHA4	SDHB
SNX14	MRPL11	TXLNA
EIF2AK1	ATP13A3	TMEM69
TEC	SLC5A10	PPP1R13L
DSCR4	HINT1	C9orf41
CHAF1B	ZNF623	ZC3H4
CATSPER2	HSF2BP	CD163L1

FAM96B	MSN	ZNF395
SRGAP3	RNF4	OSBP
ZNF845	ADM	TCEB1
RGL3	ZCCHC9	SPATA5
STK36	PTGER1	METTL15
KRI1	LCNL1	C11orf85
FARP2	STEAP3	KLHL21
SLIT1	CAMK2N1	SEC14L2
ELMOD1	GINS3	C6orf195
MRPL46.1	EIF2S3	FBXO45
ZNF761	DCDC2C	INPP5F
PRR15	TPBG	C5orf34
ZNF559	IGFBP1	SLC38A1
PLCB1	ZCWPW2	DAZAP1
ZNF709	COL11A2	RP11-293I14.2.1
MTHFS	TMEM11	TMEM200B
FAM164C	HERC4	LGALS1
TTC39B	GAS2L1	UBA2
SRA1	GLO1	CAPRIN2
CCZ1B	ZMYND19	ZNF622
MTERFD3	C13orf16	PGRMC2
NCKAP5L	TNFRSF10	NT5DC2
DNASE1	PLK1	NR1D2
EGFLAM	TAF10	GSPT1
TMEM184A	ROBO4	OGFOD2
GAB4	PRICKLE3	ZFAND2A
SLC4A10	TFE3	PLXNA1
PIGL	POLR2E	RASGEF1C
SLC5A11	TOR1A	ASAP1
CHPF2	TBC1D2	FBXL18
FAAH2	MAGOHB	UBE2E2
CEP164	ZBTB46	ISYNA1
GNAS	ASPH	FAM127B
PHKA2	NDUFAF3	IKBIP
C10orf32	TRIM71	GPER
NAP1L2	MTMR12	CTDSPL
PRPF38B	PACRGL	C11orf57
CBX8	HMMR	IL1RAP
KRT20	SLC31A2	IL1RAP
GMEB2	C9orf172	CBY3
CEBPA	NR1D1	MEMO1
GOLGA5	LYRM4	FUCA2
ASGR1	MPP5	BTBD19
RANBP10	C11orf71	PSIP1
MIA	THOC6	TBC1D23
WRAP73	SWI5	C4orf46
ATN1	KCTD7	EBF3
ACSS2	CREB5	PLEKHG2
DCDC2	EEA1	OLFML2A
TFPT	CDV3	CNTNAP1

CCDC64	ZDHHC5	KLHDC8B
KLHDC7A	GINS4	TANK
PLCE1	IPO11	CUL4B
C19orf63	NDUFS6	SEC61A2
ACBD7	ELOVL2	TMEM151B
IFT52	BICD1	ODF3L2
SLC26A8	KNTC1	C19orf55
ZNF679	AKT1S1	OR2A14
CST11	TMEM39A	FCHO1
RTP4	NUMBL	GDF1
ZMYND11	C13orf27	CLTA
E2F7	ASF1B	C2orf89
SNX29	SLC25A32	IFITM2
NADSYN1	C3orf49	MBTPS2
CCDC148	FLG	ZNF570
ALAD	DHX33	TAF9
ZNF774	MED30	PRRC1
SLC4A9	AKAP5	PNLIP
NARG2	ALDOA	CAST
DYRK3	UBE2U	RNF114
CTNNBL1	URM1	MAGOH
ZNF433	CERCAM	FAM134A
FBXO42	DTD1	MAPK12
DDX24	CD3EAP	EMD
ILVBL	TMEM186	SLC4A1
ZNF783	FAM20C	PPP2R5B
NBEAL2	C16orf11	MPP6
KIF4B	THY1	TYMS
AGXT	CNTNAP1	SRGAP1
TRMT1L	KLK1	KXD1
SLC25A14	CELF4	VAV2
PMS1	OPA3	C12orf65
ACP6	GLS	CDH2
ANKRD45	ATXN1L	NT5E
TUBA3C	SEC23A	PHPT1
POLR1C	HIF1AN	POLR2H
PILRB	GPER	EIF6
HNF4G	MED9	MNT
NXF5	C16orf61	AC100775.1
C14orf159	ZCCHC2	TFPI2
NUDT22	CA5B	CAPNS1
WDR5B	EME2	RABL3
C1orf61	ABHD6	TMEM240
SYTL3	PPP6R1	EPM2AIP1
FAM213A	LDHA	MATN4
IDH1	UBE2D2	MYO10
FBXO11	MTA2	METTL6
NOP2	TRNT1	UBE2Z
CEP70	NXT1	ALLC
BCL2L14	NANS	FAM120AOS

GPHA2	NACC2	HSCB
UBC	MAPRE1	RBM12
OPLAH	TSR2	ERI3
MCAM	ALLC	SLITRK1
PACSIN1	RNF5	PANK2
OR52B6	C19orf55	ANKRD30B
ZNF439	TMEM132	CDCA8
ANKRD35	MYL9	AURKAIP1
MAN2C1	TUBA4A	PC
CSAD	SP7	CCDC73
CEP104	IGF2BP3	SDCBP
SETMAR	DHFRL1	FAM9A
TXNRD1	ZIC5	HAS2
RIPK2	GBE1	TIMM44
LRP6	AP3B1	QRSL1
RBM39	MRPS35	RAP1B
EML1	SLC35F2	CDCA7
ACTN2	GAPDHS	NSUN2
FBP2	NKIRAS1	BCCIP
FAM162A	PSMB2	HP1BP3
CHD8	SLC2A1	PCMT1
DOCK1	ASB14	C1orf216
ACAD11	ZFC3H1	PDRG1
CEP85	XKR9	ARF6
SRPK2	TMED7-TI	DAP
ZNF613	BDP1	SEC24C
TCN2	TMX1	LARP1
PSMA5	ULBP2	EAF1
ATP6V1H	KLF6	NDUFC2
KIAA0895L	HNRNPAB	GFRA1
ALDH16A1	FAM196A	TPD52L2
SPATA2L	CMC1	VPS26A
CLCN4	GNA11	ACN9
PDE6A	ZC3HAV1	C6orf120
ALDH4A1	CRYAB	ACCN2
CGN	THAP2	CLSPN
LTA4H	RP5-972B	GRINA
UPK1A	IL1RL2	ELOVL3
RNF213	NRSN2	SERF1A
TIGD4	TADA3	ZNF623
ATG4B	HSD17B14	SPHAR
ZDHHC23	UBA3	CDK7
C1orf56	BRAP	UPK3B
FASTKD1	10-Sep	C3orf14
BCAP31	C6orf48	RP11-849H4.2.1
AGPHD1	AP2S1	DDX51
HPR	RPSAP58	RASA1
RHPN2	CAMLG	TUFM
MBD6	POLE3	LSM4
ENPP3	NF2	MAEA

KIAA1614	FHL2	SHC1
ABCA5	ACN9	PTRF
CPB1	PTPN12	C3orf37
HEXDC	THOC5	TCP11
ACOX3	C14orf109	GRK1
ZNF681	STMN3	CD274
FIG4	SGK196.1	FEZF2
ABHD11	PAICS	CTU1
TMEM111	TMEM184	ZNF121
REPS1	SERF1A	NOP14
GTPBP4	GOLT1B	RP11-111K18.1.1
SPNS1	HIST1H3J	NRIP3
LTBP1	SYS1-DBN	ARL6IP6
FN3K	CLPTM1	CHML
PCK1	HIGD1A	EIF4H
QPRT	STAM	BAG6
POTEI	BCAS4	NOV
RGL2	SLK	CRKL
RAB17	ELAVL1	C11orf48
PARP6	IRGM	NCLN
ZNF160	STK32C	THAP2
HEXA	RFPL1	NFIL3
C1orf210	F2R	UTP15
NDUFB8	HIST2H3C	TIMM50
CD40	TNFRSF12	ARPC4-TTLL3
RP11-977G	BBS10	MRPL21
PSMD4	USP53	RP11-585F1.10.1
DDX60	ATF6B	IPO7
RBM45	FAM120C	ATAD3A
DCAF8	FBL	FPGS
TMEM9	RAD18	SLC23A3
GATC	ZC3H12B	SIGMAR1
SPTBN5	SLMO1	OTUD6B
HYLS1	PIAS2	TMEM14B
C20orf118	MYO1C	RAB11FIP5
ARTN	GNE	RP11-122A3.2.1
01-Mar	TCP11	ITGA5
REV1	SYDE1	SSH1
WDR34	FAM40B	DNAJC19
ESRRG	KIAA0664	BCKDHB
ZNF10	OXSR1	CNPPD1
C21orf58	WDFY1	GALNT9
NCALD	ZNF660	BCL9L
CUL7	KIAA1967	IL11
PCDH15	FSCN3	AVPI1
TBX19	LRRC61	ANO4
ITGA7	GPX8	AC091153.1
KCNIP4	DUS4L	EXOSC6
ANAPC2	RAB2A	BSG
IFNAR1	RPSA	PPID

AP1M2	MPHOSPH	WDR82
BRD7	FAM72A	ADAMTS6
TIMM17B	PRDM4	SSFA2
C7orf59	MUL1	PDCD5
ZNF350	ITGA5	AP3B1
NLRP8	RP11-3N2	PCDHB14
SLC15A1	ENY2	NTN3
ZSCAN20	QTRTD1	PPIA
TMED3	PRIM1	INCA1
TMEM164	UCHL1	FGF2
USP17L5	KIAA1191	SDC2
ZNF444	C6orf195	NR2C2
TDRD5	EIF2A	NOL7
DNTTIP2	PSMC4	TNFRSF12A
ZNF416	PRSS33	FAM102A
AHSA2	ZNF598	ACTL6A
MYL5	LIN7C	GPR176
ARMCX1	C5orf35	CRTAP
RBM5	PVR	PLCD3
PYCR2	CTSL1	TBC1D19
MTIF2	AFF4	SAE1
ZNF443	SYT14	WASF1
HEATR5B	PA2G4	HK2
LDHB	ZNF654	AC026271.6.1
C2orf56	NDUFB4	SCML1
NOP56	UQCRB	FAM180A
PCSK7	RAD21L1	C10orf122
EDEM2	ZNF141	BATF2
SAG	GLE1	USP22
KPTN	HP1BP3	SRPK1
GDF3	SMARCC1	UBE2F
UTP14A	RALB	RBBP9
TNRC18	FAM120A	BCL2L13
ALMS1	GAPDH	FXR2
PRPF4	CPNE8	NTAN1
PRKCD	NAA30	POLR2J2
USP48	BACE1	MRPL30
ZNF799	ETS1	PBX3
MAB21L3	ZNF724P	TMEM110
ZNF544	PCDHGB4	MUTED
DDX39A	TBCB	MAPKAPK5
HOOK2	POM121	B3GNT4
CPPED1	VPRBP	PGF
POLR2C	CDC20	STT3B
C6orf170	C1orf187	PEX2
GTF2IRD2	NLGN1	MRPL22
ZNF773	ARL2	UROS
LRRC45	SPTY2D1	ATG13
UNC45A	FAM101B	TAF9B
UNC45A	SLC25A24	OSGIN2

GOLGA6L9	ATG10	LSM3
ZNF57	RAX	ORC2
EML2	C7orf42	PRDM12
ERGIC3	GRINA	USP14
POP5	REEP3	THRSP
AIF1L	PDCD10	PRDM10
KIAA1737	HBB	TKTL2
TTC30A	TULP1	STK25
C2orf82	POLR1E	NANP
SORL1	UCMA	RNF130
CLDN25	C14orf178	SMS
OR52B2	KDELR2	SIRT5
OR2C3	DSCC1	ISY1
GAD2	ETHE1	TARS
CLCN2	NSMCE2	ADAMTSL5
ZFP90	STK39	SMARCD1
CEP250	IGF2BP2	NOL9
RP11-1286I	FXN	ADNP
SCCPDH	ZNF580	STK39
MYO7A	FAM164A	CTNS
THOC2	XRRA1	PCGF6
EXTL2	NPM1	PTPRB
SLC35E3	QRSL1	PLEKHA4
ASL	DIXDC1	ELAVL1
SCRN2	CSPG5	TBCB
C20orf43	SNAPC3	NAA20
LRRC37A2	C14orf39	UBXN7
ATP6V1C2	C6orf120	ACTN3
CEP290	STARD6	UNC119B
GATAD1	STC1	AGBL3
GGA2	PSMB6	KDM2A
PCDHB5	SLC37A2	RIF1
MED23	C5orf15	C1orf51
ECI2	TIGD6	SHARPIN
WDR52	RNF212	CREB3
SNX25	NOP14	ZHX1-C8ORF76
SENP6	LINC0049	UBA3
PARD3B	OR2A14	SLC5A3
UBAC1	NAA50	C10orf47
DSCR8	RAB6A	CDK4
PGBD2	CECR6	MOCS3
NDUFA10	SLC27A4	PLCB3
HOGA1	LMNA	GLO1
GTF2H4	BTBD11	ATXN2L
NDUFV1	KCNK3	PPAT
TCEANC2	AQP11	KIAA1826
COL9A3	SQSTM1	RFC5
HIPK2	DNAJC19	PRR3
ZNF541	GNAT1	ACTR10
ZNF607	C22orf25	GLIPR1

RHPN1	INVS	ST5
GTPBP6	MAT2A	GTF3C4
RIMKLA	NOV	PTPLB
PTCD3	TMEM39E	GNA12
TNRC6A	EEF1E1	QRFPR
TMCO4	RPH3A	SOX15
AGK	DMWD	C5orf65
UNC13A	C11orf65	FAM40B
ANKRD20A:	CCDC58	MRPL2
DSTYK	PTPN23	CAP2
IRF2BP1	CPOX	USP42
NUMA1	RPL27	IL5
MAST2	ZNF501	FAM20C
PRMT7	PURA	EIF4G1
IFT80	SFR1	GPR172A
SUV420H1	RBM3	CLOCK
CSRNP3	ATF7	FYTTD1
HHAT	PNO1	PRPSAP2
MX2	COTL1	MIR4720
RABEP2	PRRX2	ZNF805
GRHL2	PIN4	SLC16A1
CNOT1	TCTA	PTPMT1
SUPT7L	PYGL	HNRNPUL2
PNPT1	MYOM3	NRBP1
AMN1	MYBBP1A	C11orf30
RP5-1180C:	NUTF2	RPLP0
LGALS14	NPC1	RNF10
C10orf11	PCDHB12	G3BP1
ANKRD36	KCNK13	SDCCAG3
DIP2C	PSG11	ACTN1
PRSS50	GSG2	BRD4
ANKRD63	GSK3A	RAB1B
ASTE1	WNT7A	GJA3
GPR89B	CASC3	TMEM44
C17orf56	CFL2	STAT1
ZNF516	FCHO1	SIK2
RPN2	EXOSC6	NR1D1
SAMD9L	AC013461	SIGLEC15
PARP2	CYB5R2	SF3A2
CACNG7	VTI1B	KDM4D
HLCS	NR2C2AP	IRGM
ZDHHC12	GXYLT2	ERCC1
RDH13	CCDC71	NRF1
UBB	SURF4	METTL5
ZNF701	PWWP2A	C17orf74
TRMT5	HIST2H2B	ARMC6
WIBG	MRPL3	GNG12
NOMO3	GPR173	PFN1
MSH2	MRPL22	TOMM34
CIAPIN1	RASA1	RRAS

HOXB6	SLC2A3	NOXRED1
KLK8	BAG3	HIST1H3D
ZNF627	SPRN	TMEM38B
TIA1	ASPDH	CAD
DMTF1	KIAA0947	ENO2
BUD13	ACVR1	SRMS
GDAP1L1	ACYP1	PITX3
PCDHA2	IFRD2	NR2C2AP
INHBE	FAM49B	SETD1A
RBM25	SPC25	ROBO3
EGR4	PTGR1	RNF112
ATP6V1G2-	FAM204A	CCDC124
SAFB2	RPL37	QSER1
ODZ1	CCDC90A	ZMYND19
GABRR1	DPYSL3	CTNNAL1
FAM103A1	CBY3	RABEPK
ZNF253	NME2	TUBA1B
LPPR3.1	PTP4A2	FLNA
TAF6L	TMED7	WFDC8
ART5	SRSF1	C5orf43
HAUS8	FYTTD1	TIGD1
CCT7	TMPRSS9	KIAA0907
RP11-6F2.7	PTAR1	TMEM88B
DDX28	ASPHD2	PRPS1
SRSF7	OXCT1	CENPA
NACA	CENPW	CITED2
TRMT11	HNRNPC	TRIM56
RANBP2	PPP1CC	NETO2
MBD4	SP3	ZNF292
TAPBPL	MIS18A	C7orf11
ACIN1	F3	ELOVL4
CREB3L4	CCK	NDUFA12
SMG7	TEX264	TCTE3
PRRG2	CAPRIN2	NPC1
CCT6B	ZNF716	WASL
VEPH1	EIF4A1	C4orf43
TAS2R31	PINK1	TSEN34
UGP2	SERTAD2	FBXO41
CEP350	BCL7B	TWIST2
MYPN	RBFOX2	MFAP5
PPP6R2	KCTD10	C9orf21
EHMT1	HNRNPR	RFX1
ZNF17	SRP72	WDR43
C6orf127	LETMD1	PAOX
C19orf77	RPL35	ZNF585A
TH1L	CHURC1	CACNB4
IL17RB	RPP30	ULBP2
PTPN18	BFAR	POLE4
CLN6	NFIL3	NLRP9
TLN2	MAP7D3	MRPL47

TLN2	GZMK	ZFP64
TOP2B	FGFBP3	ZNF259
CCDC138	RP11-196(	NPTX2
JMJD7-PLA2	YWHAZ	SPIN1
OR56A3	TAMM41	AP1M1
SPZ1	RAB28	RPS3
ERBB4	RPS18	SRD5A1
DIP2A	TBC1D22E	GFM1
AMIGO1	RRP1B	NIT2
DAGLA	FAM72B	ZNF140
ZNF614	COL4A3BI	NRBP2
ATP6V0B	ELOVL4	NLN
HARS	C5orf65	LSM6
TCEAL4	NDST1	RPUSD3
NDUFA9	NDST1	SEC22C
CHORDC1	COPZ2	ABL1
SH3GL3	TP53TG5	FTH1
CHRM5	PROS1	DNAJC9
PHF20	ZNF334	CMTM6
DRG2	LASP1	RASAL2
RP11-357H	ARL6IP4	GLIS3
FAM169A	AP1M1	AP002884.2.1
ZNF513	NDUFC2	CTC-260F20.3.1
FAHD2A	CCDC9	ZNF85
GABPB2	ERH	SERPINH1
FBN3	KCTD9	KIAA1191
XPNPEP1	CCDC15	STK11
MAP3K7	XIRP1	RP11-87C12.2.1
CLDN10	LSM4	RFWD3
TJAP1	ANKRD33	TROAP
TFRC	PTP4A1	ATP5G3
CASP7	POLR2H	MTG1
GOLGB1	CKS1B	RP5-972B16.2.1
KLHDC9	RPS3	GPX5
HACE1	C8orf44	U2AF1
ZNF556	SH3BGRL3	UBE2D2
RAB3GAP1	SPPL3.1	XKR3
MX1	PCIF1	ZC3H8
DNAJB8	KCNV2	HMMR
CDK5RAP1	NSFL1C	CREB5
XPO1	PRDM12	DEFB118
AMPD2	NXPH2	SPATA21
TBC1D1	POLD2	TCOF1
ANXA4	ISCA1	PHF7
ZNF552	FAM133A	GLUD1
ZCCHC17	SLC35A2	ARL9
GTF2IRD2B	HIGD2A	TMOD4
ANKMY2	TM2D2	STRN4
MMEL1	C22orf39	CBWD3
AHCTF1	HYAL2	NANS

NEBL	STYX	NRSN2
ASTN2	TMEM167	GLE1
ODF2L	SYNGAP1	THOC6
HDHD3	SFXN5	SCAF4
ADAL	ZRANB3	CRBN
MAP3K11	TXNL1	YARS2
STAP2	F8	SENP1
ZGPAT	PRR3	FAM64A
DCLRE1A	C9orf82	NLGN1
ZHX3	NTSR1	TATDN1
GRB14	SLC35B2	DTD1
ZNF200	KDM4D	FGFR1
APC	PPP1R15A	LIN52
FMO6P	C2orf89	LMNA
NOMO2	MELK	PRDM13
TTC7A	SNAPC1	YWHAE
TAS1R1	SPRYD4	DPF1
TBC1D8	FADS1	C11orf65
HOXB5	05-Mar	GTPBP8
CREG1	TP53RK	PEX26
CLPB	CDYL2	PNO1
ZNF766	CA12	KLF12
FAM174B	SMNDC1	ZNF653
SP100	UTP15	PYCRL
PLEKHG5	UNG	KCNV2
NMB	DAP	ZFYVE20
FAM120B	BRD2	CHN1
CEP95	FAM211A	KLF6
PPDPF	OR1Q1	HIST2H2BE
SRSF5	PSMB9	TMEM184C
FAM161B	HDGFL1	DMWD
TCP1	SMTNL1	MUL1
DTX3	LDHC	GFPT2
IQCB1	GTF2H2	CPNE1
WDR91	RAN	RPL8
IFI27	GNB1	UBE2H
PIKFYVE	BOD1	KCNJ14
RP11-1286I	LRFN1	AUH
HIRIP3	PCMT1	CELF4
RNF183	PEX13	NDUFB6
POTEH	MB21D2	HNRNPUL1
SCNM1	GINS1	ISCA2
MAGEB6	ACTN1	HERC4
SHF	MTX2	XYLB
PLA2G4D	RPL36	DOM3Z
AKAP17A	STMN1	MFI2
C22orf42	ADO	FIP1L1
C20orf111	SLC2A10	WDR4
PDCD11	AP2M1	FAM199X
BEND5	TMEM194	P2RX3

PRDM15	KXD1	HIVEP2
RRP1	EOMES	MED29
UBE2CBP	THRSP	RMI1
ARFRP1	GLP2R	MED15
RRN3	YBX1	CD44
IFIH1	PRPF40A	HIST2H2BF
PRADC1	MRPL47	TOR1A
ELP2	PCDHGC4	DALRD3
ARFGEF2	AC008073	CENPW
ANKZF1	PPP3CB	ZNF526
C1orf27	ABCA1	CEP41
PLA2G2C	GMPS	C10orf88
INPPL1	PKN3	PDCD10
ZNF432	SEC22C	C3orf49
DDX27	SPRY4	CHRDL1
CNTN5	NOXO1	C12orf76
C2orf72	ABHD5	LRRN4CL
CCNI2	FHL3	PAWR
RGPD6	CDK17	P2RX6
TOP3B	TYW3	SLC2A3
CBWD2	SAP18	ELK1
RP11-632K	ZNF736	SQSTM1
PARP9	RP11-728	CA9
GTF3C1	SLC35E4	PRKAA1
MTR	PSG5	HSPA14
DNASE2	MPO	VMA21
TNFRSF25	NUDT21	NOA1
PFKFB1	MAPK11	PRRT2
APLF	PORCN	DDX55
C17orf61	RP11-286	TMEM11
NVL	RPRD1A	SLC9A3
LAS1L	PPP1R13L	NDUFB9
RNF8	DENND1A	SATB2
PAGE4	SDC2	CHKA
DNAJC17	CAPZA2	SPANXN1
NKAP	DSCR3	AC078802.1
C15orf38-A	C10orf2	FNTA
CLK1	BEST2	KIRREL
CLMN	NLN	SPRY4
HENMT1	HIST4H4	TMEM201
C10orf28	TRAPPC2F	DBNDD2
PPIP5K1	TOMM40	KPNA1
PRPF40B	PPP6C	FRMD5
CUL3	SUGP1	PLAUR
CDC40	CEP72	COPZ1
AGBL5	PSMC3IP	KIF20A
IVD	ARIH2	ZNF551
H2AFJ	MMP23B	PRKCSH
ARMCX5	KCNG1	CALM3
OR52E6	GPS2	NCOA5

ERI2	CELA2B	FAM45A
CCDC77	PPFIBP1	ZBTB46
TP53I3	RAB3B	KCNE2
TPR	MRPL30	DNAJC18
ZNF335	C5orf30	PRRC2A
CLPS	ZBTB7A	RNASEH2A
AFMID	PDCD6	CRCP
GPT2	CDCA4	AKAP5
PEX5L	ODF3L2	POT1
UBA1	GPC6	FBXO34
CHD3	DUSP7	FBXO34
TFCP2L1	NT5DC2	43350
WDR59	CLEC11A	ZW10
ZDHHC24	CTNNAL1	SLK
TTLL1	SGMS1	C12orf23
LRRC26	C20orf24	IL17RE
HESX1	FAM65A	CIRH1A
NPEPL1	KLHL21	SLC35E4
DUS1L	UBA2	HNRNPF
SAMD3	EPHA6	CAPRIN1
OPRM1	KANK2	ZDHHC8
PRPF6	HIVEP2	SSU72
CCDC93	AL928654	PHF23
PRKRIP1	C5orf24	MSN
GOLGA6D	IRAK1	RBL1
TMEM132A	WDR82	ZNF501
MAGI1	CYCS	STARD6
AKAP3	ZCCHC7	SLC2A11
COMMD4	FRMPD4	AFAP1
TUBA3E	SACS	HOMER3
SUOX	TAOK3	WWC2
EIF3D	FANCB	CD59
AADACL4	04-Mar	UBE2R2
NEMF	C2orf16	URM1
IGHMBP2	SNX5	NES
ZIC1	CMTM7	ZNF296
SEPHS2	TMEM151	ZER1
PSG8	FLNA	PTTG1
AKAP9	CD109	GADD45GIP1
TRPM2	SIRT4	SGCB
HOXB2	MUTED	FARSA
BRWD1	FNDC8	USP17L4
MR1	MAFA	S100A10
SPDYC	RPL38	STX2
NUDT16	ROMO1	HIGD1A
C1orf9	PIP4K2A	ZNF555
ZNF589	SLC9A8	FAM98C
MAGED2	ZNF98	TIMM22
USP24	TEAD1	CYB5R2
TRIM11	C3orf67	CHD5

SLC10A5	COX6C	POLR3A
FGD4	CHEK2	BCL2L2
DNAJC27	MAD2L1	PDCD2
UBR3	P2RX3	MPL
DCAF6	ZNF114	ACOT8
PNPLA3	HIST2H3A	SENP2
LRIF1	GRWD1	C8orf85
LYG2	INCA1	DHRS4
ZNF808	POLK	TKT
LY75	CENPQ	HNRNPL
AMACR	MXRA7	UQCC
FKBP3	MED15	GPR32
DYRK4	ASNSD1	WIZ
C16orf80	METTL6	FOSL2
KIAA1147	NDUFB5	NSA2
C21orf33	C5orf28	LARP1B
SLC24A6	PDCL	ZNF829
KRT8	ARPC5L	NICN1
MED8	C19orf29	TFDP1
TARS2	CEP55	BTBD11
EDC4	SHMT1	HSPA4L
STOX2	MRPS6	TMEFF2
SLC27A6	RPS11	PDS5B
C1orf51	FCRLB	EPN2
KIAA1731	FOXK2	CASP5
PFAS	ORC5	C16orf11
WI2-2994D	ELMOD2	ASNSD1
MYBL2	TOP3A	DNAJC25
TFAP2C	FAM116A	PPFIBP1
MIF4GD	HYAL3	SMG6
SCAND1	CKAP4	PCOLCE
RFWD2	CCDC90B	CDKN2C
HADHA	SENP2	STXBP5
NGRN	CHCHD8	FBXO33
COPB2	BCAT1	BCAP29
ARFGAP1	UGCG	VWDE
RASGEF1B	ZNF829	FBXO22
LCMT2	CHAF1A	PITPNM1
DYNC1H1	SPHAR	HCN2
ILDR1	AP005814	TWISTNB
DOCK10	C9orf80	USP11
PPME1	CHD5	GAS2L1
FLVCR1	CNPPD1	GAS2L3
STK11IP	AC026271	FADS1
MCCC1	NCOR2	SF3B5
BCLAF1	TIMM23B	XAGE1A
GUCY2F	CREB3	RECQL4
PGS1	ASB1	ACY1
ABCF3	ZNF460	EIF1B
DIDO1	CDKN2C	KEAP1

BRF1	CNOT3	TRIM74
ESPN	FAM168B	POM121
ZNF616	CRTC2	TPD52L1
MASP1	NDUFA11	ASAP2
ERC2	CCDC85C	DTX3L
RSPH3	VCPIP1	PIN4
GANC	TMEM44	FTL
DCAF4L2	PPP1R27	PIGO
PIK3R3	VKORC1L1	RRAS2
ACAT2	SLMO2	ZNF699
SLC17A8	SLC25A43	SMN2
BRWD3	CCDC63	ZDHHC14
FRYL	PITHD1	KIAA0226
CUL1	CCDC86	LRRC8E
ZNF512B	CTRB2	NRM
MAU2	ZC3H4	KNTC1
GFOD2	KLRD1	RASSF8
NFXL1	DOM3Z	SLC38A5
SASS6	TRPV3	SEZ6L2
P4HB	GLUD1	NDOR1
ATP9A	C11orf57	USP7
GGT6	PQLC1	CDV3
MRPL35	ISYNA1	EIF2B1
SSX7	APOOL	KIF18A
IKBKAP	REXO2	COBRA1
C14orf80	EGFL8	OR2H2
ZNF138	HNRNPL	ACYP1
PIRT	RRS1	ENO1
VPS54	LYPD5	NUTF2
PIGC	TOMM20	DNAJC8
NT5C1B-RD	DNAJC1	MAP1B
RFPL4A	SMC1B	CCBE1
ZNF530	TAPBP	GART
MORF4L2	SENP1	C19orf33
HIBCH	NPTX2	FAM210A
PPP1R9A	C19orf38	HDGFL1
RBFOX3	COBRA1	PDE1C
PUM2	MRPL2	DNAAF2
WDR27	ESPNL	CDKN2B
TMEM141	LMAN1	SPDYE4
ACADS	C10orf88	ETV6
GLUD2	C5orf39	XKR9
RP11-1286I	MAX	PTPRG
TMEM160	GATAD2A	PRIMA1
TAS2R19	PIAS4	SLMO2
DHX38	RAB7A	PRX
CHDH	QRFPR	DNAJA3
C11orf93	PCDHGB6	KLHL24
DLD	DBNDD2	PSMB9
RBBP7	PITPNB	DPH2

HAL	C3orf23	TIGD7
MFSD3	HK1	C14orf135
BIN1	SLC38A1	CUL4A
PDIA6	NUP43	AIG1
CCR8	WDR46	ROBO4
TTC27	CRY1	SLC16A8
ZNF304	METTL15	CADM3
SERPINB6	RAB10	NUP153
RPS6KB2	ARPC2	RP11-526L8.1.1
C12orf56	TMEM126	C11orf46
ZMYM3	WWTR1	MXRA8
G6PD	PAPD7	LZTFL1
LANCL2	CHM	CNOT4
SHCBP1L	RHOC	HAUS5
LPGAT1	CXorf56	SPC25
CXorf40A	SF3B2	SNN
SPATA12	ZNF345	DCDC2C
PPM1H	FAM199X	APEX2
WDR61	CTDNEP1	HNRNPAB
WDR61	FBXL18	BAX
YWHAB	NUDT2	AKNAD1
ANKS1B	ZBTB11	EMP3
TMPRSS3	IRX4	NTSR1
RP11-1021I	QKI	POP7
ZNF75A	SUV39H2	ALG10B
FMO3	PLAC8L1	SLC35B4
LRRC14B	IFITM5	RPL37
CDCA7L	FAM134A	CLIP1
LRRC37A	CDS2	FAM175B
ZNF256	IL17RA	INS
FAM136A	SOX15	CHEK1
ZNF562	SGTA	SLC37A2
BRAT1	ZNF99	NFIX
MNAT1	BCKDHB	ACD
NT5DC1	UBXN8	KCNIP3
EFNA4	FSTL1	C7orf60
LGI1	PRDM13	RSPO4
GOLGA8B	RPS25	PPIF
ZNF189	MAEA	ANKRD13C
C22orf46	USP42	CLMP
PFKL	ISM2	DHX30
ZNF620	NAA35	CKAP2L
PRAMEF6	PTDSS1	PRR11
C15orf62	BCL2L2	PPP1R2
IGSF5	COX7A2L	BCKDK
SLC25A12	SEC24C	IKZF5
FAM151A	DUSP1	HIST1H2BN
TNP2	HIST1H4J	RPRML
THNSL2	CBWD3	EFEMP2
PSAP	PCDHB14	TMEM132B

COLQ	HIST2H2B	GRWD1
TTC31	MPL	ADSS
C6orf226	KLC2	RPF2
NDUFB2	HCN1	CDC42EP2
PNP	MME	GNG10
AC010336.1	RPL26	FLG
L3MBTL2	ATG9A	MEPCE
SUV420H2	ITGB3	TUBB3
ZNF534	ADSS	RANBP1
SLC29A2	SPATA5	C5orf60
MYL6B	SPDYE4	RASSF1
FAM90A1	NAMPT	TTC23L
ATP12A	PSMD6	FHL3
RPS6KC1	C17orf81	KIAA1644
C17orf90	RPS10	C19orf38
SSB	RPRML	C18orf26
TFF2	TAF1A	LCNL1
LGALS8	PAOX	PTBP1
ECEL1	AP1S2	SLC16A3
PFKP	SIRT1	KHDC1
DNASE1L1	UBA52	MED30
DIS3L	KIAA0020	C8orf44
COPG	HNRNPA3	CGREF1
SLC38A7	KIRREL2	NCOR2
TRMU	IL1RAP	FOXK2
OR2W3	IL1RAP	PML
RBM17	PES1	CSE1L
PPP2R5C	KRBA2	FAM120A
PROM1	NT5E	FAM92A1
ACTR3B	SCARF2	KLF17
CREG2	SHB	RAB7A
CDH26	OLFML3	LETMD1
ALDH9A1	GALNT2	MRPL36
AC026150.9	PCDHGA6	GANAB
GTF3C3	GPR153	ZNF490
ZBTB37	PCDHGA1	TMED1
ACTR8	HIST2H2A	FAM133A
MGAT5	PHC3	LACTB
SHISA9	ERI1	KCTD5
EEPD1	TIMM8A	DUSP15
MOGAT3	TSC22D2	RBMX
SUN1	SIGLEC11	BCAS4
SLC25A13	MATN4	CCDC36
LRP2	GPX5	YAP1
LRP5	FNDC3B	PPPDE1
ITFG3	P2RX6	NDEL1
KCNT1	PTBP1	PWP1
ATG16L1	DEFB118	SP3
KIAA0753	MAN1B1	NGLY1
HDAC6	MAD2L2	HUS1B

KATNA1	CTF1	TMEM14C
ADAMTS19	RPL15	INTS6
PNKD	WDR55	HIRA
KRT83	SGOL2	EPC2
DDIT3	ARHGAP3	MAPK1
MC4R	C8orf59	PSMB6
CLK2	FAM206A	HMGA2
MKRN3	TFR2	YJEFN3
CDH8	C9orf69	TNPO2
PSMC1	OR10AD1	ZFAND3
MAZ	C12orf76	PUF60
DOPEY1	UBE2B	FEM1A
FOS	FSTL3	ZNF581
PDIA2	CWF19L1	DEPDC1
TTC5	SLC44A2	NUP37
CLDN3	NOG	AGPS
EIF2B4	SNRNP40	SRP19
RP11-15K1!	ANKRD62	GAS6
PAX2	ZNF296	VTA1
CHST4	GAS6	BTF3
SLC27A2	CCDC51	SLC31A2
RFX3	GTF2E1	MACROD2
SUCLG2	AOC2	MRPS16
SH2D6	PNPLA6	ACVR1C
CHCHD6	RAD1	IGFBP1
FH	NES	OR10AD1
DACH2	CTC-260F.	NDST3
SUGP2	ORAI3	C9orf167
SEMA6C	NCOA3	XPO6
MAGEB17	PLAUR	SYT14
OVOL2	UBE2W	TMEM194A
BEX2	NRF1	ALKBH5
CCDC142	UBXN7	SLC39A14
CLEC18B	SIX5	SLC35A2
PRTG	PLSCR3	DYNLRB1
SERPINI1	PTS	WDFY1
BCAS2	MPDU1	PRELID1
ZNF280A	SDHD	RPL35A
ULBP3	E2F4	ELFN1
LGALS9C	WDR74	ATRIP
TAT	UBE2S	GALNS
CCNL1	ANXA2	TCP11L1
HLTF	LRRC42	SPTY2D1
GTPBP5	ATP5J2	SRRM1
ADRBK1	FBXW5	SIK3
ALOX12B	MMP21	RRAGA
DHX35	RIN2	DGKZ
MOGS	CA13	CKS1B
ZNF626	FAM122A	PTF1A
ZNF93	ENHO	GPR173

TSC1	C16orf52	SRCRB4D
TRIM58	NOXRED1	TIGD5
STAT2	BRPF1	NECAB1
MTL5	GJA9	ING5
MYB	PABPC1	PPTC7
ACOT11	IFRD1	RAD23B
ALG1L	COPS3	ZCCHC2
KAAG1	C18orf42	PRSS42
KCNH5	HTR1D	TMEM64
SUCLG1	PPP2CB	SFR1
DMRT2	OR5C1	43164
HTR3E	AVPI1	XAGE1B
IFITM1	FBXO36	SNX5
KRTAP10-5	HUS1B	YBX2
FASN	TMEM201	PSMD2
HOXB9	SRGAP2P1	TRIM47
MTX1	UBE2V2	ISL2
SULT4A1	TMEM192	XAB2
C20orf11	AP3D1	MECP2
MDM2	TLR9	LNP1
LRP1B	GRAMD1E	PRKACA
ELL3	RAP2A	NCOR1
LAP3	ADRA1D	APEH
PRAMEF9	TCOF1	EPN1
MARVELD3	C9orf167	CNOT3
CSAG1	FBXO43	SLC35F2
KLHDC3	SPINK9	SMYD4
CACNA1A	ACOT7	SNRPB2
TTC36	CYTH3	MAML1
HSPA5	LARP6	TRMT112
FOXI1	COQ10B	SDF4
TREX2	C12orf65	CCT2
SWT1	UBXN1	CHFR
PAQR9	CAPRIN1	SLC25A36
PLEKHG6	ARL9	RASIP1
HOXB3	ZNF570	REXO2
FAM123A	TOMM70A	NOP16
MSTN	DYNLRB1	APOA5
DDX1	INPP1	PAK1
OR4N2	SRRM1	ATL3
TMX2-CTNI	CLIP3	GNA11
MAGEA12	NAA15	PCDHGA1
CES3	GPR143	HDAC7
AC012414.1	MEFV	MAPK14
POTEB	TOMM22	NXN
CDY1	NELF	AFF4
DEFB131	IRGQ	ZNF347
IFNW1	COX10	PCBP1
OR2T12	KDELR1	C6orf89
CDY1B	METTL21E	RPS6KA4

GSX2	TUBB3	TEX2
TMEM30C	PPIF	GPS2
OR8J3	ATXN2L	C14orf178
OR10H5	OGFRL1	BRPF3
WI2-3308P	KCMF1	PRDM11
SYCN	RBMX	HRNR
OR2T8	HCFC1R1	CCDC90B
XIRP2	KIAA1958	USP4
OR14A16	CENPB	TRAIP
KRTAP19-7	CHEK1	PCBP2
OR2A5	PITPNM1	TMEM239
OR2Y1	MED6	C1QBP
OPCML	PCDHGC3	POLDIP3
OPCML	FOXE3	FAM165B
LCE5A	FAM108B	SUMF1
KRTAP20-2	ELK1	STK10
LACRT	ORC2	PCNP
MAS1	PRKRA	MED9
PRY	TMEM102	FAM196A
MRGPRX4	CHCHD3	SARS2
AC018682.6	NOA1	ME2
TAS2R42	ACTR1A	RPP30
CTD-2313N	WSB2	FANCB
GLRA4	DIRAS1	GNE
KRTAP19-3	SMG6	NME1
KRTAP19-5	SSBP1	YIPF6
RP11-58C2	NCBP1	MMS22L
APCS	TRIM52	RASA3
OR4K2	GIT2	ZFAND1
LIPF	RPA2	ZNF300
PRY2	UBE2D1	FBXO28
INS-IGF2	GTF2IRD1	CCDC117
GPR128	CDKN2B	PABPC1
NOX3	MRPS22	ZYX
HTN1	RAPGEF1	TRA2B
GRPR	GUCA1C	LDOC1
AMELY	NETO2	FJX1
ZNF776	VDAC1	SLC20A1
KIAA1024L	SLC16A1	MRPS27
CDK3	ELOVL3	CAMK1
GCDH	UNC119B	ACCN4
FAM134B	UTP11L	TCF3
TYW5	KIAA0907	SLC36A4
MAP3K4	KIAA1033	UBN1
GNPTAB	TMEM5	FNDC8
FRG1	CLIP1	CTRB2
OLFM3	ALKBH5	SS18L2
TEX101	CDK4	HIST3H2BB
CDX4	RUSC2	TIAL1
RSAD1	GTPBP8	GTF2H2

CDH22	DPY19L1	DHFRL1
C8orf74	SMS	HCFC1R1
ZSCAN2	CRCP	CYP51A1
ARHGEF11	ZFYVE16	SRSF3
TXNRD3	CREM	F12
MYBPC1	SLC10A3	C18orf42
AZGP1	TIGD5	ADAM17
KRTAP5-7	TUSC1	ZNF98
DIRAS3	SMAGP	NDST1
CCDC70	TATDN2	NDST1
CYP4F2	RNF150	B3GALNT1
PRL	C11orf73	GINS4
CCT3	HUS1	FOXE3
NOL12	CLCN1	MAP2K2
HSFY1	TMEM135	FASN
STOML2	POLR2J2	NUDT2
RP11-1000I	ZNF169	GDPD4
TPRA1	KIAA0355	NSUN3
CSTF1	HPDL	SUDS3
FAM32A	SEZ6L2	TP53
METTL7B	CREB1	SF3A1
TRAF3IP1	MT1P2.1	SMN1
PXMP4	POT1	TRAPPC2P1
TRIP11	KLHL7	F3
ETFA	S100A10	AL050321.1
ABCB9	TRPC3	RRP7A
RALGAPB	SERINC3	CDCA5
WDR18	RIF1	RNF4
DCTN1	RPL30	SP7
TIAM1	ZNF526	C11orf58
ULK3	COMMD6	ITGA2B
ASCL2	ZBTB9	PGAM1
MRPL16	MRPL4	ZDHHC15
BHLHA9	CCNY	BBS10
CENPE	HRASLS	USP36
EIF4E2	TEX261	GOLGA6C
TAF7	RBM14	DNAJC1
PLEKHG1	SAP30L	PARL
C2orf44	TARS	NEK4
IQCJ	C20orf112	MLH1
PDIA4	UCHL3	ZNF7
ILKAP	DTX3L	MTX3
FMN1	ENO3	BET1
PGM3	CALM3	HMGN1
BCAS3	RASIP1	RAI1
ADSL	TRIM47	MAP6D1
AP3B2	RAB1B	GNAL
KCTD1	AL050321	TUSC1
KRTAP5-4	VDAC3	ZC3H12B
VPS39	ZNF492	LBX2

MFSD9	C9orf64	FSCN3
ACTR1B	ZNF529	SETD5
HOXC9	NHEJ1	PROS1
RP11-108O	HIST1H3D	HIGD2A
GLRA3	KCTD5	RSPH6A
GLRA3	ALG3	U2SURP
DNAH14	CLN5	DPP9
CPSF4L	SLC25A37	MARK4
CCDC115	SMAD4	CSTF2T
GALR3	MMS22L	TTC39A
NR4A2	PTDSS2	ST20
NEU3	FNIP1	RAVER1
SUPT3H	HIST1H2B	RUSC2
CCDC88C	AP3M1	AP1B1
GPS1	AGPAT3	CYHR1
KDM3A	AGPAT3	GOLGA2
POU5F1B	PLAA	SMC3
RBM38	EIF1AX	COQ3
CAMP	C1orf85	PRLR
DDO	PSMB7	ZNF528
TMEM106C	C14orf135	RAD21L1
APOBEC1	DNAAF2	ELMO2
HSFX1	UBQLN2	RAX
OR2A1	ZFP64	QSOX2
SLC4A1AP	MYADM	RACGAP1
ELOVL6	PYCARD	FAM83D
SHC4	FAM131A	ZFP91-CNTF
KRTAP5-11	ANO10	MICAL3
ZNF134	DPP9	PSMD12
AZI1	FAM24B	RAB6C
SDR42E1	EIF1AD	RB1CC1
LRPPRC	YWHAQ	PELI3
IST1	ABHD14B	PRSS33
NOM1	RPS16	DMRTA1
CENPBD1	C11orf24	CDYL
C1orf172	MECP2	GSK3B
AHCY	PRX	WDR92
ISY1-RAB43	DNAJC9	RPP25
ZZEF1	AKNAD1	CELA2B
EXD2	C9orf123	MRPS6
L3MBTL4	CUL2	KRR1
FAM133B	PRDM11	PCNXL3
TRPM5	DDA1	CCL8
DNAJA4	TCTE3	DGCR14
ALX3	EIF3G	SMCR7
POTEC	NUS1	CERCAM
RGPD8	TTC24	TIGD1L2
SORT1	TGFB1I1	WDR62
VPS41	CAMSAP1	AIDA
ZBTB41	CDYL	INCENP

GORAB	TRAF2	ANAPC10
GOT1	LYAR	RPL35
GABRG2	HNRNPUL	NCEH1
FAM195B	ZNF527	FKBP10
SLC41A3	THAP4	VDAC3
WDR4	RPLP0	COMMD2
PCGF1	NR3C1	PFKFB3
AGAP1	STARD3NI	CREB1
GRID2IP	MAPKAPK	RP11-613M10.9.1
MUDENG	ZFP37	HECW1
RNF25	TLN1	YWHAQ
CTD-2611O	DRD4	CLPB
KRTAP5-3	SIK3	STRN3
TRIM15	CEP76	C16orf72
MORC2	PIGU	ERGIC2
PPP1R26	CNOT7	RPL15
MAP7	NDUFA6	RBFOX2
MUC20	RP11-603.	MRPS10
SLC30A9	STK11	DRD4
HMX2	G3BP1	CHEK2
GNA14	CEP41	EIF3H
UCK2	SLC1A3	PQLC1
NFS1	TONSL	SETP18
LGALS9B	PCBP1	ITGB3
ZKSCAN4	RGS10	TMSB10
CDKAL1	RRP7A	GORASP2
IMP4	ELF4	TMEM86B
WWOX	CHFR	PTPN20A
BSPRY	VASP	F2R
CBWD1	07-Sep	HHIPL2
MEAF6	C13orf33	CHCHD2
AQR	ZFYVE20	FSCN2
TMTC2	FBXO28	G0S2
HMGN5	HDAC7	HCN1
LRBA	ZNF383	RAB21
MOAP1	TANK	DUS3L
PREB	ROR1	PTAR1
USP33	SLC3A2	DSG2
NASP	C2CD4C	REST
RECQL5	PRPSAP2	RNF150
PEX16	VCL	MRPL13
KIAA0895	SNRPF	ZCCHC9
FAM19A4	PRELID1	SKA3
URB2	NAB1	PHLDB3
HMGB1	CCDC42	HIST1H4C
PRR5-ARHG	ZNF789	OR5P3
ODF2	C18orf26	RBAK
SNRPA1	TMEM41E	KCNG1
TMEM87A	BTBD2	BOLA3
OS9	AKT2	WDR45

ERCC3	C8orf85	CHURC1
MARS	PDE1C	LSM12
CHMP4C	BCCIP	MAST1
GDF11	SEC13	WDR70
NADK	SERPINH1	NME4
GLA	ZDHHC2	NCBP1
SLC28A1	FOSL2	COPS3
ZNF396	FAM82B	TMEM209
ACAD9	SMOX	MEFV
NGDN	RASSF1	FKBP1A
USP21	NME1	CBFA2T2
COPA	PPPDE1	TSC22D2
PLXNB1	USP17L4	DCLRE1B
IL4I1	EIF4H	EXOSC4
PLSCR2	SCRN1	ORAI3
SMPD4	TGFBR1	AFF3
CCDC120	NBEAL1	C8orf46
IARS2	ZNF354C	CD70
SLC25A4	NLRP9	CA12
RAB40B	WIPF2	COPS6
HEATR6	XAGE1A	C9orf123
ZNF434	KPNA3	DNAJC25-GNG10
CDC25C	DIMT1	PACSIN3
XRCC6	CHERP	TEAD1
SORD	INTS6	EXOSC5
NDUFS1	BAX	ECD
BSND	WFDC8	ZCCHC7
PAXIP1	SUDS3	ACAN
TRIP4	PPP1R11	NAMPT
GGT7	VTA1	SLC2A8
RCCD1	SRMS	DNAJB5
CCDC14	SF3A2	NBEAL1
CASZ1	HIST2H3D	FAM208A
NMNAT3	RAD23A	LARP6
PCDHGA1	PRDM8	RNF2
KISS1R	TCF7L2	LAMP1
SPIN2B	SARS2	SGK196.1
CLDN8	CYP51A1	MMGT1
IGSF8	FSCN2	C1orf144
ZFYVE26	TUBG1	SH3BGRL3
PRPF4B	IAH1	MAMLD1
HOXB1	TM6SF2	ASAP3
UBA5	C11orf48	FKBP1C
RBBP5	ZNF300	CCT4
ARID2	ATG14	RFNG
WISP3	PDS5B	ANKRD33
EYA3	ZBTB16	JAGN1
OR10H1	C7orf11	SCAMP1
PPIG	CTU1	TFAP4
HGS	MAFF	NCOA3

SYNJ2	RPE	FBRSL1
RCOR3	IGFBP3	NUDCD2
SYNE2	MFSD5	EXOSC2
SYNE2	FBXO45	WDR53
HOXC10	DNAJC21	TNIP2
ZNF84	VAPB	CEP57
ZNF670	LPAR2	PTPN11
LCT	NHP2L1	CAMSAP1
STAG2	PPP1R2	ABCA1
C6orf222	ROBO3	A4GNT
HOXC5	DDX56	SLC35B2
NSL1	SYT6	RPS18
GPR35	SLC30A1	TRIM65
FARSB	STAT1	STAM
SOX13	PIP5K1A	RAP1GAP2
MLL3	RSPH6A	ANLN
CHRNA1	PRKACA	C14orf109
STXBP4	CSRNP2	YME1L1
CCT6A	PKM2	UBE2V2
ADCK2	C9orf102	NACC1
PLA2G15	KIAA0226	PAK2
ARSH	NMD3	CLEC11A
GOLGA8A	HCRT	FAHD1
ACTR5	LRRC40	SLC6A15
USP28	NFIC	CHTF8
RP11-382B:	CUL4A	NUP107
ZNF772	FAM207A	MZT1
MVD	ETF1	AL928654.7.1
NOTCH3	CA1	CDR2L
KIF21A	FAM151B	XKR6
TTLL4	EPB41L4A	NAB1
CLDN23	UBE2I	HEPACAM
BHLHB9	LHPP	L2HGDH
C2CD4D	EPHB4	TRAF2
PHF8	MAPK12	PAQR4
ERMP1	PEX14	GPR3
APEX1	TRIM32	PUS1
LRRC8D	PDZD8	IPO11
TTF2	GAS2L3	MEA1
ZNF549	RPLP2	ZNF492
SDHAF2	OR13J1	C7orf30
MAP1LC3A	ZNF791	RPGRIP1L
PPP1R1A	NECAP2	PTDSS1
TRIM10	RFX1	C20orf4
OTUB1	MIR3654	KRTAP10-2
KIFAP3	HIRA	EWSR1
DAAM1	CXorf69	NUFIP1
ACSM5	SETP18	ZNF333
TMPRSS5	NAP1L1	KCTD15
PBX1	PARS2	CFL1

PBX1	CSNK1E	TEAD2
GK5	DNAJC11	KCNK13
ATIC	DPY19L4	TAF6
TOR3A	C17orf67	SNX32
TUBGCP4	KLF17	RG9MTD1
PAK6	INS	RAP2C
DDX18	RP11-463I	ZEB1
ZKSCAN1	SGPP1	AMFR
APBA2	STAU1	ZNF324
SETDB1	DMKN	PPIL4
TDRKH	MRPS10	EIF2C1
RNF43	RPL10L	HTT
SFT2D2	HOMER1	TPGS2
CRISP3	GDI1	RALB
CEP63	VHL	RAD23A
TIMM17A	MINPP1	YWHAZ
BCS1L	FBRSL1	VWCE
PCNXL2	UBE2L3	RP11-540D14.8.1
SEMA4F	CENPA	C16orf61
STAMBP	WAC	MEX3C
FAM63A	YJEFN3	ZC3H3
GPR56	LSM2	SRPX
LRPAP1	ADAMTS1	PUS7
ITFG2	MED26	FAM19A2
NARS2	CNBP	C19orf68
RBM41	ZBTB43	S100A16
POLR1B	IL17RE	MTCP1NB
ACTL10	CD8A	CERS1
UBE2L6	REST	TUBA4A
GCHFR	TCTN2	AC003682.1
ASPM	ELOVL5	CRY1
PSMD7	CHCHD10	CWC22
DERA	UBE2A	DFNB31
VAX2	CNGB3	BACE1
TSSC1	XRCC4	MRGPRX2
C12orf50	SLITRK1	GFOD1
MKS1	FTH1	GFOD1
NIT1	GNAQ	USP53
TADA1	RQCD1	DENND1A
KRBA1	ANKRD30I	PTS
COIL	GHITM	SLC25A38
MRFAP1L1	ZCCHC6	BATF3
FAM115A	FAM9A	ZCWPW2
OPRK1	RAB14	CYP26C1
RAB6B	C14orf142	C7orf73
PNN	PDRG1	CEP76
ZSWIM3	PFN2	C14orf142
VLDLR	FKBP10	PARD3
OSBPL2	RP11-87C	CDK6
OPTC	IL5	MED26

MAN2A2	SUMO1	OPA1
RP11-831H	CLEC18C	IGF2BP3
AGPAT3	ELL	ATF6B
AGPAT3	CSRNP1	PPP1R18
PRAME	MCTS1	C9orf86
ALS2CR11	SEC61G	ASB14
ACACB	TMEM123	RBMS3
WDR3	RALY	PRKRA
KDM4C	TSR1	CTDP1
C14orf133	FOXJ3	REP15
C9orf140	GLIS3	MRPL32
TSPAN16	ATP5L	CLTCL1
ZNHIT6	POLR2K	GTF2H2C
SCYL3	PPARD	SUB1
ORC4	ZCCHC14	ATP13A3
PLEKHB2	ACTN4	APOOL
PPP6R3	CCT5	HMG20B
CCAR1	NUDT15	FAM164A
TRAPPC11	REP15	NEIL3
DNM1L	PRKAG1	IMPA1
PSME4	MICAL3	MKLN1
TIPRL	IFNG	C5orf41
HOXC4	B4GALT2	ADAMTS17
DEDD	GOLPH3	FXR1
C17orf110	STK25	RP11-603J24.9.1
DARS2	BSX	ZIC2
ZNF3	TLK1	PTPDC1
SLC4A3	TAP1	UBE2W
C5orf44	FRMD5	PTP4A1
DNMT3B	KIAA1826	POLR2L
POTEJ	BCL7A	ZFP36L1
DIAPH2	TRIM14	TUBG1
ZNF496	FHOD3	PACRGL
ZMAT2	UBE2N	ABHD14B
TSNAX	NCOA5	SLC12A5
HES6	MBLAC2	PLAC8L1
CPN1	CDK7	CCDC120
BAG5	NSMAF	IFITM5
TMPRSS11E	BATF2	PDCD2L
C1orf115	PDDC1	C11orf68
SERF2	HIST1H4C	FCRLB
ZNF107	SRP19	SACS
RAX2	CHCHD2	MPDU1
REPIN1	GMEB1	TRUB1
INTS7	DPF1	B3GALT1
PCDHA1	C11orf46	MPRIP
EDC3	RAP1GAP2	RBM3
BUB1	GAL	TMEM39A
TBC1D16	SLC25A3	ZNF852
PRPF38A	IQCJ-SCHI	EHD1

ITGB8	NDUFB9	GAPVD1
MTF2	POLE4	MAGEE2
MAP2	C4orf29	SLC35E1
PMS2	WNK3	TLL1
MKRN1	TNFRSF1A	MRPL16
ATG4A	UROS	ATG14
RPUSD2	ZNF268	ACSL3
FAM170B	CBLB	COX15
CHMP6	SPAG11A	GRAMD1B
MYPOP	COX7B	DNAJB2
OBSL1	ZFP36L1	C14orf39
SPG11	RAB3D	PBRM1
KCNH2	XPA	NFIC
ANAPC1	ENO2	SENP5
ARID4B	CDK2	RALY
MTSS1L	TIMM50	MAGOHB
SERPINA4	TPX2	HAT1
ZNF45	ULBP1	PPP2CA
PEX19	VSTM4	PAFAH1B3
ATAD2B	GPRIN1	AMDHD2
MPI	TWISTNB	OGFRL1
IDH2	CST7	FAM171A2
HSPD1	TMEM161	TAF1A
XYLT2	PLOD1	EFEMP1
ELF5	PSMD12	CGGBP1
RWDD2A	ASAP2	C5orf39
CHD7	CGREF1	H2AFX
KBTBD4	C5orf45	TRAF7
NOL10	UPK3B	C5orf35
DDX47	SLC20A1	PSMG1
GDAP2	FBXL5	MT1P2.1
SPRY3	TAB2	RAB11B
ANGEL1	ZHX1-C8O	EIF4G3
KANK1	CDC37	RAB6A
GYLTL1B	FBXO41	SRGAP2P1
TET3	BAG6	LYRM4
FAM82A2	FUNDC2	EHD4
TMEM187	DDX51	NUCB1
OR2T33	YPEL1	MAD2L2
USF1	ZCCHC24	TUBA1C
POU2F1	UBLCP1	FKBP4
TNRC6C	AC091153	WDR78
COG1	SH2B3	PSMB7
PDK3	DNAJA3	CRTC2
CDAN1	FAM213B	FBXO3
KDM4A	DEPDC1	CENPP
PSMD14	NICN1	TEAD3
APOA1BP	CDK9	POLD3
RPAP3	ACVR1C	EPO
ATG4D	MTRF1L	RNF5

POGK	USP19	EOMES
B4GALT3	GATAD2B	NSMAF
CLASP1	FAM89B	TBCA
UGT2B10	PABPN1	TTPAL
KLHL12	IMPAD1	U2AF2
CCDC129	PPP3R1	KDELR2
SIPA1L1	SAE1	TGIF1
DDI1	ZNF506	GALNT2
CCDC158	KRTAP10-	ATP6V1E1
EHF	BATF3	ELOVL2
ABCF1	RP11-111I	RPL36
GALK2	SCML1	GDI1
ZNF92	PRRT2	NDRG4
KIDINS220	HMGN1	ARHGAP39
IGSF9	PSME3	DPP6
MRPL12	MMACHC	BRD2
LGMN	KCNE2	EFCAB11
PDIA3	GNB4	C5orf45
ATP6V1B1	LRFN3	ACVR2B
GLYATL1	ELAVL2	C1orf195
TRAK1	APOA5	PRR19
AQP12B	HS3ST3B1	GNB1
HIP1R	ST3GAL3	GPI
ZNF77	ABI1	ZNF460
PHKA1	LNP1	SUMO4
GPC4	XAGE1B	CA5B
EPN3	TUBA1B	ZNF665
DNAJC22	TIAL1	MAN1B1
DBR1	CIRH1A	SLC44A2
NARF	ZNF430	SLC39A10
MED12	HSPA14	POLRMT
TAS1R2	RPL27A	CASP2
PFDN2	ZNF551	WDR75
C7orf53	MIR4720	PDHB
TAF11	SMAD2	SYS1-DBNDD2
C7orf49	BSG	PPP2R1A
H2AFY2	MRGPRX2	CIB3
EIF4A3	CENPV	ATP6AP1L
AADAT	AP1B1	ZNF655
POGZ	FBXO33	SMC4
PODXL2	PLCB3	YEATS4
DHCR7	RNF10	CNFN
HERC1	ZBTB4	C15orf52
RPTOR	MACROD2	RPS10
MKRN2-AS1	EXOSC5	CXXC5
HSP90AB1	AGFG2	TMEM167A
MTERFD2	C16orf96	SMC1B
MESDC2	C16orf96	OXTR
SLC5A6	C16orf96	BNC1
CACYBP	LDLR	AGPAT3

ANKRD37	ERCC1	AGPAT3
GYG1	ITGB1	ARHGEF33
ZSCAN29	TMEM185	PHF16
MID1	TOMM20l	C7orf61
KDM5B	PEX2	PEX14
C20orf151	MLLT10	KIAA2022
ERVW-1	TAF5	CLPTM1
MEN1	PROZ	43163
FBXO9	C19orf53	GMEB1
NFIA	FUT11	THAP1
RRAGB	OLFM1	MAD2L1
FAM187B	CAB39	GORASP1
TP53BP1	TADA2B	PSMB2
SLC6A19	TBC1D4	ZNF131
PHF16	ASS1	CLIC3
MTMR4	ZNF461	ACOT4
ZC3HAV1L	UBQLN4	SLC25A5
VRK2	C19orf57	MKI67
ZNF749	NUP37	BDNF
ZKSCAN3	RNPS1	PDZD3
SNX4	PSMG1	CDKN1A
EEFSEC	KCNK12	C1orf31
TBC1D7	TRERF1	C1orf135
NR4A1	EAF1	C11orf84
KLHL25	GNAI1	CPSF1
PLCXD1	PFKFB3	HSPBP1
ARID4A	AC068533	C16orf13
NEB	HEG1	TMEM102
RSRC2	FAM45A	SUV39H1
HOXC11	C1orf21	PRSS37
PHLPP2	DOLPP1	MYADM
ZNF136	KHDC1	RBM27
PSRC1	RER1	RPL29
ABCA12	ABLIM2	EEA1
METTL13	NKG7	LDLR
NCAPD2	RAC1	C3orf55
NDUFS2	ACTB	GTF2H4
SNUPN	TIMM21	TRAK2
USP13	MASTL	SNAPC3
ESCO2	RPL32	NAF1
SERINC5	C20orf3	CYBRD1
CNDP2	ERP29	PFDN6
ZNF124	TMEM200	PEX6
FASTKD2	WDR43	SLC6A8
HTATSF1	LPCAT4	RCC1
C3orf33	FAM175B	SIRT6
SNRNP200	ALX4	CHRAC1
SLTM	EWSR1	CDYL2
GPN1	TPD52L2	STMN3
SBK1	EDEM1	TPST2

GTF3C2	PRPF19	HTRA2
CENPM	GFER	CASC3
CIAO1	SPAG4	PDSS1
SV2C	ARF3	EGFL8
C2orf68	TLL1	KIAA1958
ATF6	GLRX5	TYW3
C10orf113	A1BG	TUSC2
PPP2R1B	ZDHHC20	ASS1
RAE1	RFC5	MTHFD2
HSD11B2	STK3	ISG20L2
CAB39L	MAPKAP1	TRIM52
LYSMD1	NXPH1	THAP8
C15orf23	ST6GALNA	ROR1
SNX1	RPS23	CTD-2116N17.1.1
KDM1A	ATP5SL	LSS
H2AFY	PLBD2	SLC39A1
TJP2	ALG10B	ARNT
OTOG	FSD1	DGKH
ALS2	XPO6	C3orf33
NEK2	IGFBP6	C9orf64
TTI1	BNC1	CDK2
TDG	TMEM145	SLMO1
PUM1	ANKRD57	UQCR10
WDR45L	ZER1	RUSC1
JARID2	CTD-3193	DIAPH1
ZSCAN21	RAPGEF2	SCAF11
LMBR1L	DAPK3	APOBEC3B
HIPK1	PRSS23	DAPK3
ZCCHC12	RBM27	C1orf187
MYH2	C6orf162	C2orf47
WDR61	HCFC1	BTNL8
WDR61	AP002884	SPIN3
C1orf105	ANO7	TMEM41A
HDAC8	SHOX2	IRGQ
IWS1	PLXNA1	ABI2
CSTF2	POLDIP3	CNNM1
DTNB	RBM15	ILF3
UPF3B	OR5P3	FXN
MTRNR2L7	C2orf49	AC008073.5.1
LEO1	FRS2	MRTO4
ACO2	FBXO30	TMEM136
VIL1	RPP14	UBQLN4
C12orf48	OPN1SW	MAP4K2
PRRC2C	PDSS1	MLLT10
C6orf223	CAP2	COMMD9
ANP32A	CDCA7	MAP4K4
UBE2O	ROR2	CCDC92
TUFT1	SLC39A1	C20orf112
NEDD4	RAB11B	GPR150
KLHL20	SUN2	DIRAS1

ARHGAP11/	PAK2	AKT3
CLP1	SLC35B3	CENPO
RPAIN	METTL9	KCMF1
CEP135	C11orf30	GARNL3
HOXC6	AC012652	FLNB
SLC1A2	CLSPN	PLK4
MYLIP	FEM1C	ATP5J2
C17orf80	ATOH7	TOMM5
CHD4	YAE1D1	WTIP
TRIM24	PITPNA	PLSCR1
PHIP	SLC23A3	FSTL3
C7orf34	TRIM65	TEX264
TAF1	TMEM88E	C3orf22
YLPM1	ZNF730	SHB
SRBD1	SRD5A1	CCDC112
SLC25A2	SHOC2	RNF135
C15orf44	CPNE1	AGPAT6
CENPL	ECSIT	B4GALT5
PRPSAP1	CRBN	AP3M1
SMUG1	03-Mar	MRPS25
DLGAP1	SERF1B	ENO3
CCDC137	LMBRD2	PLRG1
PGK2	RBM18	FDXACB1
TOMM40L	NRBP2	C5orf30
KANSL2	POLR2L	PITHD1
RIC8B	C17orf79	METTL22
SPTBN2	RABL3	PER1
PTPLAD1	TRIM28	MRPL41
EGLN3	CEP97	SLMAP
ZNF398	AJUBA	BSDC1
LNX1	CD68	CDK16
SMG8	SLC35E1	MRPS12
DIS3L2	C3orf17	TCF24
HSP90AA1	SPATA21	TGOLN2
COPS7B	SNX16	C7orf42
ZNF184	A4GNT	FGFR1OP2
NUSAP1	GSS	FAM70A
DPP3	YIPF6	C3orf39
HBM	C10orf12!	OSBPL10
CABP4	BCL3	CUEDC2
TRMT2B	HEXIM1	RAD54L2
NUF2	HN1L	SPINK9
STYK1	ZSCAN5B	NDUFA6
TFCP2	NOP16	FEM1C
C12orf51	PHF7	ZFP37
PIF1	ATP6AP1L	FUBP1
FAM5B	RG9MTD1	CNOT2
NEURL3	PRTFDC1	UCHL1
CCNB2	PRR11	TAPBP
IVNS1ABP	PXDC1	PAFAH1B2

ACPL2	MRPL50	GNS
GLYATL2	C9orf131	BTBD7
BAZ2B	APOA2	LYPD5
MFAP1	ACD	UBE3B
AAGAB	RNGTT	SMNDC1
DNMT3A	AURKAIP1	ANXA7
PRC1	NOL7	STK3
ZWILCH	ATP5E	TADA2B
JUN	CHAMP1	ZC4H2
KIF23	HAT1	CHAC2
C1orf112	ZNF668	B3GNT9
	ZNF490	BORA
	TTC39A	ZNF354C
	CXCL5	PDHA1
	CDCA8	MRPS33
	C1orf216	GREB1L
	AMPH	ENY2
	RPF2	RPN1
	SSFA2	TNFRSF9
	LMOD2	FNDC3B
	KCTD15	ECHS1
	TERF2	MYOM3
	PPAT	TAOK3
	CENPP	C20orf30
	YTHDF3	GOLT1B
	USP7	43162
	STRN3	FAM116A
	WBP5	SPIN4
	STXBP1	FAM213B
	ZNF581	C11orf82
	LSM5	HCFC1
	MAP1LC3I	PDDC1
	CLOCK	NUDT19
	SPTBN4	PTDSS2
	CRTAM	CMC1
	SETD1A	SPTBN4
	STK4	C20orf20
	PROM2	SSSCA1
	SRSF3	ZFP30
	RAI1	TCTN2
	TMEM65	TMEM126A
	COX7C	TEX261
	ZNF408	PEF1
	DSG2	PEBP1
	ACER1	CEP89
	NXT2	SUV39H2
	LDOC1	LAG3
	YBX2	SEPHS1
	AL034548	TMEM93
	HBA2	A1BG

C3orf22	ENHO
TMEM18	MRPS36
TPP1	HUS1
CKAP2L	RNASEH1
DHRS4	CDCA3
HNRNPF	TMED7
GPR180	ZBTB43
APOBEC3(	DDAH2
ARFIP1	PFKM
TTC23L	C19orf79
AC078802	LARS2
PTPMT1	ZSCAN5B
NSUN2	VASP
RAB18	CELF1
CTDP1	CELF1
AC069368	RPRD1A
C12orf49	PABPC4
DGCR2	CALHM3
TNPO2	SH3BP2
CAMK2D	ZFYVE16
PRR13	KHDRBS3
PTPRH	ACVR1
GNS	KIFC1
POLR3A	TDP1
PDZD11	SMG5
PPID	TOP1MT
PIK3C3	KRT85
C19orf33	OXSM
OXTR	CYB5B
C9orf86	C16orf91
FAM214B	PRRX2
SDF4	MKI67IP
PRKCDBP	SNF8
C12orf75	EPB41L4A
EIF5AL1	FANCG
IMPA1	C20orf96
C15orf52	ILKAP
NCEH1	GCSH
RPS9	CSNK1E
ZNF639	SYNCRIP
PPP1R14A	ZNF730
ZNRF2	VIM
TSFM	CAMLG
ATP6V1E1	CCDC154
B4GALT1	SURF6
CASP2	PDZD11
CDKN1C	BDP1
TFDP2	LCTL
MRPS33	FAM89B
ATF1	CDC45

MTG1	ZNF791
GPR63	ERBB2IP
BSDC1	DUSP7
LAG3	ZNF585B
MDC1	C3orf17
CDIPT	SGMS1
C1orf135	TLN2
FLG2	TLN2
TMEM194	BCL7A
MAMLD1	GTDC1
DNAJB2	ZNF26
PRKCA	ZNF428
ZSWIM6	ZNF668
UBE2G1	AMZ2
CDK1	MAFA
AC100775	BCL2L12
FTL	TTBK2
MKKS	ZIC5
C16orf5	EDIL3
C16orf5	GPRIN1
ARHGAP1	TMEM147
ARMC12	ZNF628
ERCC8	TSPAN10
GPANK1	SNX33
CCDC92	HNRNPM
NGLY1	ZNF167
KRAS	CETN3
C8orf46	RGP1
URI1	RP11-305M3.3.1
ATP2B3	RAB4A
CEP57	C9orf69
ATG13	FOXO3
TRPA1	SURF4
PLEKHA3	MPO
DCAF15	GSK3A
PRKCI	SSBP1
KIF20A	SGTB
ZW10	C17orf81
WASF2	EIF2AK4
PPP1R42	SYDE1
PACS1	SPCS1
OVOL3	MYNN
SF3B5	ARMC12
PTPN11	CIITA
C19orf68	ETHE1
SOX18	RP11-77K12.7.1
RNF114	EIF2S2
WTIP	P2RX4
RUSC1	LZTS2
CCDC80	MTRR

PRKAB1	TLCD2
NNMT	AC068533.7.1
ATP6AP2	SLC25A37
TSEN15	ADAM20
FAM18B1	YAE1D1
TIMM44	C1orf122
TRUB2	ERI1
HTRA2	HERPUD2
NDUFAB1	ZCCHC6
RPS2	CYTH3
FAM19A3	SUSD3
ATP6V1G2	SPPL3.1
EHD4	ORC3
KCNK2	FAM49B
C1QL3	VSIG8
ABHD10	KIRREL2
FNBP1	NAT10
RAB4A	GNB4
MT1F	PFKFB4
CSRP2BP	FKBP14
SCOC	FAM168B
ST20	ZYG11A
NRBP1	LRRIQ3
SLC36A1	PURA
RWDD1	RAD54B
PAFAH1B1	PJA2
CHAC2	TCEB3
B3GALTL	CAMK2N1
ERGIC1	SIM2
ADNP	CFL2
NANOS1	ZC3H18
C8orf58	DDX49
SMC3	KIF15
LCTL	PPP1R8
SUMO4	OR13J1
EIF4G1	SLC2A14
ING5	PLBD2
HSPA4L	C6orf48
GSPT1	PPP1R42
MARVELD	NCOA6
BANF1	ATP2B3
GFOD1	ORC5
GFOD1	VHL
TCF3	AIMP2
TMEM22	VAPB
AIDA	TMX1
SHARPIN	ARMC1
TGOLN2	CTXN1
RAB11FIP	DNAJB1
PPP2R2C	C17orf79

LZTFL1	C11orf74
HNRNPUL	SERINC2
RHOBTB3	KIF5B
BICC1	KDSR
TOMM6	NMNAT1
PPP2CA	SGCE
HBA1	PEX10
C8orf82	EPHA6
GDPD4	DDX54
ZNF140	GBP1
CETN3	DCTD
MAP7D1	GPR153
MAGEE2	HIST2H2AC
OBFC2B	CXorf69
C12orf66	KCNK3
RPS6	RLIM
AMFR	CPPED1
MOB3A	ZNF564
CTSB	NAPA
CXXC5	MAP2K1
DNAJB4	OBFC2B
FAM64A	SDC4
UBTD2	AACS
STRAP	EIF2C3
GALNT10	GPX8
RFC3	GPR63
TEAD2	PIGT
PRPS1	AKIRIN1
LHFPL2	FBXO36
CEP89	TRMT1
M6PR	NUP43
G0S2	RPL14
AC011498	ICAM4
C6orf72	RBM15
HHIPL2	TMEM161B
FTSJD2	UBAC2
MRPS17	RAB3B
C19orf10	CDC23
DAND5	NDRG3
CYP26C1	ZNF562
GULP1	ZNF584
ZDHHC15	PWWP2B
CORO2A	TRAPPC5
PTCH1	C2CD4C
ENTPD7	TMED7-TICAM2
SLC16A8	CDC123
IFI27L2	WRNIP1
FANCG	CECR6
AKIP1	ZNF345
ANKRD13.	SCAF1

MED29	PLK1S1
COPS6	RPS11
NIPSNAP1	NXT1
C6orf89	CPSF2
CDC25A	FAM98A
C4orf52	GIT2
C19orf42	NELF
ATAD3A	COL4A3BP
ACAN	CYB5D1
GAPVD1	HEXIM1
CASP5	CHURC1-FNTB
DALRD3	CECR5
SNX3	PIEZO1
YRDC	AS3MT
BCAP29	PPHLN1
GPR3	TNFRSF1A
CUEDC2	UBAC1
TROAP	LASP1
ARMC6	HIST1H2BD
DGCR14	SPAG4
TSEN34	PPFIA1
CCDC165	ZNF569
DUSP15	ZNF251
BSN	CENPQ
MEIS3	PHC3
GSR	APOBEC3G
KIAA1704	TARDBP
ZNF771	SMOX
TMEM242	CSRP2BP
CMTM3	NANOS1
CBFA2T2	EXOC1
DCTD	AURKB
MANBAL	PFAS
STK24	PANX1
EFCAB11	C5orf54
CD244	HDAC4
NOL8	ECSIT
RTN4	SLC1A3
PML	THUMPD3
ACTL6A	VSTM1
PPIL4	STRADB
ZNF251	C8G
ITGA2B	AXIN1
KDM2A	MBLAC1
MRPL21	HBS1L
ALG2	IBSP
CREB3L1	PSKH1
GLOD4	FAM82B
ETV6	NEK1
AP4S1	RNMTL1

PRKCSH	FUNDC2
HIC1	PFN2
COX15	MTRF1L
SMYD4	MEF2BNB-MEF2B
MFI2	CCK
ADAM17	HIF1A
LRRC3C	HIF1A
PTPDC1	C19orf40
TRMT6	CXorf56
TCF24	TNPO1
NECAB2	TRIM14
LYL1	NTPCR
OSBPL10	PLOD3
C9orf46	OCRL
CLTCL1	RECK
TBX21	RNPS1
MFAP3	ARPC5L
RP11-526I	B4GALT2
ZFAND2B	ANKAR
MMADHC	SLC25A3
EIF6	F8
GP1BA	TMEM55A
FBXO3	SSRP1
AC003682	ALYREF
ST3GAL2	IARS
MRPS16	ADRM1
SATB2	SMARCA5
TOMM34	CD109
PRKG1	DNM1
HSPB11	TMCO6
HIATL2	ANPEP
SLC9A3	MAP2K7
PPP2R5B	PTPRH
CHKA	RP5-977B1.10.1
NMNAT1	ZBTB7A
THOC3	WDR41
ARMC1	MIDN
HRNR	EP400NL
TEF	ELF4
CFL1	RBM10
NEDD8	C9orf80
CCDC72	SLC13A3
HM13	TGFBR1
TRIM56	DCAF4
S100A16	B3GALNT2
HELLS	THY1
APEX2	MAPK11
HHLA3	REEP2
RPL36A	KIF11
BRPF3	TPM2

C14orf21	MRPL18
TRIM8	CYB561D2
JPH2	CD68
RP11-613I	LCN1
ZMYM1	LRRC59
MRPL41	AC069368.3.1
TXNL4A	CPSF6
LRRC47	GIT1
TGIF1	RAB8B
USP6	PCDHGA11
PDCD2L	STARD3NL
EXOSC2	PEG10
MTCP1NB	NNMT
RPL8	OSBPL6
INPP5A	MASTL
AASDHPPT	FAM123B
USMG5	DPYSL3
RUVBL1	FRMPD4
ATP6V1G1	ST3GAL2
TRAM1L1	PSMG4
EIF3F	GALNT10
TBC1D10A	CCDC66
OSTM1	SLC35C2
RB1CC1	RUVBL2
UQCC	FAM24B
ZRANB1	USP30
JPH4	PSMC3IP
GPR126	PINK1
GIT1	PGK1
HNRNPH3	RPL7L1
C3orf37	PROZ
KIAA2022	CATSPERG
HIST1H2B	CMTM7
MPV17L2	CDK1
ZNF70	CDC34
ORAI2	RNH1
MOCS3	SERTAD2
SAV1	PYGO1
GOLGA2	AP005814.1.1
UQCR10	MCF2L2
HIPK3	KLK1
ANKAR	KCTD6
LTBP4	METTL21B
C4orf27	SLC25A18
CDC37L1	URI1
ZBTB26	MMAA
CCDC71L	CCDC144A
EIF2B1	LENEP
MDGA2	UTP23
ARNT	SHOX2

DIAPH1	RAB2A
SEC24D	UBTF
TMTC4	ATMIN
RBPMS2	SFMBT1
ZNF622	ZNF625.1
HMHB1	OR5C1
KHDRBS3	RP11-770G2.3.1
ZNF665	UGCG
RC3H2	COQ10B
FAM127B	UBL4A
PIGO	CCDC50
BTBD3	SPATS2L
C8G	PPP1R37
SWAP70	DIAPH3
WWC2	OSGEPL1
FIP1L1	RP9
ACTR3	RAB10
FNDC4	COL20A1
NCOA6	ZNF768
GFM1	DNAJC30
RP1-239B	TMEM135
LATS2	TAF10
GLRX2	ADRA1D
ELP4	DHODH
MEPCE	MDGA2
TRMT112	
SEC24A	
TRAF7	
TMED1	
ZNF131	
SLC6A15	
ACTR2	
LYPLA1	
MRPL36	
ZRANB2	
HOOK3	
RPGRIP1L	
ALYREF	
CERS6	
NFIB	
HEMGN	
SLC2A14	
FKBP9	
BRD3	
TRAIP	
YME1L1	
HSPA13	
ZNF121	
RPL37A	
PDGFA	

SUPT4H1
KIAA0494
PUS7L
UBXN6
C16orf74
DGKZ
CSE1L
SLC35G3
KLHL9
ARCN1
ESF1
SPIN4
SRSF2
RNF38
TPRX1
TTC32
SLC20A2
RPUSD3
MAP2K2
C8orf45
MCM8
SYNCRIP
SLC2A8
08-Mar
CCDC154
PNRC1
RPS27L
RAB33B
PUF60
WDR45
ZNF585B
C1orf31
PARD3
NDRG4
F12
MRPS27
UNC45A
UNC45A
POMZP3
HOMER3
ARL1
TBCEL
PTPN20A
PGAM1
POU4F1
EIF4EBP2
SREBF1
CSPG4
WDR62
C3orf64

SLC38A9
SLC35D2
SUSD5
DSE
PSMD5
ARHGAP1
GUCA1B
SGPL1
MRPS12
CA9
RECQL4
OSBP
AMDHD2
RP11-691N7.6.1
GRPEL2
WASF3
DCLRE1B
RP11-305M3.3.1
MBD2
SETD7
DEPDC7
PHF10
BORA
CNOT4
C11orf1
GRPEL1
TPH2
PLSCR1
DFNB31
ACR
ZFP30
XPOT
TEAD3
FAM70A
KIF5B
TSG101
NIT2
RNASEH2A
RPS6KA4
U2AF1
SF3B4
ADARB1
SRSF10
MCF2L2
TRA2B
RYR2
CHMP4B
PAFAH1B3
CAMK1
UNC119

MRPL42
HIST2H2AC
LAPTM4A
CTD-3074O7.11.1
CYB561D2
CDC45
PSMA2
C19orf43
EFNA2
FAM171A2
KCTD18
VEGFB
TRIM74
SNF8
PARVA
ORMDL1
REEP2
NDUFS8
EFHA1
RNF216
GPX1
ADAM9
MRPS2
CHRNE
SERTAD3
JOSD1
C7orf30
DDX55
FAU
DCUN1D3
SLC13A3
CHUK
SEC61B
PANX1
APOBEC3G
VAMP7
AP3M2
ICAM4
SLC16A3
DLK2
TEFM
RUFY2
RMND5A
NLRP6
TBP
RBBP4
C8orf38
NEDD1
DDAH2
RACGAP1

SIM2
IGF2BP1
FRMD6
ORMDL2
NIPSNAP3A
JAGN1
UBN1
CNN3
C9orf25
G2E3
GANAB
NAGS
PAIP2
AKIRIN1
FOXN2
OGFOD2
AMZ2

Supplementary table 4: List of negativaly coregulated genes with top5 LncRNA

**RP11-480I12.3.1**	**PVT1**	**RP11-783K16.5.1**	**PPP2R3B-AS1**	**RP11-359M6.1.1**
TNFRSF6B	EIF3L	OR6S1	RAPGEF2	COX5B
SYT15	KLF15	ICAM3	PHC3	PIGV
PRKCA	ZNF211	JAM3	SH3BP5L	BIRC2
DUSP18	DDX59	LRMP	FAM127B	IGFLR1
UFM1	DHRS9	C1R	VPS37B	SLC25A23
PSMG2	DPEP2	IL17F	HNRPDL	CTF1
FAU	TMCO4	NLRC3	TMEM129	AQP8
P2RY13	RGAG1	FUT7	KCMF1	CIITA
HBA1	DOCK6	CD2	TMED1	XKR9
ENOX1	SIL1	CNR1	EGLN2	ZCCHC4
ARHGAP9	RG9MTD2	ITGAX	PRPF19	IL1RL2
LHPP	ANKRD18A	VAV1	GNB5	PRRT1
C19orf54	PDZK1IP1	PTPRD	LSM6	EGFL8
MSRA	MYBPC3	F10	CXorf56	SIGLEC11
MSRA	RAB36	SEL1L3	SLC22A15	C5orf60
FYN	SLC6A1	HBE1	PRMT10	NKIRAS1
NNMT	ARSA	LILRB1	KRTAP10-2	KIAA1522
ARGLU1	ITPKB	SEBOX	RWDD4	ATXN2L
SSC5D	WFS1	PTPRC	DHX33	PITPNM3
PAN3	CLSTN3	KIAA0564	SHMT1	RAB6C
IL6ST	ACE	COQ4	CCDC132	SDC4
HBA2	ASB3	ARHGDIB	PREX2	RSPH6A
PRKCDBP	SULT1C2	NEK3	ZBED1	CORT
XKR9	ATP1A2	PLA2G7	STON2	FOPNL
RPL13A	RTBDN	TMEM176A	C19orf6	RANBP3L
CSF1	KCNS3	TAL2	PSMC3IP	TM4SF1
TPT1	SLC22A10	CIDEB	CHRNA9	FAM82B
NECAB2	NXF1	REPS2	CBLL1	METTL20
TMEM173	RIIAD1	FCN1	CCDC43	QRSL1
C16orf5	PDZD9	CHIT1	UEVLD	UTP11L
C16orf5	RP3-486B10.:	ALG12	HNRNPH3	NME4
SYVN1	BLVRA	SLFNL1	ALX4	MED26
ALG13	HLA-DQA1	SLAMF8	CD163	MRPS17
C9orf131	ENOSF1	MUCL1	TMPO	PRICKLE3
RPS11	IDI2	SGCZ	NUP205	HDAC7
LTBP2	FXYD2	TSPAN7	CHD7	FAM200B
PLCXD3	SPATA20	CYBB	TPH2	C16orf42
PALM2	PRODH	KCND2	SEC61A1	NUDT12
BMP2	ANK2	PAN3	RABEP1	IL4R
HLA-DQA2	ALG5	MEOX2	RASSF10	NMNAT1
ST6GALNAC6	SPINK4	CYP26B1	HCN3	DAB2IP
FBXL3	DCLK2	CDKL1	PTCD2	LMAN2L
LPCAT2	TJP3	PPM1K	AVEN	AJUBA
C4orf52	PBLD	FGD5	PPP3CA	TMEM106B
POMP	SULT1A4	C13orf15	C17orf75	TRIM47
TIMP1	ECSCR	FRMD1	GPBP1L1	NECAP2
RNASEK	ECSCR	SEMA4D	FOXG1	GLTPD1
FMNL1	NRCAM	MFNG	R3HDM1	TMEM88B

PPM1N	TMEM163	TM7SF4	NLK	ARFIP1
AKAP11	WWOX	DIO3.1	KBTBD4	KDELR2
SLC16A7	KPNA7	TAGLN	AFTPH	SPHAR
RPS2	TBC1D8B	GMFG	NOL10	ZCRB1
ZCCHC9	PPYR1	C11orf96	FAM110A	MKNK2
AIF1	THBS4	LRRTM3	TPI1	LCTL
C6orf221	PZP	PPIAL4G	ARL1	RAD23A
TRIM8	TSPAN32	ATP7B	LIG1	PROS1
DPP9	TRAF5	LRRC55	DDX47	PNO1
TRPC3	PPP2R5A	WNT2	KRTAP1-1	GABRE
SLC6A3	TLR2	CITED4	TFAP2B	PPID
PDLIM2	UPK3BL	PROX1	ATP5G2	SPG21
VSTM2L	DIO1	IL12A	DCAF5	CCDC134
UTS2	HNMT	KCNB2	NMUR2	RP11-286N22.8.1
RPL36A	PLA2G4F	GIMAP5	CLN8	CASP8
RP11-343C2.11.1	VIP	SMPD2	ANO7	CTNNBIP1
GLIPR2	AC002472.13	AC007952.1	CADPS2	FEM1C
TRAM1	HIST1H1T	CREB3L3	MDM4	EPB41L1
SLK	AC002365.1	SYT5	WEE1	RAB11FIP5
SH3D19	KLHL6	P2RY10	RMI1	STK3
PTGES	KCNC4	HOXA5	C1orf85	TEF
TP53INP1	VSIG2	ACVRL1	CD276	RAP2C
RPL36	BHMT2	C20orf160	EFR3B	CNGB3
COLEC12	CRISP2	C3	NAIF1	ZNF557
INSL4	ZNF155	POU3F3	RREB1	TOM1L2
RAB31	LRCH4	SIM1	IMMT	FOSL2
ATL3	ACCS	FUT1	GPC5	SDHB
KIAA1704	TLR6	NLRP3	THRAP3	VAMP3
PABPC5	C2orf73	ARHGAP9	43354	BOLA3
PRKG1	CYS1	APOD	GDAP2	BRP44
MRPS28	KANK3	RNASEH2B	PRPS1	PSMB2
NRN1	ATXN7L2	CSMD1	CENPA	CHRNA6
IRS1	SDPR	CTC1	ATP5A1	MTFMT
MFAP4	PTPN2	CLVS1	PROM2	PFDN1
DERL2	AGRP	TNFSF8	ZNF410	ATP5H
RAP1A	ARID3B	PLEKHH2	RSC1A1	ADSS
RPL23	ARSD	MPP1	TRA2B	NTPCR
PIM1	CNKSR1	CTSK	NAA35	NDUFA6
ZBTB16	PAQR6	TLR5	LAMB3	ITPRIPL2
CLIC2	AGAP4	POLR3GL	DUT	YAP1
MMP23B	TMEM241	MCOLN2	SNPH	NCOA2
PNMA2	MCOLN3	IGJ	TRIM22	BRPF3
MLC1	KIAA1377	FAM50B	KIF18A	POLH
CYP27B1	PRB2	SERPINF2	SPRY3	PIGZ
PDE2A	ATP10D	PALM	COBRA1	HSD17B12
SH3BGRL3	DCAF8	LDLRAD2	ANGEL1	MLLT1
ZBTB46	LPXN	KCNA5	ZNF821	MTCP1NB
GALNTL2	BCAS3	CLSTN3	KANK1	TEX261
IER3IP1	CPVL	CERKL	SNX19	FRMD4A
EGFL8	RBM33	ZG16B	GBX1	GAS2L1

GPR34	SLC6A7	RERG	PIAS4	SATB2	
CELF2	CTSH	GMDS	DDX20	OGFRL1	
DPYSL2	SIGLEC1	MMP9	KCP	RAB43	
CCDC165	ABAT	CDKL4	GYLTL1B	TNFRSF1A	
C8orf45	INTS12	MAPRE2	KCNA4	ZNF283	
MSMO1	TTR	IZUMO2	HEATR2	TXNDC2	
RPL10	BIRC7	RALGAPA1	TMSB15B	S100A11	
ROBO4	SH3YL1	P2RY13	EMX1	ELF4	
ZCCHC7	OFD1	SLC9B2	SCIN	KRT85	
PTGER3	CEP192	KIR2DL1	PIP5K1A	CNOT4	
POLR1D	MUC15	RHOH	CHRNA5	FNDC8	
VAPA	ELANE	LRRC2	TARDBP	CCDC58	
TTLL11	KIF19	AGAP3	CNOT3	FKBP14	
BET1	SLC39A8	FMO1	RPN1	MUL1	
RP11-691N7.6.1	CNGB1	RGS9	RAB22A	GDPD4	
RFXAP	HSD17B4	CPZ	NSDHL	CTRB2	
SEMA6D	HTRA4	GLCCI1	HOXD3	MRPL3	
MRGPRF	MAN2C1	VPS36	AP2A2	BAG3	
RRM2B	NUDT9	ANGPTL1	CYP3A7	NR1D1	
UXT	USO1	CCL11	TRRAP	TMEM201	
FAM167A	RP3-412A9.1:	IL17D	ANGPTL7	LRRC8E	
MAP3K14	C12orf10	ATP11C	RRP8	FOXK1	
KCTD12	LRRC43	PODN	TET3	KIF1B	
ACTBL2	TMC6	VIPR2	FAM82A2	C3orf38	
TRIP10	NUCB2	KCNH8	FDPS	PDCD7	
AGPAT3	RPGRIP1	CACNB2	SLFN12	SHISA5	
AGPAT3	FAM183B	PRKCB	GSK3A	TMEM186	
CHST15	RPL4	INHA	NPSR1	MIDN	
MT1P2.1	SAMD14	NUAK2	TMEM187	PPARD	
RTN2	FASTKD1	TNFSF12	OR2T33	UBE2W	
LCP1	PKD2	NKD2	ABI2	PIGO	
SLC20A2	DAAM2	IHH	ARID1B		43162
NDUFA11	FAM82A1	TBX2	DMRT3	PLEKHM2	
KANK2	CCDC87	LOXL4	BMP1	TRPC4	
RP11-330H6.5.1	SHF	CCDC48	USF1	BCAR1	
ITGB3	MCOLN2	PAPLN	H1FX	GNG12	
RRAS	VCX2	SNAP25	POU2F1	MRPL30	
JAK3	KIAA0040	ZFP42	GCNT1	C7orf70	
MPP4	EMILIN1	MIXL1	ACTR2	MRPS11	
CNRIP1	MYO7A	BTNL2	SLC12A4	RNF13	
EPAS1	APBA1	PCSK1N	GABPB1	SMAGP	
SYNJ1	RP11-645C24	KPRP	FAM122B	FAM168B	
CAPNS1	HMCN2	IGFBP2	TNRC6C	KPNA6	
LYL1	HMCN2	CXCR6	COG1	EFS	
C8orf40	HMCN2	AGPAT2	PDK3	SRD5A3	
ABLIM3	HMCN2	TDRD3	PROSER1	FOXJ2	
ZMYM6NB	ARHGAP8	NDP	KIAA0141	ARHGEF35	
EFEMP2	CCDC149	RDH12	HRAS	CIB3	
MSRA	FAM65C	KRTAP4-8	OR52I2	ZC3H8	
MSRA	RHBG	PTK2B	NFRKB	CPSF7	

SGPP1	EGFL7	UNKL	APOL4	PEX13
GLIS2	BLM	CD27	RP11-526J3.3.1	NXT2
PTAR1	WFDC10B	REM1	CDC73	QTRTD1
SCARF2	CACNB2	GIMAP8	43346	CCDC168
CCDC36	RAMP3	IL13	SP2	PDCD10
HRSP12	FAM47E	GFI1	CGGBP1	SLC6A8
PNMT	UBOX5	ELAVL3	FAM64A	TEAD3
GIN1	FGD3	TRANK1	FAM72D	MAP2K1
PYCARD	ARHGEF15	USH1C	DCAF12L2	TIMM8A
C8orf59	C9orf174	RP11-1286E23.18.	MPP6	RAB3D
AL928654.7.1	FBXL15	GALR1	AP2B1	NME2
C10orf125	BDH2	ZEB2	PDS5B	HDGF
CD209	ELMO1	LIPC	CDAN1	NACC1
CLIP3	ABCA10	PRUNE2	TRIM26	CLCN1
LILRB5	GCM1	EPHA5	MXD3	TBC1D22A
KDELR1	FREM2	NRIP2	MTMR8	HN1L
MEFV	CABYR	OR9A2	OR8S1	UBXN2A
H6PD	OSCAR	TRPM8	SPC25	SUMO3
TGFB2	MUC5B	KIAA0748	MED27	CDKN1A
PMP22	RNASE13	MLC1	C17orf59	MMACHC
RPS13	KIAA1456.1	CELF6	ARPC1A	ATXN7
PTPRE	POLG	LXN	CSNK1G1	RAB7A
PURA	FUT1	FAM169B	PDRG1	BTBD1
MMP2	AKAP13	RPGRIP1	GCC1	PAPOLG
RASIP1	KIF12	GK	HTR4	RIOK2
USMG5	NOD1	LSAMP	RPUSD4	C1orf122
IGFBP4	AZU1	C1S	SAT2	LPAR2
CCL2	SNCAIP	CYP2A7	RBM18	NOL12
MAPK11	SAA2	RGL4	CLDN2	TOB2
USP12	CYP2C8	NBL1	DDX19A	ICT1
DOK5	TUBB1	RFX4	KDM4A	FAM83G
CLN5	LRRCC1	NPM2	IDH3A	NDUFB4
IMPA2	PCSK6	STAMBPL1	NOA1	NDE1
CA5B	CAMK1D	PARM1	SERPINH1	MAPKBP1
FBXO47	ACTG2	CD180	UBAP2L	RAB11B
RPS29	TCIRG1	CYSLTR2	ZYG11B	PABPC1
C16orf74	GAS2	AIRE	AWAT2	OSBP
CECR6	SYTL2	ADAMDEC1	PSMD14	ACAN
GABARAP	SAA2-SAA4	PLA2G2D	APOA1BP	RAC1
MFRP	CLEC3B	TNFRSF17	ZNF408	MAT2A
MS4A4A	GPR20	EVX2	MPDZ	TOB1
FAT4	MYO7B	FOXL1	MEX3B	PSMD12
C14orf1	ACSM5	STMN4	IL1RAP	HIF1AN
CLEC11A	CINP	LRRC9	IL1RAP	ZFR
IGSF21	CINP	LMOD1	H2BFM	RNF7
XCL2	CCNT2	ASGR2	MARS2	BRD2
PTPRG	TEKT3	PLA2G5	SETD3	SUMO2
ABHD13	MYH15	SOX14	CNTNAP2	EPS15L1
RPL30	ARRDC2	TXK	RPAP3	GTPBP8
SLC16A2	PHKB	GALNTL5	XPO7	CCNK

CACNB4	MRPL1	PPT1	ING2	CCDC120
OR52N4	CDHR3	GLT8D2	ATG4D	ULBP2
RP11-728F11.6.1	GSTP1	CLEC6A	USP35	AMZ2
NEK7	RIPK3	SKOR1	MORC4	PHACTR4
BEGAIN	FOLR2	KLRF1	BDP1	SCAF4
EPHA2	NLRP3	YPEL3	BAZ1A	MRPL4
IGFBP6	MAT1A	SOCS1	ZNF445	POLDIP3
C1orf198	STAC3	CARD8	ELF2	MED14
FSTL3	APOD	AC006276.7.1	ENOPH1	ATXN1L
GJA9	FAM22E	MYH8	SENP1	WBP2
TGFB1I1	ANKRD18B	TSGA10IP	POGK	CNNM4
VASN	RNF113A	BLNK	B4GALT3	CRCP
DIMT1	PARD3B	KCNA1	B3GALT4	C1orf212
PRKCB	ATP8A1	TESC	CLASP1	TADA2B
RAB12	OSR1	PGM5	NCSTN	TBC1D13
NR5A2	MTRNR2L3	DLGAP2	YY1AP1	YY1
FAM155A	CIDEB	FAM9B	RP11-330L19.5.	SNIP1
DPF3	C21orf2	SH2D3C	DSN1	TMEM41A
RAB37	PSD4	ZNF683	A4GNT	LDLR
SH3RF3	TXLNB	C12orf68	STAG1	POLR3H
CAMLG	MIA2	LAMB1	CCDC110	HEBP2
COMMD6	SYTL1	CDK5R2	USP46	PHF13
NFIX	DHRS12	RGS21	EHMT2	TAX1BP3
LINC00493	C20orf132	RASGRP2	LARGE	ABHD5
C14orf178	RP11-1000B6	GAB3	UGT2B10	SRPK1
FAM159A	SFI1	COL22A1	TRIM40	ADAM17
RP1-164F3.9.1	ZFP3	CDX1	SCLT1	ORC2
SOCS2	FAM180B	OLFML1	MDGA2	SHMT1
DNM1	JSRP1	KBTBD7	GALNT6	TRA2B
MPO	OVGP1	HOXA2	KLHL12	PIAS4
PRDM8	PHACTR3	HCST	AC092835.2.1	PIP5K1A
OSMR	ABHD14A	TRIM72	WIPI2	USP46
CYTH3	HMGCLL1	ARL5C	ARL15	FBLIM1
SPSB1	ERCC5	PTPN7	FBXO10	TWISTNB
RWDD1	PANX2	LRRC32	FBLIM1	HSD17B1
ZC3H12D	ITFG2	CST1	CCDC155	APEX2
FOPNL	CROCC	HLA-DOB	POU6F2	TSC22D2
NEFM	TMEM114	GPSM3	GSC	MAPK14
FRMPD4	GRAMD1C	ZNF540	ASCC3	PLSCR1
SUSD3	USH1C	GPR142	ZBTB33	H3F3B
HRH4	CIRBP	ERN2	CCDC129	PAK1
CD248	ACY3	SBK2	HMGCS1	TCEB3
PHYHIP	PDE8B	POU2F2	SIPA1L1	COMMD2
RPS16	XKR8	SFRP1	UBE3C	ZNF121
MYL9	ASB4	FCRL3	TCEB3B	ZDHHC5
REG1A	TSPAN7	SLC6A7	ZNF221	AP1B1
TMEM200B	SP140L	UBOX5	DDI1	SRRM1
F3	MYOZ2	MEP1B	CCDC158	FBXO3
FAM101B	SLC6A12	SLC25A45	EHF	
CIDEA	CTC1	TMEM204	UNC119	

RARRES1	MAD1L1	CLEC12A	PTX3
AP1S2	CC2D1A	PRKCQ	ALYREF
HHEX	ITIH1	OR2L2	FZD9
CD300LD	MS4A8B	ATP10D	WDFY3
BCL9L	NOTCH4	BHLHE22	HAS1
HIC1	C19orf71	CACNG3	DIEXF
AKT3	RP3-402G11.[illegible]	REG4	FANCA
C8orf58	ZNF76	TAC3	ABCF1
FAM89A	ADAMTSL2	GNRH2	CLTC
KIRREL	LRIG2	SORBS1	TWISTNB
TMEM45A	THADA	CRYGS	ZNF217
PDPN	CC2D2B	NTRK3	LRIG3
SLC38A9	TFB1M	C20orf132	GALK2
TMEM236	SEC16B.1	SLC17A7	PLS3
AFF3	KIAA0513	AC092165.4.1	CHRNB2
RPS25	SKOR1	SEC31B	ASH1L
RP11-293I14.2.1	TESC	DISP2	LRRC37B
EDEM1	ATP6V0A1	PLA1A	POLE
RNF125	C20orf160	COL16A1	PM20D1
STK24	GLB1L	SPATA13	KCNQ1
SPCS2	TRAPPC12	TRIM13	ASRGL1
CTH	HERC5	BEND4	CA6
DGAT2L6	GRID2IP	MAPK4	KRTAP4-5
LRRC8C	TUBGCP6	CDH7	SMC4
PPP3CB	KBTBD7	MAP4K1	ZNF92
BCL2A1	SULT1A3	PTPN6	OMP
STK17A	CLDN15	CTSS	WDR20
IFITM3	EPM2A	TRAF3IP3	MAN2A1
TMEM14C	ZNF683	GNG7	LEMD2
CCL13	LGI4	KCNS2	PLCG1
FNDC4	RP11-295P9.[illegible]	ST6GAL1	DDX54
UQCRB	P2RX1	CHGB	AKAP1
FXYD6	NRIP2	TMEM196	SLC17A1
SLITRK5	SSR4	BFSP2	DCUN1D2
TFAP4	TCEANC	ZCCHC5	STARD3NL
OSTM1	TSPO2	FKBP11	FAM71C
EFEMP1	GPR111	GABBR2	ZNF645
MXRA8	RASGEF1B	TTN	B3GAT2
RPL10L	MEGF6	FAIM2	CASR
COL16A1	CRYBA4	RERGL	KIDINS220
CYP26B1	43168	SLC25A21	ZNF695
GPR180	GPR162	SPOCK2	ARMC8
LEPREL2	C1QTNF7	CSRP1	TRIM34
KIR3DX1	CXCL17	CLDN5	OTUD5
SIGMAR1	COL4A3	RP11-176H8.1.1	C7orf26
HVCN1	HEXDC	PRDM6	TMEM2
LAYN	AC006276.7.[illegible]	ASB15	PRMT6
CCR1	IFT140	LRRC7	CHID1
CDK17	SLC4A8	43349	CNDP1
SMOC2	NENF	C8orf84	GSS

EIF4EBP2	TMEM59L	GIMAP1	SPIN3
PDE3B	ZNF837	HRH3	MIP
PDE3B	CLK1	ADM2	ATP2A3
USP53	FAM5C	SLC39A5	OR10AD1
ANKRD29	ZNF446	TMEM47	SAC3D1
B3GNT9	C17orf47	OR4C3	TNIP1
FGFR1OP2	HMBOX1	GFRA4	EIF2B5
F8	HHATL	DPEP2	PNPLA5
THAP1	GEMIN8	FIBIN	UBXN2B
BCAT1	NFE2	FRMD3	IFI27L2
SRPX	SMPDL3B	ABCA5	DNAJC19
LRRC4C	RGPD5	KLK12	RAC3
SYDE1	SLC47A1	FGF16	SMARCB1
NHP2L1	MAPK10	PLCB2	MYO1A
RNF5	ZBTB48	CEACAM21	MCM3AP
AOX1	BIN2	BARHL2	MADD
DSEL	YPEL3	ABI3BP	IGSF9
TWSG1	NR0B2	KLHDC1	LARP4
IQCJ-SCHIP1	WDR81	PHOX2B	DOCK7
CHRDL1	C4orf44	PLSCR5	HSD17B1
TCF7L2	C1orf141	FBLN1	ZXDA
CHCHD10	LDHD	LAMA2	EPHB2
CD109	TPSG1	ANP32D	SLC2A14
TM6SF2	TXNDC3	CEACAM4	SCN1A
HBB	ELN	C4orf50	FOXD2
TMEM50A	APOB	OSBPL1A	FAM104A
EMP3	ERN2	MNX1	MRPL12
FAM20C	XAF1	PHF21B	RAB24
PCOLCE	YIPF1	GRAP2	RHBDL2
RSPO4	IFI30	RLBP1	GPAM
NFE2L3	CASP1	CAP1	STRN4
GZMA	AMIGO2	NKX1-1	ZNF687
MARVELD1	PPP1R3D	AKAP4	LGMN
ECSIT	STX16-NPEPL	STRA6	CDC5L
ZEB1	MAPK13	C5orf58	CASC5
ALOX5AP	AASS	RIMS3	ALG10B
ANXA5	BTN3A1	CLLU1OS	MGAT1
MRGPRX2	C14orf2	RTBDN	FAM134C
TIMP2	PEX11G	RLF	ADH5
TMEM11	COL18A1	ADRA2A	TBL2
MOB3A	ANK3	GPR25	PWP2
EFCAB9	F10	FAM155A	APEX2
UBLCP1	KCNE3	BPIFA1	ALAS1
PLP2	FAM184A	CYP2A13	IRGQ
SPOCK1	PIWIL4	IL17REL	CECR5
RPL26	TCN2	GSTM5	ZNF718
C19orf12	DNAJB13	CALCB	PDIA3
FAM40B	SLC5A4	PRR20D	ZFAND6
FAM70B	TLE6	DIO2	C3orf52
LMNA	ASB5	CYGB	ATP6V1B1

MSRB3	FAM116B	SLAMF7	DCTN5
ERVMER34-1	SGSM3	NPPB	MFN2
PAG1	NCKAP1L	POU4F2	TLE3
CTBS	LRRC39	CPEB4	GIT1
ITGA4	VIPR1	COL4A4	AGFG1
PPP1R3F	NSUN7	DPT	PHC1
NUDCD2	TRADD	DCLK2	NDC80
DAD1	CD101	NCF1	CAMK2B
IL4R	RAB20	SELS.1	TMTC4
VAT1L	TRABD	GNG8	EBP
SLC7A1	SMARCD3	KCNH6	AZIN1
GYPE	LRRN3	ESR2	CCDC99
SIGLEC15	C19orf45	EVI2B	ADCYAP1R1
TMEM169	TNIP3	CYTIP	C14orf180
DAB2	CTPS2	ZBP1	TSC22D2
RARRES2	ARHGAP25	PBOV1	ACSL3
GFRA1	GGA1	TDRD6	SIRT3
UBXN8	UNKL	PHACTR1	LRP3
ZCCHC24	OR51B4	NCKAP1L	FAM13A
CAMK2N1	CALCOCO1	FNDC3A	MRPS23
ANKRD60	RP11-505K9.4	RELN	SPDYE2
DNAJC25-GNG10	FMN2	MYOM2	CCDC50
SEC61G	ABCA9	RET	GLYATL1
MSN	DPH5	CD37	TRAK1
43353	EML3	OLIG3	AQP12B
KATNAL1	FBXO40	BAALC	TNRC6B
DEGS1	TSPYL6	C20orf196	LCLAT1
PTRF	OR2L13	KLK14	TMEM177
GSR	ATPAF2	KCNN3	RNF2
MXRA7	CDH26	SPESP1	CDH24
IFITM2	NPIPL3	KCNIP1	THTPA
MBLAC2	CDX1	RP1-310O13.12.1	TTBK2
TM9SF2	STAT4	ZNF643	HIP1R
KLHL29	PLEKHH2	PIM2	SLC37A1
CREB3L1	FLT3LG	NNAT	FAM100B
EDIL3	FGD2	PRICKLE2	ZDHHC17
SPON2	GRID1	MAOB	CLOCK
EHD2	AMICA1	KCNC2	ZNF77
CSF2RB	LIMS2	BPIFB2	PHKA1
CST7	CAPN3	CGA	ARFGAP2
TRIM47	NAGLU	SALL3	WDR53
ACCN4	ZG16B	RNASE4.1	TBPL2
GATA3	ALG9	POU2AF1	GPC4
NDUFB6	UFC1	SEZ6	EPN3
NAA20	SYNE1	CYTH4	EIF4E
RPL27A	BAI3	IL3RA	GPRIN1
CERS1	KCNRG	IGFL2	ENPEP
PROCR	TLR3	CTPS	CLDN12
DIO2	FAM22B	MEF2B	CELA3B
MRPL49	FCGRT	WISP1	CHMP3

CYBRD1	OXER1	FOXP3	TRIM48
CCDC73	CDRT4	KLK9	KLC1
RNF139	DMXL2	IL2RG	RABL3
ADAM9	USHBP1	TFF1	DNAJC22
CXCL5	UBAP1L	KCNK10	POLR2H
GPR171	PROC	GLI1	MLXIP
NTAN1	VMO1	ITIH3	ZNF167
CRTAP	FOXL1	CCDC54	SOX6
ZBTB4	ALDH5A1	SERPINA10	TNIP2
POU2F2	ZNF547	DENND1C	KIAA0232
TEX264	FOXA3	GYPA	CSNK1A1L
KCNK3	LARP7	TFAP2D	TRAIP
SEC61B	PLEKHB1	KIR3DL1	TMEM104
PLCD3	RTDR1	FOLR2	C19orf40
C12orf53	SOCS1	EPB42	COG8
AC100808.7.1	C19orf60	NEUROD2	CTSC
RPL22L1	HAAO	MTMR7	DBR1
HSPB2.1	HBE1	C8orf80	NLN
SIGLEC14	TATDN3	DNASE2B	POU4F1
TREML2	HK3	FGF18	TRAF4
LRRN4CL	DENND3	RP11-77K12.1.1	GALNT14
STK17B	WDTC1	C1orf95	HIATL2
IGF2	ADCY4	CASP10	CDON
METTL4	PTPN6	PTBP2	NFYB
GPR176	MCF2	43168	NARF
ARFIP1	SEC31A	LRFN5	SCGB2B2
COL5A1	HSD17B8	ALG5	ATAT1
STARD6	GALT	ITPKB	MED12
OR5B2	THAP9	KIF19	ZNF292
SCN3B	C4orf33	C9orf95	OXCT1
NECAB1	C17orf62	USP17L1P	RTN3
GP1BA	KCNJ16	SLC26A4	CREB5
FAM180A	FGR	ZMPSTE24	GCA
KDELR2	ZNF540	SFRP5	ZNF287
XPO4	CELF6	ABRA	EHHADH
SLC35F1	TTC23	C3orf77	PRPF40A
FBXL7	LACC1	GAGE12G	SPDYE6
PRCD	RNPEPL1	NMUR1	IGFBP2
PROKR2	FERMT3	LCN8	DCUN1D1
FGF2	KIAA0408	FGF17	PRKRA
HRH2	ABCB1	GAGE12J	UHRF1BP1
REEP3	ASMT	CTAG2	RNF14
SLC16A10	SEC31B	PHGR1	TAS1R2
MKNK2	UNCX	DFFB	OAS1
ZNF729	OPALIN	C10orf54	PFDN2
DIRAS1	KCNMB3	NRXN3	ZNF286A
RPL27	LRRC49	GAGE12C	IGF1R
RAC2	PLA2G6	RP11-1286E23.4.1	CYB5RL
FSCN1	FATE1	PTPRN	PSMD1
RER1	DENND2D	HTR1A	C7orf53

CAV2	DNASE2B	NEUROD4	TFDP3
PRRX2	OR1N1	COLEC10	TRPV6
BTBD19	PLA2G12B	UGT3A1	BMP5
HSBP1L1	MAP1A	KIF21B	TAF11
ARPC3	MTIF3	GAGE12H	ZNF195
NOG	KIAA1755	GAGE12E	SYK
SHC1	IL29	POMC	KLF3
HSCB	PLA2G12A	SLC7A14	WDR36
NDUFAF2	KIF21B	KIAA0040	SMARCA1
LY96	TRPV2	DSCAM	STRBP
C17orf39	CCDC48	AGBL1	EIF4H
MSC	NUDT14	HMP19.1	KCNE4
MDFIC	PION	L1TD1	C7orf49
TRIML2	ASTN2	PNMT	SHROOM2
PLEKHA4	MRPS31	TRIM49L1	SS18L1
FSTL1	FCGR2B	GAGE12D	TSSK1B
GNG2	VWA5A	GP2	CKS1B
GAS1	MNX1	CTC-554D6.1.1	GSDMA
PROS1	H1FNT	OR2M4	PIK3C3
HTRA1	BCAN	TMCO2	DIP2B
BPIFB4	RCN3	OR2Z1	GNL3
ZYX	LAMB1	SPAG11B	NEIL2
HECW1	TNFRSF18	DEFB121	C17orf70
NUFIP1	ABCA6	GIF	HNRNPA0
PAPD4	TNFRSF25	BARHL1	TDP2
PPM1F	KIAA1024	MTNR1B	AC005280.1
GNAL	ITPA	HIST1H2BA	IBSP
GUCA1C	PCSK7	AC013269.5.1	C11orf89
RPLP2	CHRNA3	OR51G1	RP11-1286E23.10.1
CYR61	CD81	GJA8	VBP1
DCUN1D3	C3	TNP1	SLC30A3
GXYLT2	THAP6	RESP18	PRMT1
SPAG11A	HSD17B7	RP11-234B24.6.1	FANCM
C9orf21	RGN	AC010872.2.1	PRDM10
PRDM13	TARSL2	ZNRF4	TAF4
STYX	SERPINF2	OR52N5	PLEKHA7
RUNX3	ZNF182	GAGE12B	LRRC8B
C9orf125	PDZRN4	GPR139	PPP2R3A
ISCA1	PAMR1	FAM47A	DDX6
SNAPC2	CHN2	MFRP.1	ASXL1
STMN3	AGAP3	GPR119	POLD3
CNTFR	ATG16L2	CST9	REEP4
SIRPA	MATK	CARTPT	SUPT6H
GLP2R	TCEB3C	KRTAP9-9	CNTNAP4
SREBF1	CLCNKB	TAS2R1	DIAPH1
PHOX2A	GK	IL22	CTDP1
PPP1R18	RBM20	GC	NUP62CL
FUT5	EPB41	COX7B2	SMYD4
SERINC1	IL11RA	NLRP13	H2AFY2
STAT5A	BLOC1S1	RP11-548K23.11.1	FAM122C

MFAP2	C11orf96	DYTN	C5orf25
TWIST2	AXIN2	PTH2	OAS3
CCK	ALPK1	GIMAP4	RASAL1
HAPLN4	DAK	NEUROD6	SERF1B
TBC1D4	IPO8	NEUROD1	PPAT
THAP5	MCF2L	GAGE1	NPTN
ANGPTL3	DUSP26	SLC14A2	ARMC7
USP38	UNC93B1	CALB1	ATP11B
SNX18	TMEM180	TMEM74	NCBP1
ALG2	CDC42EP5	GLI2	EIF4A3
HS3ST3B1	CDK5	RETNLB	RORA
BEND6	KIAA0564	RNASE11	ZSWIM1
SLC35F2	LRRK1	IRF4	AADAT
LIN28A	TRIM39-RPP2	PRLHR	DAZAP2
ZNF99	MAP6	GGT5	ANKFY1
ANKRD13A	CHPF2	TRIT1	YES1
MB21D2	RABGAP1L	ABCA10	DNAJA1
CLMP	FASTK	RNF128	GOLGA6L4
FHL3	L1TD1	ALDH1A1	PTPRA
QRFPR	CPT1B	CALCA	ISY1
TNFAIP8	UACA	KIR3DL2	RIT1
PROK2	TRANK1	FAAH2	PGK1
ADAM19	DTX1	CFC1B	GPR37L1
GFOD1	SLFNL1	UMOD	ACTR3
GFOD1	AP4B1	INSM2	POGZ
IRX4	BPIFA1	PAH	PODXL2
KLK1	LILRB3	LIMS2	POLR3K
BCL2L2-PABPN1	CHIT1	SST	ING5
GFAP	OCM	FGL1	MAPK14
C16orf52	GIPR	GAGE12F	SGPL1
RFTN1	FAM214A	GAGE2A	ZFAND2B
SLC25A32	TMEM134	EMILIN1	CHAF1A
LATS2	NFXL1	MMP3	MTX2
IGFBP3	TLR5	INSM1	ADA
CCL8	PRICKLE2	MYCL1	TRIM52
BSX	BPIFB2	PPP1R17	ZNF777
RAB5A	NEK3	AC217771.1	NPC1
LRRC70	RP11-762I7.5	AXIN2	DHCR7
NXPH2	CAT	NEUROG3	C19orf48
THBD	STARD10	C5orf47	PEX2
BICC1	NLRP14	SLC38A11	MPRIP
RAP2A	TREML1	LCN15	LSM2
ANAPC10	ZNF18	RFX6	VOPP1
C5orf24	SMPD3	BAI1	RPL39L
PHC2	ZDHHC1	CFC1	ARFIP2
RALB	TNNI2	ASCL1	ACTL6B
TTC28	SORBS1	KRTAP1-3	C22orf28
AP005814.1.1	PRAM1	CDH17	DYRK1A
TMEM22	NAA16	SSPN	GS1-211B7.1.1
TTC39C	ARHGEF10L	OIT3	CDC45

AL034548.1	INPP5B	KCTD4	OTX1
GPR150	WISP2	FCRL5	ZNF148
SKI	CRYGS	NDST4	ZACN
DDA1	IL12B	PGLYRP2	HERC1
FOXN2	VPS33B	LY9	DEK
COL6A1	CCDC88B	NOL4	RPTOR
PPT2	FUCA1	ARHGEF25	NOTCH2NL
WASF3	SLC46A3	NEURL	HAUS1
GPR32	GUCY2D	MCF2L	CNPY2
CAPZB	GNG8	GADL1	INPP4A
ADAMTSL5	SIDT2	MYBPC3	SYT2
IPMK	TBC1D10C	ATP8A1	VEZF1
RP11-122A3.2.1	MERTK	PCSK1	MKRN2-AS1
HEPACAM	NEIL1	PDE1A	HSP90AB1
VMA21	NRL	FAM123C	RNF4
BCL3	LUC7L	PPEF2	NEDD4L
C10orf90	TSSK3	IRF5	CLSPN
KCTD11	TBCK	BRCA2	METAP2
PPP2CB	ARHGAP15	SCGN	PLEKHJ1
IGDCC4	ARHGAP15	ZNF642	TP53I11
GLIPR1	MLKL	ASXL3	ZFYVE21
INSL3	C5orf38	LDHAL6B	PHLPP1
RHOF	PPIB	GABRB2	DDX42
EBF3	PARVG	CER1	UMPS
FGFR1	RP11-632K20	LACC1	SAP130
RPS23	SURF1	ZIC3	MTERFD2
ADAM12	TECTA	SCN7A	MESDC2
XKR6	EPHX2	LIG4	GALNT2
TMEM240	FGF9	CPLX2	STIL
GHITM	ZNF710	PCP4	TUBA1C
SLC25A43	ADM2	SLC8A3	PQLC1
IKZF1	SETDB2	RTN1	DGCR8
SNX3	ZNF784	TNFRSF13B	ADAR
VASP	IL3RA	SYT4	SLC5A6
PIP4K2A	PPIAL4D	FCRLA	SNF8
RNASE3	RIMBP2	CCDC69	IPO7
NPW	ASNS	CD40LG	CACYBP
RASA3	CES1	LMX1B	PROKR1
C19orf43	FLT3	RTL1	TRAM1L1
PCDHB15	LIAS	DTX1	ADPGK
QKI	WDR86	ANKRD6	KRR1
XKR3	RASSF4	PVALB	PHEX
PLAU	GMPR	TMC8	ZC3H11A
GOLGA7	CREB3L3	PLCG2	UGT2B28
OSGIN2	TDRD3	GRP	C20orf144
PNLIP	PDE4DIP	HS6ST3	C2orf47
C20orf30	PMEL	IGFL4	ANKRD37
LSM3	PLEKHO1	NR0B1	MAPRE1
TOMM5	SPATC1	SEZ6L	CMYA5
HMGA2	CCDC54	CST2	SH3RF1

UBE2E2	GUF1	RIMBP2	USP30
VKORC1L1	LUZP2	STMN2	COX4NB
THOC6	CLDN5	ST18	SMARCAL1
ST6GALNAC4	LAMA4	KCNT2	RQCD1
C3orf14	CAMKMT	ERO1LB	KRTAP2-3
N4BP2L1	ACTA2	BDKRB1	NAB1
FAM172A	USPL1	RGS7	KDM5C
PID1	VAV1	KCNK9	ZKSCAN5
IMPAD1	SLITRK2	CABLES1	MRPS12
TMEM158	ARHGAP4	DCC	GYG1
CBS	ETV7	GYPC	NRIP1
PDK2	COL4A4	CALY	ZSCAN29
ZNF385D	SIGIRR	CRLF1	MID1
PXK	DEF6	IL16	DPY19L2
NLRP6	CSMD1	RASL12	HMG20A
POLB	LEAP2	SSTR5	NCOA6
GNG5	COQ4	SLC18A1	ZNF454
KCNK2	ZNF235	CNGA3	RANBP6
RAB11FIP5	CHAC1	LRFN2	MFSD6
U2AF1L4	ERAP2	ABAT	PCTP
HAS2	DENND1B	AMIGO2	INADL
NUDT4	ZNF554	KCNU1	DCHS2
RNF185	SLC25A45	ETV7	CCDC150
MYOM3	TBXAS1	NKX2-4	STARD7
COX16	VARS2	DEM1	HIST1H1E
KLF13	SNAI3	CHAC1	KDM5B
PRRG1	ALG12	PRELP	C20orf151
TFPI2	ZMYND15	PTPRCAP	TFAP2E
ZBTB7A	TNFRSF14	C12orf26	ZNF263
TEF	SDF2L1	BHLHA15	TTYH3
LITAF	PLA1A	THBS4	PLSCR1
MPV17L2	CADM1	SHOX	SAFB
43167	METTL18	SLC46A3	ANKRD13B
FAM211A	BBS4	ASB5	CDC42BPB
FHOD3	ITGAX	MRPS31	TMEM81
CCBE1	BDKRB1	TRPV2	TMEM5
GCK	IRF5	SCG2	C19orf52
FAM105A	TRAPPC6A	CPXM2	RP11-697E2.6.1
TMEM64	CXorf21	CABP7	DEPDC1
LRRTM1	WDFY4	CNKSR3	NAT10
UCHL3	CYBA	ZNF684	KDM1B
GJD4	KIAA1324L	SMAD9	ERVW-1
LEPROT	PNPLA7	DCX	OR51E2
AVPR1A	JMJD5	PPP4R4	NOTCH2
TMED5	FAM18A	MUSK	ETV6
TMEM43	TRAPPC9	MGAT4C	UNK
CNGB3	F5	SNCAIP	MEN1
EIF1AX	RINL	SCML4	FBXO9
MZT1	CNTN6	TNIK	CTBP1
MBD2	NMUR1	MORC1	DNAJC5G

ATG10	MAN2B1	SDPR	NFIA
POLR2K	ZNF451	PCSK5	LRCH3
PTPLA	CRELD2	OLIG2	GABRA3
DUSP7	MRVI1	SLC38A8	ADRA2C
SP7	CTSS	PKD2	LHX2
VSTM4	NLRP1	LAMA4	PTCD1
GNG10	TMEM88	CAMK4	RRAGB
ETS1	C22orf32	CACNA1S	FRMD8
SGTB	LIG4	FGD3	FAM187B
BCKDHA	CASP10	CSF2RA	EIF3J
CADM3	PLD3	ZIM2	PREP
FAM214B	C6orf108	ELMO1	TP53BP1
RIPPLY2	CABLES1	CASD1	ZNF674
DCTPP1	ITPR2	ACTA2	LTK
SNRPB2	TRIM13	DPEP1	SLC6A19
ANKRD44	CXorf57	HMGCLL1	JAG2
IKZF3	HSH2D	LRRN3	GPATCH8
PRICKLE1	PLCG2	KCNK16	SORBS2
RP11-585F1.10.1	ZAP70	TNFRSF19	COASY
DOCK11	CYP4F22	HERPUD1	MST4.1
PRSS33	FAM50B	PROX2	PHF16
VPS26A	PLCB2	ABCA6	PUF60
TPST2	ASB13	CRABP1	C17orf89
UBE2A	RCBTB2	SLAMF1	SLC25A39
OLFML2A	SMPD2	NPAS4	CSNK1E
SH2B3	NCF4	HMCN1	KCNJ11
JPH4	ALDH1A1	RBFOX1	EP400NL
IL34	GZMM	STAT4	TBC1D30
RRAS2	ABCA5	FAM46C	GINS3
GOLPH3	MS4A4E	DHRS7C	TRIM3
C1QL3	LMF1	MS4A4E	H3F3B
SUB1	IFFO1	PDE1B	DHX9
RNF138	PRR5	MFSD2A	MTMR4
NLGN2	PDE1B	TBC1D8B	LSG1
AP3S1	AKAP6	FAM214A	PHB2
VEGFC	SYN3	TPH1	ZC3HAV1L
C5orf28	BTK	TBXAS1	FGB
PLSCR4	C12orf26	KCNA3	TSPAN14
ZNF169	C16orf95.1	CPNE5	VRK2
FEV	S1PR4	FAM113B	C20orf3
ZNF585A	FAAH2	SETBP1	DUSP16
FAM131B	TNIK	FOXF2	OGDH
KIAA1143	ICAM2	PTGDR	ANKRD17
TP53	ZNF775	C22orf32	ZNF275
PAFAH1B2		GIPR	LUZP1
ISCA2		FAM55D	PANX1
CT62		TRAPPC9	ARHGEF19
ZNF264		MYO7B	FER
COL1A1		CELF3	ZNF749
ST3GAL4		TAGLN3	MED13L

THY1	GALNTL6	SLC5A1
ONECUT3	MBP	CALCR
CLPTM1	BRP44L	INSRR
UBE2D1	SETDB2	HTR2B
TAF10	GABRR3	IRF2
KCNK13	ARHGAP25	FAM76A
GAR1	RP11-1280I22.1.1	UPRT
C17orf74	OR51B2	PRDX2
SRGN	TAAR1	TCF19
SLC25A26	CNTN6	PLA2G4B
TMEM192	PDLIM3	CDK2
ELOVL2	PLA2G12B	ZKSCAN3
HRH1	ATP1A4	CLDN6
PDPK1	TRIM39-RPP21	CDH1
CHRAC1	COL25A1	SRRD
FJX1	CCKAR	ANKRD56
GZMK	CLEC3A	ZNF41
PABPC3	NFYC	CDX2
ROR2	KCNA6	SNX4
APOBEC3D	PNOC	C9orf86
ZDHHC8	HSD11B1	SRP68
SLC39A10	LMF1	PCDHA11
S100A2	SMPD3	CREB3L2
UBAC2	SMAP2	HELQ
ETHE1	RINL	FKBP4
CLIC4	LEPR	GLYATL3
B4GALT1	SPATC1	ANLN
RHOA	HEPH	MGAT5B
KLRD1	PNPLA7	TRPC4AP
ZFAND1	DDC	PTPN13
UBXN6	UGT2B4	C9orf7
SEC24A	SERPINF1	ODF3
COL6A2	KLHL6	FAM83D
M6PR	CLEC1B	NAT9
TMEM9B	TMEM59L	TUBA1B
PTP4A2	DOK3	CABLES2
RBMS3	OR7C1	PDE4D
QSOX2	KCNQ4	ATP6AP1
C1orf21	HAND2	EEFSEC
SLC30A5	C8orf47	RPH3A
TGFB1	IPO8	UCHL5
GLS	NRCAM	EPT1
NSA2	FAM189A1	TRIM37
TUBA4A	ELN	TBC1D7
MMP14	ACCN1	BSDC1
C16orf72	KRT40	KIF14
PTF1A	KCNJ3	FAN1
FAM132B	OR2L13	ZBTB9
PPP1R11	ITPR2	SRP14
ZNF527	PDK4	KLHL36

ANKRD40	CD79A	ZNF490
ELFN1	LPXN	CFL1
AKAP2	NLRP14	USP36
GJC1	CACNG5	NR4A1
43160	ITGB7	MMS22L
GJA3	AKAP6	PLD5
ODZ3	CRYBA4	COPG2
STIM2	PDZRN4	COPG2
TGDS	FGD2	ACAA2
NR3C1	BTBD17	KLHL25
ZNF580	CD101	PLCXD1
CPS1	GPR15	CSH2
NUPL1	SEC11C	C1orf109
C1orf49	TMEM119	CLINT1
RPL37A	RASAL3	TAF2
CD3G	NKX6-3	SEPSECS
RECK	TLR6	ARID4A
ARL8B	PDGFRA	NEB
NXN	FCGR2B	MAGEF1
NUDT11	ENTPD1	MUTED
GOLT1B	DERL3	TRERF1
FAM92A1	RCBTB2	CSNK1A1
GNAQ	ZNF775	IMPA1
FAM102A	CNTN4	HNRNPF
SLC15A4	TBX5	RSRC2
SLC38A5	RP3-412A9.11.1	MLL
RAB2A	RAB3C	ZWINT
EPN2	AMPD1	SURF6
TSPAN5	PLEKHO1	RP11-565P22.6.1
C9orf89	FLT3LG	PVRL4
SPRYD7	C16orf95.1	FGD1
CNOT7	LPL	BAIAP2L1.1
FHL2	OCM	ZNF736
ZNF141	ASB13	GNB2
CCNI	SLITRK2	SLC7A5
IL11	KCNJ16	HOXC11
MRPS36	KCNC4	CAPN13
CTDSPL	ANAPC4	ARHGEF6
FAM210A	GNLY	ARNT
WASF2	OSR1	NCAPD3
EHD1	CXorf57	SUDS3
PGRMC2	CLEC10A	ELAC1
CHM	FOXF1	PHLPP2
TTC24	APOB	MYO1G
NDST3	GPR114	TBRG4
ZIC5	DUSP26	NUFIP2
C3orf70	NCF4	SAP30BP
SLC38A2	PMEL	ZNF136
LAPTM4A	P2RX1	PSRC1
WIPF1	KIAA1755	POP4

CCDC117	CD1D	KIF2C
QRICH1	RUNX1T1	ABCA12
PWWP2A	FMO2	FIGNL1
CA1	DENND1B	ANKIB1
YAP1	FGF9	POC1A
SLC23A2	ABCB1	PDDC1
CRIM1	ACAP1	ONECUT2
SLC36A1	TXLNB	PRKG2
CDC42EP2	S1PR4	PRSS46
MNT	SLC24A3	ISOC1
VGLL4	CDC42EP5	CASP6
SBDS	SLC35D3	CGNL1
CALHM2	EPHX2	TEAD1
SNRPD1	ANK2	PAK1
ELK3	ASMT	METTL13
FOXP1	RCBTB1	ASAP2
SEH1L	KCNK17	QDPR
TNFRSF11A	HGF	ZNF193
TSSK2	IFFO1	SLCO5A1
P4HA3	SMCHD1	ILK
SP9	CYP4F22	PSMA7
FBLN2	TSPAN11	PLA2G4A
IL7R	PTPRN2	MED13
HINT1	C7orf58	TFAP2A
COX6C	BIN2	KHK
UBE2R2	LCN10	RABGAP1
ASAP1	MATK	ZSCAN30
LACTB	RAB33A	RALGAPA2
ERBB2IP	ABCA9	NCAPD2
UBE2D3	NLRP1	GPR98
EIF1	ICAM2	AC005726.6.1
SSR3	SYN3	MON1B
NRM	AMICA1	ZAR1L
ANKRD57	F5	SPEG
BTF3	KIAA1024	CCDC97
RAB39A	MRVI1	MALT1
TBC1D20	FLT3	DHPS
CRLF3	GZMM	CHEK1
PCBP3	ARHGAP15	PLCZ1
FAR2	ARHGAP15	ORMDL3
MAF1	WISP2	AP1G1
ZIC2	TBC1D10C	MYO6
NPBWR1	CXorf21	DOT1L
RNF38	BTK	HNRNPU
MAX	HSH2D	NSMAF
GPC6	SSR4	SLC5A12
HEG1	SNAI3	RELT
ZMYM5	NAA16	PAQR4
SEMA7A	ZAP70	ATP2A2
TXNL1	WDFY4	DRP2

EMR2	RASA1
TMEM41B	SH3GLB1
ITPRIP	OR2J3
UBE2E1	FBXO22
IGF2BP2	LAMTOR1
C22orf39	FLOT1
DNAJB5	NDUFS2
GCLM	POU5F2
ELL	FARS2
C10orf122	SNUPN
PSMD8	TBCCD1
ZFAND3	SIVA1
RAB33B	ONECUT1
E2F5	COPS7A
UBE2B	ZNF346
NAF1	USP13
IFFO2	PVRL2
PSMF1	WNT3
TLR9	BDKRB2
RBBP9	SEC14L1
ISCU	HEBP1
PPP6C	ESCO2
FKBP5	CASC3
MAG	TCEB3
GAS2L1	PPM1G
TMED7-TICAM2	TMED9
C7orf73	RIOK1
VGLL3	PSPC1
NDFIP2	PRUNE
NDRG1	ZBTB5
CHCHD4	MLIP
KDSR	ZDHHC18
IKZF5	SERINC5
SURF4	POM121
FAM164A	ERAL1
KLF16	CNDP2
ZFPM2	FAM190A
RNF112	TTLL2
C9orf41	SLC39A9
GLI3	BTN2A2
TM2D2	ZNF117
NCS1	DGKH
FAM122A	HIF3A
MAP7D1	C9orf150
CD44	CSDE1
MAPK7	WWP2
NCOR2	ZNF124
MEF2BNB-MEF2B	SMARCC1
SF3A1	USP42
ZNF706	FASTKD2

NDUFB5	HTATSF1
TMEM19	LRRC31
ZMAT3	C3orf33
RHOBTB3	TROAP
EFHA2	C9orf100
TRIM7	OLIG1
TAF3	DCAF12
ZNF699	SNRNP200
MFSD5	FTSJ3
PCDHGC3	SRSF2
OGFRL1	MAML3
C4orf43	FAM171B
ADO	ADAMTS7
SLC7A7	FBRSL1
RAB43	C1orf131
TLN1	TGFBRAP1
STK10	B4GALT2
ASPHD2	MRFAP1
KIAA1644	MIER3
MCTS1	SLTM
TNFRSF1A	ZNRD1
ANKH	RNF19A
SLC37A2	GPN1
TMEM239	ZCCHC6
ZNF283	TMEM170B
KAT6A	UFSP2
DNAJC18	ELF3
CRX	CYP19A1
GATAD2B	MPZL1
VCPIP1	SBK1
FPGS	SSR2
C6orf57	GTF3C2
MTDH	ZNF786
KCTD17	MYH13
TMEM38B	DDX11
GFPT2	SKAP1
GPR153	EXO1
TRPV3	CARS
ZNF805	KRTAP2-4
CLDND1	UBN1
HIPK3	MAD2L1
PFN1	HNRNPA3
LYSMD3	FAM126B
FAM25E	DSP
RPS27L	RAB19
ELF4	CENPM
CDRT1	RORB
MRP63	XIRP1
TPM4	CIAO1
ARL6IP6	TNPO3

SERPINB8
COTL1
KLF12
LRP12
NOL9
OLFM1
ZDHHC20
FKBP1C
RHOG
SHB
MKKS
RP1-130H16.18.1
PBX3
SPCS1
TMEM55A
DNAJC25
SNX12
CTDNEP1
LRRC38
RAB8B
TRIM35
KLHL33
DAZAP1
DCUN1D5
TMEM194B
GNAI2
NFIB
C22orf13
CIC
KLF9
FNDC8
PINK1
C5orf62
DERL1
ZFC3H1
ACTB
RAB28
PCNP
FAM126A
COPZ2
PPRC1
FSCN3
FBXL20
USP22
C5orf41
RAB21
MTX3
C17orf107
JKAMP
OTUD4
BORA
DBF4
TECPR1
THOP1
H3F3A
SV2C
CAPN5
NPEPPS
C2orf68
RPIA
ILF2
LDOC1L
DMRTC2
HNRNPA2B1
NKRF
ZNF227
AURKA
STX19
RABIF
CHCHD2
FAF2
RP5-1086D14.3.1
ATF7IP
LLPH
TLL2
ATF6
DMBX1
ALKBH4
NSRP1
ATP8B1
STIM1
SCO1
SLC5A9
C10orf113
PPP2R1B
E2F2
COMMD2
TMEM44
CDYL
TTF1
RAE1
DENND5A
FNTB
CDCA4
MTMR1
SLC25A3
DOPEY2
FZR1
MBOAT1
GALNT7

FAM57A
EI24
TPP1
CBL
TRAM2
SLC39A14
MAGOHB
CMC1
LSM12
RASSF8
CMTM6
RP11-463D19.2.1
CHAMP1
PDP1
CLIC1
TCEB1
SGCE
LAMP1
DPYD
TMEM200A
EXT1
SERTAD2
ERH
MUL1
ZDHHC3
DHFR
RPL35A
MRPS18B
ITGAV
LMAN1
PABPC4L
ABLIM2
PHF23
C14orf129
POLK
C13orf33
BAG3
STAM
NR1D1
PITPNB
ST3GAL1
CTDSP2
MMGT1
FAM110B
C1orf144
FKBP1A
CCDC71L
NRIP3
COX7C
FOXK1
TCF3
TMEM194A
GLYCTK
SEMA4B
MYLK2
ABCC8
NIN
ERCC6L
RFC1
SWAP70
TSR2
HSD11B2
PRMT3
ACP2
BECN1
GPATCH4
SCD5
RRP36
FUNDC2
ADAT3
ZP1
C11orf30
KLHL7
AGGF1
COMMD9
PRDM9
CAB39L
VPS18
NUP62
SOCS6
ZNF655
OR56B4
HAUS3
MARK1
RBM46
HYAL2
MELK
C12orf73
NLE1
C11orf84
MTO1
GPR77
LYSMD1
CDKN1C
C15orf23
PLEKHF1
CEP78
GMNN
REST
SNX1

C9orf3
UST
CADPS
CHST2
LYRM4
OLFML3
ZNF395
SAMD4B
CSNK1G3
ACER1
CPE
BNC2
TMEM209
KPNA3
DSE
DGCR2
ABCE1
RGP1
FRMD6
DUSP5
SHISA5
TMEM186
C4orf3
SUFU
SSH1
GFER
RAP1B
GNA11
UBE2G1
ATF7
TMBIM1
LIN7C
IP6K1
OR13J1
WASL
LASP1
PLSCR3
BICD2
FAM49B
MEX3C
LRRC3C
APOBEC3C
CENPB
CCDC80
TMEM39A
TRPA1
C6orf48
SYT14
MAP1B
UBE2I
MRPS7
BET1L
ORMDL2
USP10
HMGN1
EWSR1
HTRA2
THUMPD3
TRAF6
MAPK6
SPRYD4
CTCF
NPLOC4
ZNF121
NRSN1
TMEM183A
ABHD16A
MEST
DBF4B
C15orf52
CCNO
CYP2C19
CA5A
ERMAP
KDM1A
MRPS10
LRRC57
GUCA1B
ZNF215
ZDHHC5
DCAF7
H2AFY
CHD6
TJP2
BEST3
KAT7
RRP15
PRSS22
EIF3F
XAGE3
NMD3
ACTL6A
RAD51AP1
TBP
MMP26
PLK4
FAM171A2
PRKAG3
OTOG
RFC4

C4orf46
NUDT19
GNG12
C12orf66
EPHA6
ABTB2
PHF21A
C12orf23
MED22
PICALM
TSHZ1
TMX2
DPY19L1
GNA12
SRRM3
TIMM22
KLF7
USP14
NDFIP1
AKIP1
SLC25A24
CSNK2A1
ST5
HERPUD2
DR1
ZBTB25
TRA2A
GARNL3
ZDHHC2
PTBP3
CDR1
WIPF2
PANK2
AKT2
NXT1
HM13
CYB5B
C11orf24
KIAA0930
MAF
PJA2
KLF10
BLOC1S3
NANP
STK32B
IL17RA
RP1-239B22.1.1
KIAA0355
ASB14
ACTR10
CPSF4
TSPAN17
CNBD1
STRAP
MARCKSL1
AP1B1
ATP6V0D1
PRR20E
DLST
STIP1
ILF3
FGF12
PDZD4
LBR
BTBD3
C4BPB
ALS2
WDR76
43166
NEK2
PRDM12
CDHR2
GIT2
TTI1
C17orf63
CPSF2
SMOX
EIF1AD
VSTM5
ADAM29
LSM7
CDC27
CNOT6
VAPB
PLEKHA8
DBNL
FNBP1L
SDAD1
ALX1
WDFY1
SNX11
CACNB3
TDG
PUM1
APOA2
NSUN3
ARHGAP10
ZNF154
KSR1
C12orf45

CMTM7
ATP6V0E1
IPO11
TUBB6
PTP4A1
MTPN
ZFP91
GINS1
RAB5C
EFHA1
LAPTM4B
KCNIP3
ZNRF2
PACRGL
SETD7
ZNF260
SLBP
FBXW5
CENPH
HOXD8
NDEL1
GNPDA1
MAPK1
MTMR6
PRNP
SH3PXD2B
SMN2
RAB35
ANP32B
C7orf42
GNAI1
SEC24D
CRKL
PAWR
UBQLN2
EPC2
ELMOD2
GNRHR
SPHK1
PGBD5
SPPL3.1
MAML1
NDST1
NDST1
AMPH
DCAF16
ZNF70
LIN52
MAVS
DCLK1
ARHGEF5
NAV1
STARD3
HOMER1
FRMPD3
MCM10
TMED8
ZNF510
ARF6
WDR45L
JARID2
ZSCAN21
RNF168
RP5-1187M17.10.1
SPINT1
LMBR1L
HIPK1
TMEM235
WDR41
DHX29
TUBA1A
ZCCHC12
MYH2
HUS1
WDR61
WDR61
PIPOX
TERF2
WIPI1
MANBA
LGI2
SSRP1
C1orf105
KRTAP10-10
SPTY2D1
SIN3A
EXOC4
EXOC4
ACBD3
CDC42SE1
UNC93A
ATG9A
DKC1
HDAC8
TRIM33
DHX15
PTGES3
KIAA0226
DOHH
ING1

TCP11L1
FAM83G
ABL1
CDK14
C14orf43
PPIA
STK35
CPOX
KLF11
GORASP2
C18orf25
SMN1
MAST4
AFAP1
PSME3
DEPDC7
A2LD1
LRRC8A
SMG9
DUSP1
FAM19A2
OR5C1
RFC3
ATP6V1G2
PITPNA
PHF12
YTHDF3
ABI1
KCNQ5
ME2
KIAA1967
YEATS4
CACNA2D1
LCORL
TFDP1
RNF144A
INPP1
UBTD2
ATP5SL
UBE2Z
TXNL4A
FXR2
C19orf10
ATP13A3
TPGS2
CDS2
SCAMP1
IGF2BP3
FAM108B1
SDCCAG3
FOXP4
SYT16
POMT2
CEP76
YWHAG
USP9X
RNF180
ZNF330
SKIV2L2
UBQLN3
SDK1
IWS1
DEDD2
SLC6A5
C2orf63
GFM1
VANGL2
ABHD2
CSTF2
FCHO2
TCP10L2
DTNB
LYSMD4
GLOD4
CTC-435M10.3.1
SETD5
UPF3B
POLR2G
MTRNR2L7
CIDEC
CTTNBP2NL
BAZ1B
ADAM22
BPTF
DOK6
FOXN3
ABHD15
ST13
LEO1
ACO2
C6orf47
RTTN
STMN1
WDR7
C12orf34
CLDN20
VIL1
EZH2
TOPBP1
C12orf48

C19orf47
PCBP2
SUN2
C5orf43
CRK
RIN2
ACOT7
ERGIC1
PHC3
RMI1
GSK3A
BMP1
RNF4
B4GALT2
SWAP70
SMAD4
CHTF8
LRFN3
ATF6B

RAD54B
TDRD1
HNRNPL
PCLO
PPP1R14D
DHX8
PRRC2C
GNL3L
PLEKHA6
TTK
SPAM1
HELZ
IPPK
CENPF
WDR67
SLCO4C1
UBE2T
LY75-CD302
UMODL1
MUM1L1
TNPO2
FBXO28
ACCSL
TES
GTPBP2
RTKN2
FREM1
PRPSAP2
SIX4
ZNF197
SYNGR4
KIAA0922
NOS1AP
RP11-831H9.16.1
CASP2
MTHFD2
C6orf223
DCAKD
NAA38
ATCAY
FAM55A
UNC13C
TPBG
PPA2
PKD1L1
LHX9
ANP32A
SMAD4
UBE2O
NEIL3

CEL
PYGO2
GRIK3
NIPSNAP1
ARMC9
PHB
THEG
KLHL28
FECH
INPP5E
NSUN4
PRDM4
TEX2
ATRX
SLC17A3
BYSL
FUBP1
TUFT1
RFC2
NEDD4
GPR125
KLHL20
USP3
HCLS1
ARHGAP11A
H2BFS
SHMT2
CLP1
KCTD10
SREK1
LRRC16A
GNRH1
RGS12
MAP1LC3C
PPP2R2B
VANGL1
RPAIN
HUWE1
IGSF3
SRRM1
ARL17A
CKM
ARMCX3
CHTF8
MED20
MAGEC3
DTYMK
HNRNPA1
SLC39A7
NCAPG

RDX
CDKN2D
CEP135
NR1I3
CDK13
NR1D2
SLC35B2
AIMP2
OR4A47
HOXC6
ZNF311
KCTD2
ABT1
ING3
DHRSX
LRFN3
FFAR3
CDK12
KDR
NUP85
TRIM5
GUCY1A3
SLC1A2
MYLIP
SSBP2
LSM14B
DPH1
EDDM3A
BIRC5
GRM4
AGXT2L1
USP47
SLC25A11
KRBA2
OPA1
INPP4B
AFP
C17orf80
TSG101
CHD4
HSPA8
ZFYVE1
TRIM24
FAM134A
EHD3
HES1
FRMD5
NFE2L1
KIAA0100
SMARCD1

RP11-528L24.3.1
PAF1
RACGAP1
MURC
WHSC2
PCDHA5
BZW2
NAA40
SCAND3
PCSK9
FAM55B
FAM123B
KRTAP10-3
SPOCK3
GPN2
LHFPL3
RAD21
IP6K3
FRK
DTNBP1
DSCR3
ZXDC
OR10G2
KRT33B
PRSS1
PHIP
KRTAP1-4
RALY
C12orf40
PROZ
FZD1
PKP3
SFXN1
ATXN7L1
PGM2L1
MTUS2
GPX6
CLVS2
RP11-463C8.4.1
XAGE5
HTR1E
LRRC53
OR2B11
OR51F2
AWAT1
OR4D11
KBTBD5
OR7E24
OR10P1
GHRH

OR1L3
OR10K2
MEPE
OR51S1
LGALS13
GPR148
PRSS48
CTD-2140B24.4.1
HSFY2
PTH
TMEM207
FABP12
PDHA2
NPBWR2
MAGEB1
DCAF8L1
GABRA6
MT4
BMP10
KRTAP12-1
C13orf44
ODF4
HTN3
KRTAP4-4
G6PC
RBMXL3
SYNE2
SYNE2
OR52R1
RP1-317E23.6.1
DPPA3
C7orf62
OR51G2
EBLN1
RP13-512J5.1.1
RP11-343C2.3.1
SPHKAP
C10orf126
USP26
ACCN5
PRM3
YIPF7
CCDC105
SLC13A2
CELA3A
C7orf34
POTED
OR51A7
ZFP64
GPR151

TAF1
PRKD1
MKI67
FMR1
SPAG5
HCFC1
HERC2
ZNF543
UTP18
C15orf63
DHX40
ZNF404
SI
IFI16
YLPM1
IMPG2
KRTAP2-2
HYAL4
BRDT
AMMECR1
LETM1
CCNB1
CDKN2AIP
KIAA1919
SESTD1
C2CD3
PARP1
RANGAP1
TAS2R38
PAGE1
CCDC109B
B3GAT1
ESPL1
RGS7BP
WNT10B
SMPD1
NAALADL2
PSMD2
RNFT2
RNF217
EIF4ENIF1
POLR2F
FAM18B2-CDRT4
DSG2
BAK1
SNRNP40
ZNF714
FXR1
ISX
MYH9

CTNNB1
SRBD1
GPSM2
PPP2R5D
PSMD10
RP11-529K1.3.1
DEFB4A
MLF1IP
ATXN7L3
SPP1
ZNF486
SLC24A5
DDX3X
SLC35A2
LDOC1
ST14
ARHGAP19
KCNJ1
HNRNPH1
PCDHA7
PABPC1L2A
LIG3
MCM7
DRD2
TUBG1
CTNNA2
EXT2
HELB
SEC24B
FBXO5
TRIM56
CTXN3
SLC25A2
C15orf44
BACH1
CENPL
GPRC6A
ZNF384
ZFYVE19
CDCA8
MID2
PPP1R37
POLDIP2
SNAPC5
PRPSAP1
ZBED6
SMUG1
KIAA0586
FAM106A
RAD54L

LRRC59
HIF1A
HIF1A
RIPK4
DLGAP1
PRR19
SLITRK6
GPRASP2
PAK1IP1
CCDC137
ZNF24
TMEM210
DICER1
RNF34
ZNF609
SNX13
HFE
DUSP12
RCC1
GLRB
DTNA
CAND1
PA2G4
UGT2B7
NARS
NUP188
GRSF1
PGK2
TNFAIP1
ZBED4
QSER1
C6orf106
TMEM89
SRC
CCNE1
NKIRAS2
TACC3
SMAD2
TMCC2
TOMM40L
KANSL2
RIC8B
TRIM61
C1orf111
SIKE1
CSNK1D
CKAP5
VPS53
SPTBN2
NUDT1

SLC35E2
HJURP
ZFP92
SCFD2
EIF4G1
PABPC1L2B
ADAM20
BRCC3
DLGAP5
LHX8
ABP1
DDX5
CDK16
SLFN11
TIMM21
SRP72
SENP5
DGKB
STK16
NAP1L4
TSTD2
CRAMP1L
GYPB
TMEM199
HOXC12
WBP11
PTPLAD1
SUV39H1
PHLDA2
OCRL
HECW2
RAP1GDS1
FOXK2
AP1S1
FOXO6
JMJD6
BAZ2A
MECP2
H2AFX
PCNA
EGLN3
SLC25A44
ZSCAN23
FN3KRP
ZNF331
SRSF1
CMAS
MGA
KIAA0317
F2RL1

FZD2
MAGED1
FBLN7
CWF19L2
LRP5L
FAT1
ZNF398
SLC15A2
VPS25
MTA2
GPRC5D
HNRNPH2
EYA1
SOX4
KRTAP2-1
ACVR2B
TACO1
LNX1
IMP3
UQCRFS1
ANAPC11
KIAA1549
XRCC2
SMG8
RAD51B
CDCA5
C11orf58
SVOP
SVOP
CPA1
MED24
HTR3A
DIS3L2
RRM1
ANP32E
RNASEH1
CDC7
DAND5
YTHDF1
TG
ACSL1
POLR1A
LIMK1
CORO2A
BPIFC
HSP90AA1
NIF3L1
RPRM
NFYA
BARX2

ATF6B
SYNJ2BP
RBMX2
ULK1
TK1
UGT2B15
SLFN5
ZNF746
COPS7B
ZNF184
SMARCA4
NUSAP1
CACNA1E
MYO1B
TEX19
NAP1L6
RIC8A
SLC25A5
ERV3-1
C14orf118
C6orf62
BRAF
RNF44
TRAFD1
TCN1
HMGB2
RBM12
PBK
AL137798.1
NCAPG2
SP4
AC069257.9.1
RP11-182J1.16.1
HBP1
IQGAP3
PEX14
CANT1
GNB1
CHST10
TTC19
SLC22A14
GRB10
ZNF232
TMEM206
MCM8
TMEM179
DPP3
EPHB3
FITM2
DNAJB6

HBM
RGPD1
CCDC47
TIPIN
CCDC144A
VPS37D
CABP4
C11orf46
FRS2
GALNT1
KRTAP5-1
CTR9
RAD17
HMGCR
PLXNA3
GEMIN2
PLEKHG3
TROVE2
JAK1
ARL13A
RPAP1
MAPKAPK5
NSF
CHRNA7
CHRNA7
TRMT2B
EXD1
RAD54L2
EML4
YWHAQ
AFM
HP1BP3
RP9
SLC25A15
KIFC1
C15orf42
BRCA1
HUS1B
FBXW11
EFTUD2
PAFAH1B1
FLVCR2.1
CHST6
DDX23
ZNF143
GRM3
ARHGAP11B
GSG2
E2F1
TWF2

CDCA3
APP
SLAMF9
RBM11
TRIM68
HOXB13
ZNF397
TBC1D22B
C9orf102
SCRN3
NFKBIL1
C11orf94
DISC1
GSTCD
XRCC5
PRR14L
TRAPPC10
AAMP
DCAF4
MDC1
OTUD7A
NUDCD3
MRPL10
IGSF1
CHST1
NUF2
MDH2
UBTF
FOXJ3
STYK1
WRNIP1
MCM3
LMNB1
DOCK8
ZNF641
AUTS2
ANKRD11
ARID1A
PDAP1
TMEM150C
LDB1
TPM1
GOLGA8H
SVIL
SEMA3A
FBXO3
USP49
LANCL3
MED1
HSPA6

FEN1
MRPS27
KIAA1267
TFCP2
CKAP2L
AP000721.4.1
ENTPD7
CNTN2
LGALS9
GTF2H1
ADCY5
SMARCD2
TNFRSF11B
KLHL23
MEX3A
FNBP4
BTBD10
MOB2
YTHDC1
SYT1
ZZZ3
C16orf59
LMBR1
SIX1
TIMELESS
ANKS1A
ZSCAN10
RGS16
SLC29A4
RP11-332O19.5.1
HNRNPR
AMPD3
PTGES2
PITPNM2
C12orf51
C17orf85
WDR82
SAAL1
FOXM1
TEAD2
HIRA
RNF216
PARVA
PIF1
RNF121
GRK6
ZNF669
FAM84A
MAP2K6
C20orf72

E2F3
DLG3
MICB
FAM5B
TMEM48
GTPBP1
NEO1
RBBP8
RBBP4
BUB1B
HSPA1B
SLC35G1
TCF12
PCDHA9
CSRP2BP
ERCC8
RGPD4
SPATA5
BBOX1
ZNF318
NEURL3
GZF1
BCL7A
SNRNP48
PFKFB3
B4GALNT4
CREB1
PLA2G3
TSPYL5
ARHGEF2
OR10A2
PSMD3
CCNB2
TJP1
RNF20
B3GAT3
GINS2
SEC23B
UGT2B11
GNAI3
MCPH1
SPECC1L
TRIP12
NEK6
IVNS1ABP
ATL1
AC078802.1
TSPAN12
UTP23
H2AFZ

ZNF827
RPA1
ACPL2
CXorf40B
RBP1
HSPA1A
CLDN24
TASP1
KDM6B
NAGK
TP53BP2
RNF220
WDHD1
RCC2
DMRTA2
DNAJB1
OR51Q1
GLYATL2
ISG20L2
WHSC1
ZNF343
VAX1
CNOT2
UBL4A
RP13-395E19.1.1
INCENP
NONO
HOXD12
SOCS3
C17orf64
KDM2A
KRTAP4-2
IGDCC3
IP6K2
PEX5
FAM59B
SLC19A2
ACLY
HARBI1
SLC4A11
ECT2
TGIF1
FZD5
AMBRA1
ABCG1
ANKRD32
AMMECR1L
BMF
LINGO1
WARS2

NUP153
GTF3C5
ZNF576
GADD45A
AACS
PHRF1
ZNF284
MCM6
43352
ADNP2
SIPA1L2
MAGED4
PRPS2
TNPO1
CENPI
CBX2
TAOK1
C9orf129
BAZ2B
BST1
SRSF3
MAGED4B
ZNF71
MFAP1
TRIM14
FAM193A
ZNF74
UBR1
TP53INP2
GPKOW
GNGT1
UBR7
USP32
MCM5
HDAC2
ZBTB22
MARK2
SSH2
POLA2
FSIP2
SPC24
SMC1A
KIF4A
SOX2
ZBTB39
IRF2BP2
GOSR2
DCAF4L1
FAM111B
CHMP4B

EME1
CADM2
NFATC3
APOBEC3B
LPIN1
DHX36
AAGAB
MGST3
MGST3
OBFC2B
MAD2L1BP
TMEM144
CAPRIN1
BARD1
CEP68
INO80
FRMD7
NUP98
C9orf69
RNF182
SOS2
SART3
SBF2
FAM78B
CD82
PTMA
TGFA
SRGAP2P2
POU4F3
ZADH2
PGBD1
ZNF648
EIF5
DCTN2
PRDM5
PJA1
BLMH
CASP3
USP37
GDAP1
LOH12CR1
LIN9
NECAP1
AVL9
TOLLIP
AGA
SPEN
NUMB
SLC25A25
SCN8A

ZSWIM6
ATG13
DNMT3A
SCYL2
GALNT8
OR2AE1
ZFHX4
CCNT1
DCLRE1B
IL19
TAF8
PIGS
SMAD5
SF3B3
ORC1
ATXN7L3B
RPS6KA6
TUBB
PGAP2
HOXC13
PRC1
CSRNP2
PPIF
DYNC1I2
NCAPH
ZNF711
POLR2D
SHPK
LRP8
SS18
FRAS1
NCL
FANCI
WNK2
ZWILCH
UHRF2
PPP1R12A
POLR2B
RBM14-RBM4
TAF5L
SRGAP2P1
LBX1
ORAI2
MBD1
ZSCAN12
GTSE1
METTL2A
MRPS18A
AURKB
C17orf53

KLHL2
PCDHA12
CALML5
EPG5
RNF41
CXorf38
GPANK1
CADM4
TRIM44
RTF1
RMI2
ATXN10
PKMYT1
ATP10B
SPOPL
JUN
PDF
MCM2
ROCK1
HNRNPM
H2AFV
CDC6
ZC3H10
TLK2
ZNF460
PPP1R8
KLHL24
KALRN
TMEM140
SLC16A14
FXC1
APH1A
MFSD2B
DTL
EIF4G2
E2F8
BRIP1
ATAD5
MAGEE1
LMTK2
RNF187
EPHB1
HDX
KPNB1
DEPDC1B
SYCP2L
RAD51
ADCY3
ZNF498
ZDHHC13

ZNF233
KIF23
CNO
C1orf112
RGS2
ANKRD36C
FBXO21
DVL3
SMARCC2
ATP2C1
ACTG1
PHF6
GPR161
ZNF391
ZNF142
WDR1
GSPT2
MAP4K4
AFAP1L2
NUP160
RSRC1
INSIG1
TOP2A
BCR
RCN1
C10orf12
CBX3
CCDC141
RBMX
ENOX2
C12orf44
KIF3C
MED17
CENPO
GLUL
C1orf52
NCOR1
CELF1
CELF1
KBTBD2
CNP
GMEB1
KIF18B
PRPF8
KPNA2
MINK1
RRM2
ABCB7
RPS6KB1
ZNF236

RBM15
NOL11
ASB8
ARIH1
ZNF407
PBRM1
ATRN
ZNF518B
CCNF
ZNF207
ARG2
SEMA4C
ZNF532
ZNF192
POLA1
CBX1
CS
PLEKHM3
UGT8
ATP7A
ANKRD52
DYRK2
